# Global, Regional, and National Burden of Caries Among Women of Childbearing Age From 1990 to 2021 and Prediction

**DOI:** 10.1016/j.identj.2026.109658

**Published:** 2026-06-17

**Authors:** Hui Chen, Meiling Hu, Longzhen Liu, Taohua Pan, Xi Chen, Jincai Guo

**Affiliations:** a Department of Pharmacy, Changsha Stomatological Hospital, Changsha, China; b School of Stomatology, Hunan University of Chinese Medicine, Changsha, China; c School of Pharmacy, Hunan University of Chinese Medicine, Changsha, China; d Department of Pharmacy, the Second Hospital of Zhuzhou City, Zhuzhou, China; e Department of Periodontics, Changsha Stomatological Hospital, Changsha, China; f Department of Endodontics, Changsha Stomatological Hospital, Changsha, China

**Keywords:** Caries, Women of childbearing age, Oral health, Global burden of disease study, Epidemiology, Trends

## Abstract

**Introduction and aims:**

Caries is a major global oral health issue. However, a critical gap persists in understanding its burden and temporal trends among women of childbearing age (WCBA). This study aims to analyse caries burden among WCBA from 1990 to 2021, project trends to 2040, and provide evidence for targeted oral health interventions.

**Methods:**

Data from the Global Burden of Disease study 2021 were used to assess caries burden among WCBA, including incidence, prevalence, and disability-adjusted life-years (DALYs). Temporal trends were analysed using joinpoint regression, associations with the sociodemographic index(SDI) were examined via Spearman correlation analyses, and future trends were projected using the Nordpred model.

**Results:**

In 2021, caries among WCBA resulted in 817.5 million incident cases and 688,430 DALYs globally, with South Asia recording the highest numbers of both indicators. The highest age-standardized incidence rate (ASIR) and age-standardized DALYs rate (ASDR) were observed in Tropical Latin America and Andean Latin America, respectively. From 1990 to 2021, the global ASIR increased (AAPC = 0.27%), whereas the ASDR declined modestly (AAPC = –0.03%). Both incidence and DALYs peaked in the 20–24 age group. Projections from 2022 to 2040 suggest that the ASIR will rise by 2.83%, while the ASDR is expected to decrease by 5.00%.

**Conclusion:**

Despite modest declines in ASDR, the rising ASIR, particularly in lower-SDI regions, underscores caries among WCBA as a persistent public health challenge requiring targeted interventions.

**Clinical Relevance:**

Caries among WCBA affects not only oral health but also systemic conditions, particularly metabolic disorders, adverse pregnancy outcomes, and offspring oral health. Assessing WCBA as a whole, as conducted in this study, provides an important basis and proxy for evaluating maternal oral health needs. Therefore, early screening and prevention should be prioritized in oral and primary care settings for young women and integrated into prenatal and maternal health programs to improve maternal and child health outcomes.

## Introduction

Caries is a major global health issue. The Global Burden of Disease Study (GBD) 2019 reported that there were over 3 billion new cases of caries in permanent teeth.^1^ The burden of caries extends beyond oral health, as it is linked to various systemic conditions. While associations with depression and cardiovascular disease are inconsistent, links with metabolic disorders are more consistently supported.^2^^,^^3^ Across all age groups, women exhibited a higher prevalence of caries and years lived with disability (YLD) rates than men. The risk factors for women include salivary composition, hormonal changes, diet, genetics, and social roles.^4^ Women of childbearing age (WCBA, 15-49 years) are especially vulnerable, as caries is the second most common oral disease in this group. For WCBA, potential impacts on pregnancy and birth outcomes are of particular concern. For example, caries shows no clear association with pre-eclampsia but is linked to large-for-gestational-age infants.^5^ These findings underscore the importance of assessing the burden of caries in WCBA to inform global public health strategies.

Previous studies on caries using GBD data have mainly described overall epidemiological patterns and trends across age groups.^6^^,^^7^ However, detailed burden assessments in specific populations remain limited. Existing population-specific studies have primarily focused on children and adolescents,^8^, ^9^, ^10^ while the burden among WCBA remains largely overlooked. Notably, GBD 2019 reported the high prevalence and incidence rates of caries in permanent teeth among individuals aged 15 to 49 years,^1^ underscoring the significance of studying this specific population. Two studies using data from the 1999-2004 National Health and Nutrition Examination Survey (NHANES) have begun to address this knowledge gap: one examined the impact of tobacco smoke on WCBA's oral health, including caries,^11^ and another compared the prevalence of caries between pregnant and non-pregnant WCBA.^12^ However, the geographic limitations of these studies hinder their global applicability. To address this knowledge gap, this study utilizes data from the GBD 2021 to analyse the burden of caries among WCBA from 1990 to 2021. Furthermore, the Nordpred age-period-cohort (APC) model is employed to project future trends from 2022 to 2040. The overarching objective of this research is to provide a comprehensive assessment of the caries burden among WCBA to inform targeted oral health interventions.

## Methods

### Data sources

The GBD 2021 provides the estimates for 371 diseases and injuries and 88 risk factors across 21 GBD regions and 204 countries/territories, including caries.^13^ The methodological details of the study process and estimation are described in the GBD 2021 publications.^13^ Data on incidence, prevalence, and disability-adjusted life-years (DALYs) for caries among WCBA from 1990 to 2021 were extracted from the Global Health Data Exchange GBD Results Tool (https://vizhub.healthdata.org/gbd-results/). Additionally, the sociodemographic index (SDI)—which reflects socioeconomic and demographic factors—was used to categorize national development into five quintiles (low, low-middle, middle, high-middle, and high), with values ranging from 0 to 1. The GBD dataset includes SDI values and corresponding quintiles for all GBD regions and countries.^13^ This study adheres to the Guidelines for Accurate and Transparent Health Estimates Reporting (GATHER).^14^ Since this study utilized publicly available data, it was exempt from ethical review and informed consent.

### Definition of caries among WCBA

In the GBD 2021, caries were defined as “teeth with unmistakable coronal cavity at dentin level, root cavity in cementum that feels soft or leathery to probing.”^15^ These burden estimates specifically reflect untreated dental caries (active decay).^15^ The corresponding International Classification of Diseases (ICD) version 10 codes were K02-K02.9, and the ICD-9 codes were 521.0-521.09.^13^

According to the World Health Organization (WHO) definition for WCBA,^16^ this study includes women aged 15-49 years and stratifies them into 7 age groups: 15-19, 20-24, 25-29, 30-34, 35-39, 40-44, and 45-49 years. Notably, while GBD 2021 classifies caries as encompassing both primary and permanent teeth, data on primary teeth are only available for individuals up to 14 years. Since this study focuses on women, aged 15-49, the term "caries" herein refers exclusively to caries of permanent teeth.

### Statistical analysis

#### Descriptive analysis

The burden of caries among WCBA was assessed by location and age, using the number and age-standardized rate (ASR) of incidence, prevalence, and DALYs as metrics. All rates are expressed per 100,000 population. The ASR (per 100,000 population) was calculated using the following formula: ASR = $\frac{\sum_{i=1}^{A}a_{iw_{i}}}{\sum_{i=1}^{A}w_{i}}\times \;100,000$, where a_i_ represents the age-specific rate in the ith age group, and w_i_ denotes the count of individuals within the same age group according to the GBD 2021 standard population.^13^

#### Trend analysis

Analysing temporal trends in disease burden provides essential insights for developing tailored interventions. Joinpoint regression is a commonly used statistical tool for this purpose.^17^ Its core principle involves identifying statistically significant change points (i.e., joinpoints) in time-series data through the Grid Search Method and permutation tests, while quantifying the annual percent change (APC) and its 95% confidence interval (CI) for each segment.^17^ The average annual percent change (AAPC) is further calculated to comprehensively evaluate the overall temporal trend. This model has been validated in numerous studies for its effectiveness in revealing segment-specific characteristics of disease trends and is broadly applied in epidemiological time-trend analyses.^18^, ^19^, ^20^, ^21^ In this study, joinpoint regression was employed to analyse the temporal trends in the ASR of caries among WCBA. The trend was interpreted as follows: if both the AAPC and its 95% CI are > 0 (or both < 0), it indicates an upward (or a downward) trend; otherwise, the trend is considered stable. To evaluate the robustness of temporal trend estimates to data fluctuations, a sensitivity analysis was performed using the upper and lower bounds of the dataset.

#### Correlation analysis

The Spearman correlation coefficient was employed to analyse the correlations of the SDI with both the ASR and the AAPC of caries among WCBA across 204 countries/territories. As a nonparametric method, this approach offers robustness to non-normal data distributions and outliers—a feature particularly suited to global caries burden data across diverse regions.^22^ Furthermore, the method quantifies monotonic associations between ordinal or continuous variables without linearity assumptions,^22^ supporting its widespread use in epidemiological correlation analyses.^23^, ^24^, ^25^

#### Predictive analysis

To predict future trends in the global burden of caries among WCBA, the Nordpred APC model was utilized. Based on generalized linear models, this model estimates the independent effects of age, period, and birth cohort on epidemiological indicators.^26^ The Nordpred APC model has been widely applied in forecasting trends in incidence, mortality, and disease burden for conditions such as cancer and chronic diseases, demonstrating strong predictive performance.^27^, ^28^, ^29^, ^30^ Using historical data from 1990 to 2021, we projected the global burden of caries among WCBA from 2022 to 2040, including the number and ASR of incidence, prevalence, and DALYs, stratified by age group. The projected population data for these projections were sourced from the 2024 revision of the World Population Prospects.^31^ A sensitivity analysis using the upper and lower bounds of the dataset was conducted to assess the robustness of future burden projections to potential data fluctuations.

All statistical analyses and visualizations were performed using R software (version 4.4.1), with the "Nordpred" package for predictive modelling and the "ggplot2" package for data visualization. Statistical significance was defined as a *p*-value < 0.05.

## Results

### Global and SDI regional level

**Fig. 1:**
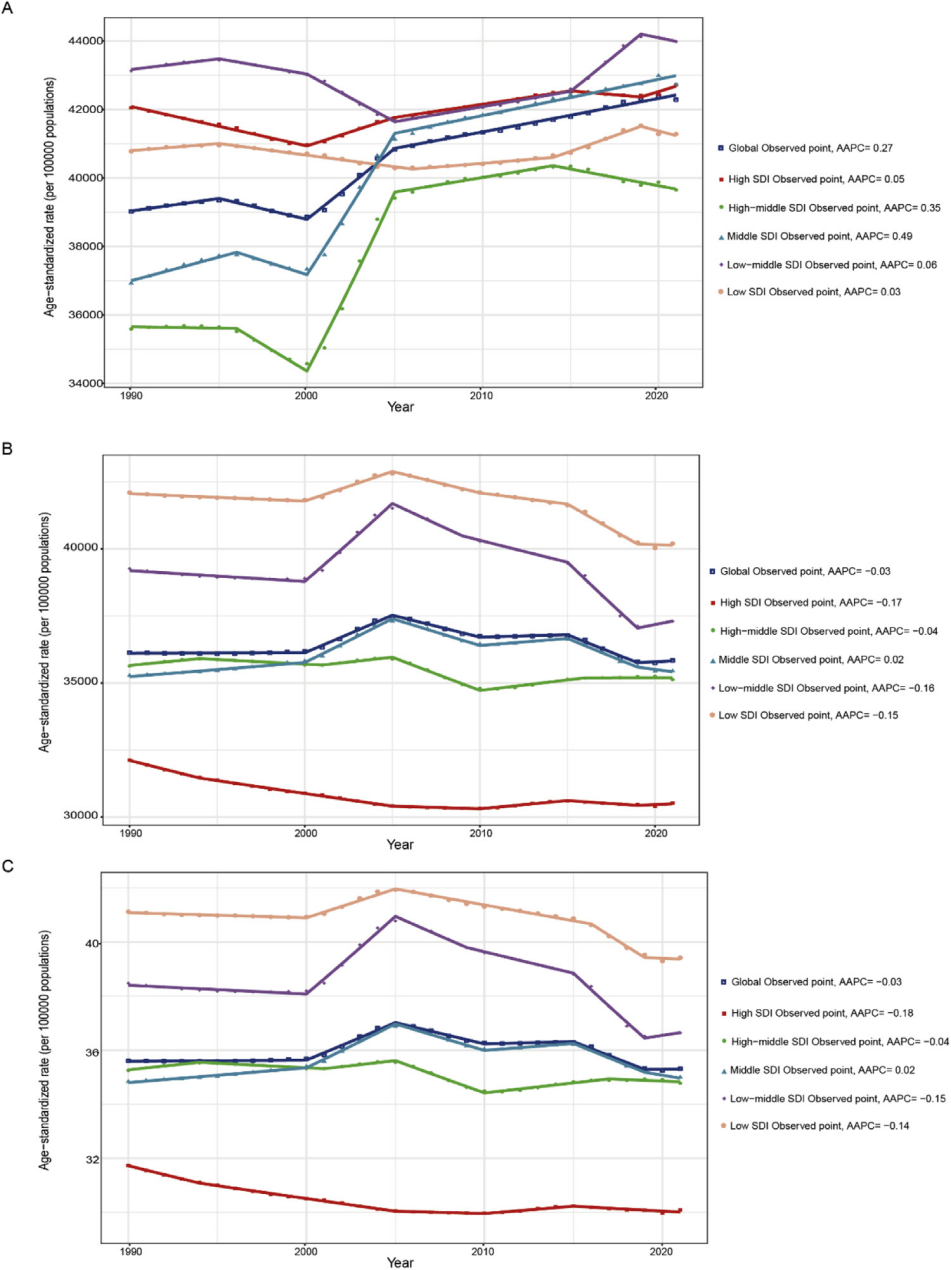
The joinpoint regression analysis of the ASR of incidence (A), prevalence (B), and DALYs (C) of caries among WCBA at the global and SDI regional level. ASR: age-standardized rate. DALYs: disability-adjusted life-years. SDI: sociodemographic index. WCBA: women of childbearing age.

In 2021, global estimates indicated 817,480,400 (95% uncertainty interval (UI): 728,746,610-931,295,527) incident cases, 699,003,728 (95% UI: 581,558,796-860,446,978) prevalent cases, and 688,430 (95% UI: 291,890-1,322,813) DALYs of caries among WCBA. From 1990 to 2021, the absolute number of incident cases, prevalent cases, and DALYs all increased by 53.30%, 44.57%, and 44.32%, respectively. During this period, the age-standardized incidence rate (ASIR) showed a significant upward trend, with an AAPC of 0.27%. In contrast, both the age-standardized prevalence rate (ASPR) and age-standardized DALYs rate (ASDR) exhibited modest declines, each with an AAPC of -0.03% (Fig. 1; Table 1, Table 2, Table 3).

**Table 1:** Number of cases and ASR of caries incidence among WCBA in 1990 and 2021 at global, SDI, and GBD regional level, with AAPC from 1990 to 2021.

Location	1990		2021		AAPC (95%CI) 1990–2021	*P*
	Number (95%UI)	ASR (95%UI)	Number (95%UI)	ASR (95%UI)		
Global	533258025 (470567962-609832943)	39022 (30290.14-47905.03)	817480400 (728746610-931295527)	42286.71 (33197.09-51442.32)	0.27 (0.26 to 0.28)	<0.001
**SDI quintiles**						
High SDI	94259606 (84680792-104806278)	42050.48 (34426.24-49612.08)	99679094 (90242833-111232515)	42720.07 (35059.88-50264.41)	0.05 (0.04 to 0.05)	<0.001
High-middle SDI	100276931 (87289463-116571548)	35587.62 (27220.14-44488.05)	114837279 (101287635-134507800)	39650.38 (30345.82-49453)	0.35 (0.32 to 0.37)	<0.001
Middle SDI	169914845 (147845794-197251449)	36925.68 (28010.91-46193.41)	259691538 (229097808-296903466)	42725.99 (33250.72-52273.7)	0.49 (0.47 to 0.5)	<0.001
Low-middle SDI	121058994 (105896258-137872011)	43127.2 (32829.94-53283.45)	225302589 (199174980-255650051)	43983.97 (34183.54-53651.44)	0.06 (0.05 to 0.07)	<0.001
Low SDI	47252851 (42060301-53841571)	40776.48 (32133.27-49999.34)	117350092 (104228775-132850640)	41282.43 (32599.62-50244.92)	0.03 (0.03 to 0.04)	<0.001
**GBD regions**						
Andean Latin America	3679589 (2991590-4491557)	37961.25 (25934.78-51402.19)	6785182 (5525008-8228332)	38770.22 (26565.22-52158.28)	0.05 (0.03 to 0.07)	<0.001
Australasia	1964170 (1665197-2349374)	36621.01 (27020.67-47246.19)	2993500 (2579258-3541387)	42572.45 (31673.1-54472.33)	0.51 (0.49 to 0.53)	<0.001
Caribbean	3832965 (3266762-4504872)	40170.58 (30091.16-51157.98)	4901971 (4250185-5663911)	40947.31 (32050.02-50348.03)	0.06 (0.05 to 0.07)	<0.001
Central Asia	6843156 (5621399-8252750)	39735.56 (28053.42-52530.79)	9794307 (8042116-11948957)	40725.32 (28984.23-53392.04)	0.07 (0.06 to 0.08)	<0.001
Central Europe	11489580 (9746380-13643883)	37712.89 (27817.35-48828.32)	9917716 (8501076-11682002)	40080.95 (30002.79-50792.16)	0.23 (0.21 to 0.24)	<0.001
Central Latin America	18572486 (16086175-21402481)	42609.27 (32065.98-53206.67)	29171086 (25575324-33880493)	42903.69 (32614.43-53272.66)	0.03 (-0.01 to 0.05)	0.074
Central Sub-Saharan Africa	5197341 (4408094-6305350)	40149.37 (28889.33-52488.12)	13526018 (11512479-16021052)	39830.99 (28638.72-52251.69)	-0.03 (-0.05 to -0.02)	0.003
East Asia	95003740 (79705563-113531676)	27584.18 (21015.72-35694.04)	111989664 (96440198-130883874)	36391.27 (27878.08-45863.95)	0.9 (0.83 to 0.97)	<0.001
Eastern Europe	24505063 (21022397-28326963)	44662.35 (33438.47-55572.04)	20661582 (17679803-23954107)	45050.62 (34149.88-55595.67)	0.03 (0.03 to 0.03)	<0.001
Eastern Sub-Saharan Africa	17523100 (15517871-20077943)	38708.58 (30408.62-47804.57)	44294819 (38920307-50489175)	39526.29 (31116.77-48394.23)	0.06 (0.05 to 0.07)	<0.001
High-income Asia Pacific	20512702 (18454821-22564921)	45639.62 (37178.1-53685.67)	14915869 (13289003-16509223)	42239.2 (34482.11-49519.78)	-0.25 (-0.26 to -0.23)	<0.001
High-income North America	33455306 (29312021-37877963)	45490.47 (35993.19-55400.8)	37440140 (33039772-41775633)	45522.13 (36507.29-54657.46)	-0.01 (-0.03 to 0.01)	0.137
North Africa and Middle East	32862918 (28182716-38114516)	41062.94 (30456.29-52000.11)	66240716 (57285420-77644657)	41636.27 (30850.26-52623.57)	0.05 (0.04 to 0.05)	<0.001
Oceania	572124 (458515-717989)	35708.35 (24459.44-49193.84)	1257161 (1002995-1568109)	35724.86 (24642.77-48104.92)	0.01 (-0.02 to 0.02)	0.585
South Asia	114670833 (100452410-130881889)	43833.74 (33643.1-54180.01)	225436025 (199766149-254567204)	45190.21 (35712.45-54659.41)	0.09 (0.07 to 0.11)	<0.001
Southeast Asia	56867434 (49097927-64681027)	46204.63 (34728.27-56886.12)	85505905 (75190189-97414385)	46963.26 (36351.98-57062.63)	0.05 (0.04 to 0.06)	<0.001
Southern Latin America	4889747 (3997451-5745220)	39125.17 (27046.25-52130.11)	6886074 (5909417-7993217)	40077.15 (30060.49-50748.02)	0.03 (0 to 0.07)	0.045
Southern Sub-Saharan Africa	5620335 (4925972-6424225)	40246.6 (31126.1-49982.35)	8910989 (7856783-10325579)	40797.14 (31704.56-50757.89)	0.04 (0.02 to 0.05)	<0.001
Tropical Latin America	19023758 (16200899-21716404)	46904.34 (34680.73-58139.65)	28887213 (24758457-33009965)	48113.52 (36165.64-59019.91)	0.08 (0.07 to 0.09)	<0.001
Western Europe	36954517 (32626982-41728486)	39154.43 (30910.24-47366.56)	36279314 (32036521-41270248)	41142.24 (32618.18-49575.27)	0.15 (0.15 to 0.16)	<0.001
Western Sub-Saharan Africa	19217161 (17143317-21744574)	41732.79 (33231.72-50318.63)	51685150 (45929079-58258153)	41071.57 (32978.83-49408.21)	-0.05 (-0.06 to -0.05)	<0.001

**Table 2:** Number of cases and ASR of caries prevalence among WCBA in 1990 and 2021 at global, SDI, and GBD regional level, with AAPC from 1990 to 2021

Location	1990		2021		AAPC (95%CI) 1990–2021	*P*
	Number (95%UI)	ASR (95%UI)	Number (95%UI)	ASR (95%UI)		
Global	483507119 (397021524-599828379)	36137.85 (25082.92-49578.9)	699003728 (581558796-860446978)	35842.43 (25611.86-48343.59)	-0.03 (-0.04 to -0.02)	<0.001
**SDI quintiles**						
High SDI	72999620 (60312869-89885543)	32126.41 (23327.82-43436.08)	74302514 (63022411-89783340)	30522.2 (22947.59-40049.48)	-0.17 (-0.17 to -0.16)	<0.001
High-middle SDI	99182786 (78925101-123983214)	35637.09 (23433.76-50026.78)	107231788 (87321255-133263935)	35127.12 (24589.21-47967.03)	-0.04 (-0.05 to -0.03)	<0.001
Middle SDI	158332100 (126903713-197269909)	35276.6 (23877.82-49244.26)	219523176 (180568080-272536200)	35448.36 (24916.6-48474.15)	0.02 (0 to 0.03)	0.032
Low-middle SDI	106063922 (87782464-130196490)	39264.09 (27343.75-53335.28)	188435742 (155606163-233231465)	37301.63 (26214.94-50807.43)	-0.16 (-0.17 to -0.14)	<0.001
Low SDI	46351256 (39394379-55216020)	42102.79 (30982.04-54269.08)	108849254 (92144007-130739584)	40199.22 (29484.47-52566.64)	-0.15 (-0.16 to -0.14)	<0.001
**GBD regions**						
Andean Latin America	4868685 (3994452-5852767)	51871.24 (35847.31-67408.66)	8877047 (7325090-10624515)	50764.09 (34679.62-66765.12)	-0.05 (-0.08 to -0.03)	<0.001
Australasia	2159205 (1783010-2585584)	40089.74 (28061.59-53818.76)	2510483 (1925628-3250882)	34440.15 (21975.3-49768.66)	-0.49 (-0.52 to -0.46)	<0.001
Caribbean	4476773 (3695135-5367389)	48464.93 (33303.59-63607.55)	5431957 (4713474-6302906)	44985.87 (33925.36-56131.82)	-0.23 (-0.25 to -0.22)	<0.001
Central Asia	7965072 (6520039-9782964)	47648.69 (32233.07-63298.15)	11374505 (9376800-13983274)	46521.91 (31510.46-62347.39)	-0.08 (-0.08 to -0.07)	<0.001
Central Europe	15339759 (13008278-17997812)	49960.3 (36135.39-63278.03)	12005284 (10196214-14283616)	46224.22 (33168.73-59700.03)	-0.26 (-0.27 to -0.26)	<0.001
Central Latin America	15922953 (13076747-19819746)	38541.18 (26290.79-53348.78)	25427495 (20806739-31333193)	37184.82 (25464.07-51424.04)	-0.16 (-0.24 to -0.11)	<0.001
Central Sub-Saharan Africa	5120735 (4106879-6300106)	42089.4 (27495.79-58714.68)	13548971 (10909488-16828009)	42116.99 (27353.62-58785.27)	0.01 (-0.01 to 0.03)	0.156
East Asia	92914341 (70101910-122882608)	27042.48 (16921.16-40670.33)	89969869 (71774404-116049466)	27843.42 (19048.66-40013.85)	0.11 (0.09 to 0.14)	<0.001
Eastern Europe	22375783 (18258352-27774213)	40170.77 (27433.33-55009.36)	19354691 (15802113-23973633)	39270.09 (27159.42-53561.14)	-0.08 (-0.09 to -0.07)	<0.001
Eastern Sub-Saharan Africa	18494738 (15833533-21668379)	43720.49 (32965.05-54986.58)	43054599 (36383409-51193783)	40896.3 (30003.27-52639.7)	-0.22 (-0.23 to -0.21)	<0.001
High-income Asia Pacific	10944127 (9065630-13831194)	24333.5 (17851.52-33660.65)	7693843 (6452827-9398673)	21547.09 (16129.66-29128.91)	-0.39 (-0.41 to -0.37)	<0.001
High-income North America	20179184 (15779245-25520170)	27125.32 (18444.11-39165.24)	21819402 (17692682-27107691)	26089.54 (18604.35-36574.22)	-0.12 (-0.13 to -0.11)	<0.001
North Africa and Middle East	33185495 (27359715-40526700)	42832.04 (30204.84-56968.27)	66609687 (54911197-81911656)	41761.8 (28818.33-56491.89)	-0.08 (-0.09 to -0.07)	<0.001
Oceania	769340 (618346-950835)	49692.36 (33040.79-66431.21)	1702998 (1381378-2093315)	49084.88 (33228.87-65044.9)	-0.03 (-0.04 to -0.02)	<0.001
South Asia	101504512 (84250256-124545038)	40298.78 (28397.29-54437.32)	186004756 (153678024-229098730)	37714.93 (27012.22-50972.53)	-0.22 (-0.25 to -0.19)	<0.001
Southeast Asia	46022481 (37669413-57205555)	38584.65 (26244.69-53382.45)	67017932 (54892745-83135378)	36458.23 (25321.35-50367.19)	-0.2 (-0.22 to -0.18)	<0.001
Southern Latin America	6039351 (4963526-7301811)	49018.59 (33576.39-64645.95)	8567590 (7448646-9860618)	48552.83 (36733.51-60344.81)	-0.02 (-0.05 to 0.01)	0.131
Southern Sub-Saharan Africa	4797481 (3921323-5907377)	36939.43 (25693.2-50321.46)	7472992 (6024461-9473092)	34376.74 (23602.67-47895.14)	-0.22 (-0.24 to -0.2)	<0.001
Tropical Latin America	15552089 (12651766-19374758)	39186.53 (26302.57-54766.12)	23663890 (19390974-29347212)	38739.21 (26474.48-53700.18)	-0.04 (-0.06 to -0.02)	0.002
Western Europe	39229386 (32651201-47930167)	40747.11 (28675.63-54715.28)	36178114 (30869190-43108431)	37984.95 (28194.46-48741.07)	-0.23 (-0.23 to -0.22)	<0.001
Western Sub-Saharan Africa	15645631 (13000965-19121020)	35978.79 (25832.7-48082.84)	40717625 (33895316-49833112)	33989.93 (24671.92-45198.52)	-0.21 (-0.22 to -0.2)	<0.001

**Table 3:** Number of cases and ASR of caries DALYs among WCBA in 1990 and 2021 at global, SDI, and GBD regional level, with AAPC from 1990 to 2021.

Location	1990		2021		AAPC (95%CI) 1990–2021	*P*
	Number (95%UI)	ASR (95%UI)	Number (95%UI)	ASR (95%UI)		
Global	477001 (198864-923926)	35.61 (14.22-70.45)	688430 (291890-1322813)	35.31 (14.22-69.92)	-0.03 (-0.04 to -0.02)	<0.001
**SDI quintiles**						
High SDI	72069 (30706-139939)	31.74 (12.85-62.92)	73136 (31739-142740)	30.09 (12.48-59.97)	-0.18 (-0.19 to -0.17)	<0.001
High-middle SDI	98174 (40197-188505)	35.25 (13.82-70.22)	105928 (44840-205698)	34.77 (13.85-69.35)	-0.04 (-0.05 to -0.03)	<0.001
Middle SDI	156656 (63789-300380)	34.84 (13.8-69.48)	216509 (91454-417969)	34.99 (13.96-69.91)	0.02 (0-0.02)	0.060
Low-middle SDI	104154 (43924-202192)	38.47 (15.34-76.26)	185223 (77828-357219)	36.64 (14.53-72.23)	-0.15 (-0.17 to -0.14)	<0.001
Low SDI	45378 (19377-85434)	41.12 (16.99-80.51)	106982 (46027-201770)	39.42 (16.32-77.62)	-0.14 (-0.15 to -0.13)	<0.001
**GBD regions**						
Andean Latin America	4812 (2042-9023)	51.16 (20.88-100.85)	8759 (3732-16663)	50.09 (20.39-99.04)	-0.06 (-0.08 to -0.03)	<0.001
Australasia	2120 (887-4070)	39.37 (15.7-77.85)	2465 (1011-4693)	33.86 (13.03-68.29)	-0.49 (-0.52 to -0.47)	<0.001
Caribbean	4412 (1875-8399)	47.71 (19.76-93.75)	5337 (2362-10001)	44.21 (18.65-85.85)	-0.24 (-0.25 to -0.23)	<0.001
Central Asia	7886 (3372-15229)	47.11 (19.17-93.72)	11235 (4786-21662)	45.98 (18.83-91.15)	-0.08 (-0.08 to -0.07)	<0.001
Central Europe	15178 (6529-28870)	49.46 (20.67-96.9)	11863 (5119-22449)	45.79 (18.96-90.63)	-0.25 (-0.26 to -0.25)	<0.001
Central Latin America	15740 (6721-30083)	38.02 (15.17-75.85)	25065 (10671-48429)	36.66 (14.61-73.1)	-0.16 (-0.25 to -0.12)	0.002
Central Sub-Saharan Africa	4992 (2082-9580)	40.91 (16.15-82.5)	13289 (5468-25862)	41.21 (16.14-84.05)	0.03 (0.02 to 0.05)	<0.001
East Asia	92438 (37014-176903)	26.87 (10.16-55.44)	89422 (37383-174623)	27.73 (10.85-56.99)	0.12 (0.09 to 0.14)	<0.001
Eastern Europe	22085 (9266-43090)	39.68 (15.88-79.23)	19055 (8110-36756)	38.77 (15.43-77.39)	-0.08 (-0.09 to -0.07)	<0.001
Eastern Sub-Saharan Africa	18133 (7774-34762)	42.75 (17.88-82.84)	42375 (18448-81022)	40.15 (16.7-78.68)	-0.2 (-0.21 to -0.2)	<0.001
High-income Asia Pacific	10872 (4643-21475)	24.18 (9.78-48.53)	7643 (3334-14889)	21.44 (8.84-42.84)	-0.39 (-0.41 to -0.37)	<0.001
High-income North America	19902 (8505-38502)	26.77 (10.38-54.06)	21400 (9250-41933)	25.61 (10.31-52.07)	-0.14 (-0.15 to -0.13)	<0.001
North Africa and Middle East	32668 (13845-63192)	42.06 (17.1-83.87)	65362 (27558-126475)	40.99 (16.69-81.47)	-0.08 (-0.09 to -0.08)	<0.001
Oceania	760 (313-1452)	48.98 (19.44-96.43)	1681 (732-3194)	48.42 (19.12-96.48)	-0.03 (-0.05 to -0.02)	<0.001
South Asia	99458 (41691-190150)	39.41 (15.72-78.02)	182551 (76721-346827)	36.99 (14.8-72.75)	-0.21 (-0.24 to -0.18)	<0.001
Southeast Asia	45618 (19062-87969)	38.19 (15.34-76.07)	66375 (27755-129913)	36.12 (14.35-72.02)	-0.19 (-0.21 to -0.18)	<0.001
Southern Latin America	5950 (2499-11328)	48.27 (19.74-94.08)	8410 (3685-16033)	47.7 (20.28-91.94)	-0.04 (-0.07 to -0.01)	0.009
Southern Sub-Saharan Africa	4715 (1987-9007)	36.22 (14.61-72.97)	7294 (3060-13967)	33.55 (13.4-67.34)	-0.25 (-0.27 to -0.23)	<0.001
Tropical Latin America	15276 (6317-29603)	38.43 (15.21-76.64)	23169 (9721-44666)	37.97 (15.11-75.87)	-0.04 (-0.07 to -0.02)	0.001
Western Europe	38627 (16273-74130)	40.14 (16.19-79.48)	35566 (15564-68325)	37.39 (15.43-73.94)	-0.23 (-0.24 to -0.22)	<0.001
Western Sub-Saharan Africa	15360 (6547-29245)	35.22 (14.34-69.75)	40114 (17041-75655)	33.41 (13.54-66.3)	-0.2 (-0.21 to -0.19)	<0.001

Across the five SDI quintiles, the highest numbers of incident cases, prevalent cases, and DALYs due to caries among WCBA in 2021 were recorded in the middle SDI regions, while the high SDI regions had the lowest. In terms of ASR, the low-middle SDI regions showed the highest ASIR, whereas the high-middle SDI region had the lowest. The highest ASPR and ASDR were observed in the low SDI regions, and the lowest values for both measures were found in the high SDI regions. From 1990 to 2021, the most substantial increases in ASIR, ASPR, and ASDR occurred in the middle SDI regions. Conversely, the low SDI regions experienced the greatest decline in ASIR, and the high SDI regions showed the largest decrease in ASPR and ASDR (Fig. 1; Table 1, Table 2, Table 3).

Sensitivity analyses modelling the lower and upper bounds of the data showed similar trends, confirming the robustness of the trend analysis (Supplementary Table 1).

### GBD regional and national levels

Regionally, South Asia recorded the highest number of incident cases, prevalent cases, and DALYs for caries among WCBA in 2021, while Oceania reported the lowest (Table 1, Table 2, Table 3). Tropical Latin America showed the highest ASIR, whereas Andean Latin America had the highest ASPR and ASDR. Oceania exhibited the lowest ASIR, and High-income Asia Pacific had the lowest ASPR and ASDR. Between 1990 and 2021, East Asia experienced the most substantial increases in ASIR, ASPR, and ASDR. In contrast, the largest decrease in ASIR occurred in High-income Asia Pacific, while Australasia saw the greatest declines in both ASPR and ASDR (Table 1, Table 2, Table 3).

At the national level, India had the highest number of incident cases, prevalent cases, and DALYs in 2021, followed by China; Tokelau reported the lowest counts for these three indicators. The highest ASIR was observed in the Republic of Korea, while Madagascar showed the highest ASPR and ASDR. Taiwan (Province of China) had the lowest ASIR and ASDR, and Japan recorded the lowest ASPR (Supplementary Tables 2-4). From 1990 to 2021, China experienced the most substantial increase in ASIR, while Japan saw the largest decrease. Denmark exhibited the greatest rises in both ASPR and ASDR over this period, whereas Mozambique showed the most notable declines in both metrics (Supplementary Tables 2-4).

### Global burden by age group

Globally, the age distribution of incidence, prevalence, and DALYs for caries among WCBA was largely consistent. In 2021, the highest counts of incident cases, prevalent cases, and DALYs occurred in the 20-24 age group, while the lowest values for these three indicators were observed in the 45-49 age group. Similarly, the highest ASIR was also found in the 20-24 age group, and the lowest in the 45-49 age group. The highest ASPR and ASDR were also recorded in the 20-24 age group; however, the lowest ASPR and ASDR were seen in the 15-19 age group (Supplementary Table 5). From 1990 to 2021, ASIR showed an upward trend across all age subgroups, with the most pronounced AAPC occurring in the 15-19 age group. In contrast, both the ASPR and ASDR decreased significantly in most age subgroups, with the most pronounced decline in the 20-24 age group. (Supplementary Fig. 1).

### Correlation between the SDI and the burden

**Fig. 2:**
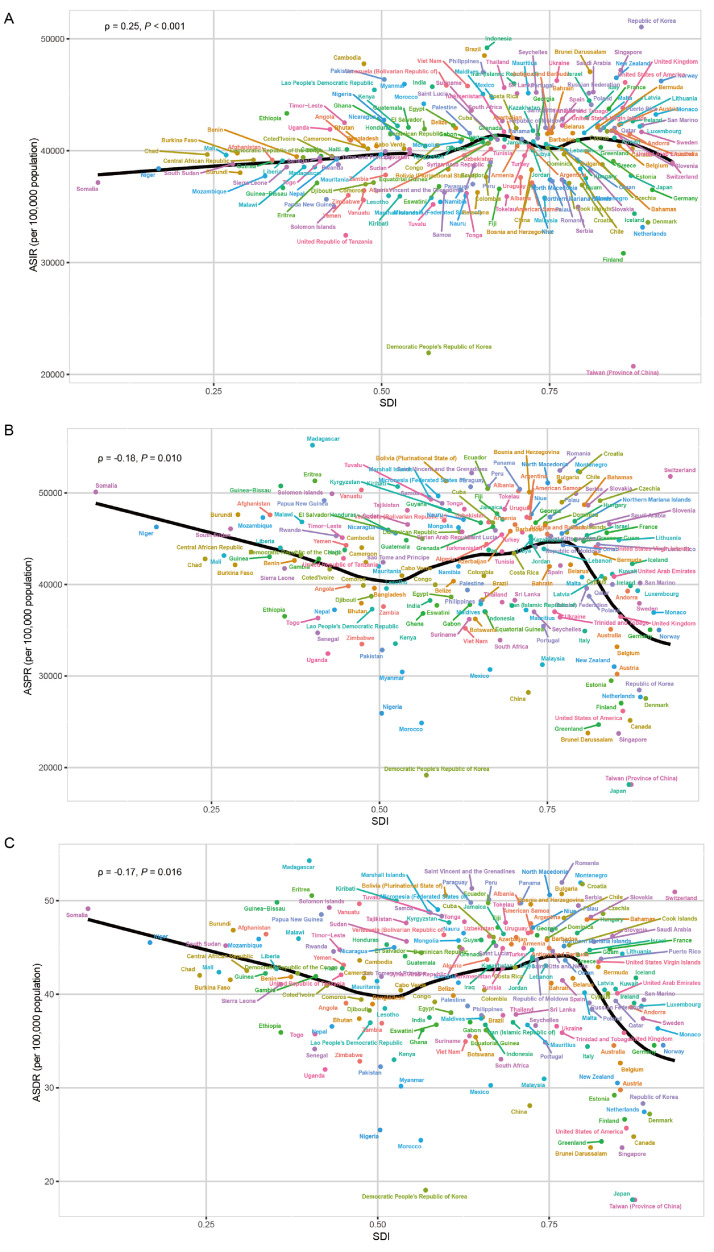
The correlation between the SDI and the ASIR (A), ASPR (B), and ASDR (C) of caries among WCBA in 2021. ASIR: age-standardized incidence rate. ASPR: age-standardized prevalence rate. ASDR: age-standardized DALYs rate. DALYs: disability-adjusted life-years. SDI: sociodemographic index. WCBA: women of childbearing age.

A slight positive correlation was observed between the SDI and the ASIR of caries among WCBA across 204 countries and territories (ρ = 0.25, *P* < 0.001). In contrast, weak negative correlations were found between SDI and both the ASPR (ρ = -0.18, *P* = 0.01) and ASDR (ρ = -0.17, *P* = 0.016) (Fig. 2). The correlation between SDI and AAPC followed a similar pattern but was stronger in magnitude (Supplementary Fig. 2).

### Global burden prediction to 2040

**Fig. 3:**
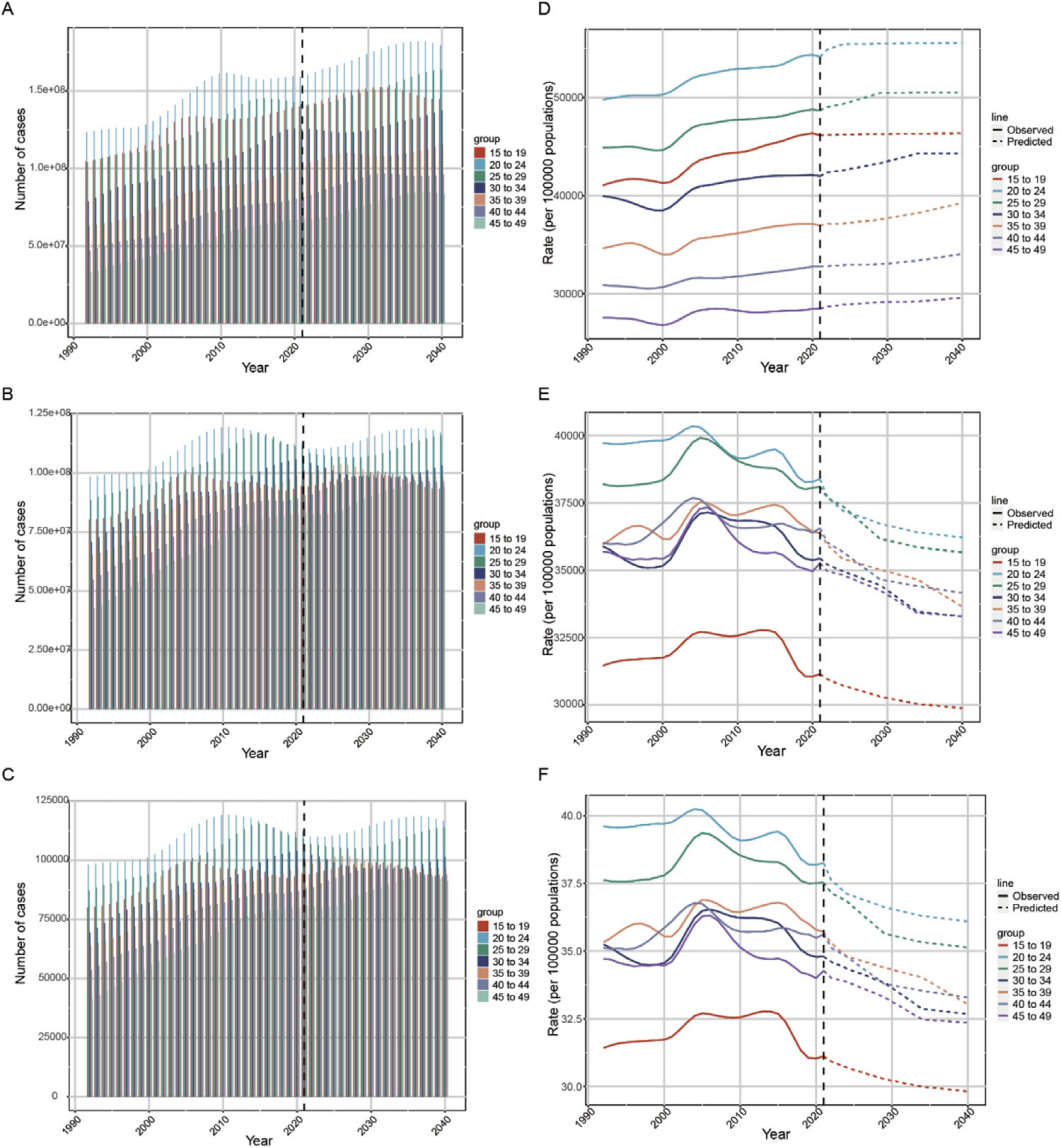
The projected global case numbers and ASR of caries among WCBA to 2040 by age group. (1) The predicted case number of incidence (A), prevalence (B), and DALYs (C); (2) The predicted ASR of incidence (D), prevalence (E), and DALYs (F). ASR: age-standardized rate. DALYs: disability-adjusted life-years. WCBA: women of childbearing age.

According to Nordpred projections, the global number of incident cases, prevalent cases, and DALYs due to caries among WCBA are predicted to increase slightly from 2022 to 2040 by 12.03%, 3.99%, and 3.87%, respectively. In terms of ASR, the ASIR is projected to increase by 2.83%, while the ASPR and ASDR are anticipated to decrease by 4.91% and 5.00%, respectively. Across all age groups, both the number and ASR of incidence are forecast to rise slightly. Although the number of prevalent cases and DALYs are also projected to increase modestly in most age groups, the ASPR and ASDR are predicted to decline across all age groups, with the largest decrease observed in the 35-39 age group (Fig. 3; Supplementary Table 6). Sensitivity analyses based on the lower and upper data bounds produced similar forecasted patterns, validating the reliability of the projections (Supplementary Tables 7-8).

## Discussion

Caries is one of the most widespread diseases globally. The oral health goals of the WHO for 2023-2030 aim to reduce its prevalence through primary prevention and expanded access to essential oral care.^32^ A comprehensive understanding of trends in caries prevalence is critical for evaluating progress toward these goals. However, most previous GBD studies have focused primarily on paediatric populations.^8^^,^^33^ This study addresses a gap by comprehensively assessing the caries burden among WCBA from 1990 to 2021. Based on the GBD 2021, this study provides a solid foundation for improving oral health strategies for WCBA.

In 2021, approximately 37% of the global WCBA population—about 700 million—were affected by caries. Between 1990 and 2021, the ASIR of caries in WCBA increased significantly, while the ASPR and ASDR slightly declined. Though divergent, this pattern—consistent with the GBD definition of untreated dental caries—supports our hypothesis that expanded access to restorative treatment has shortened the average duration of active carious lesions. This is explained by the relationship between prevalence, incidence, and disease duration (Prevalence≈Incidence×Duration). The decrease in disease duration is most likely due to improvements in dental care. Over the past three decades, many regions have expanded access to restorative dental services, broadened dental insurance coverage, and increased public awareness of oral health. These factors enable earlier diagnosis and treatment, shortening the duration of otherwise long-standing carious lesions. As a result, while more WCBA develop caries each year (rising incidence), they experience less time with active disease (falling prevalence), and the associated disability decreases (falling DALYs). Socioeconomic development and targeted public health initiatives have thus played a key role in "compressing" caries morbidity, even if they have not yet reduced its overall occurrence.

Sugar plays a significant role in caries development, with evidence supporting a dose-response relationship.^34^ In recent years, sugar-sweetened beverage (SSB) consumption has risen globally, especially in regions such as North America, Latin America, Australasia, and Western Europe,^35^ closely linking it to the high burden of caries in WCBA in these areas. Due to demographic factors such as population growth and aging, the burden of SSB consumption is highest in densely populated countries like India and China.^36^^,^^37^ In response, many countries have introduced sugar reduction policies (e.g., sugar taxes in Mexico and the United Kingdom, calorie labelling in Chile, and SSB taxes in France and South Africa), which have successfully reduced SSB consumption and may help lower caries rates.^35^ However, industry responses, such as product reformulation or substitution with artificial sweeteners, pose ongoing challenges for comprehensive public health strategies addressing SSB-related oral health issues.

In 2021, WCBA in low-middle SDI regions showed the highest ASIR, while those in low SDI regions had the highest ASPR and ASDR. Limited healthcare resources, high treatment costs, and inadequate insurance coverage contribute to the issue. Socio-culturally, traditional gender roles neglect women's health, and family resources are mainly allocated to men and children. Additionally, the dietary habits of WCBA in lower SDI countries are characterized by a high consumption of sugary foods, which are affordable and readily available due to the presence of processed food products. A systematic review found that adolescent girls in low- and middle-income countries increasingly adopted a "Westernized" diet, consuming more fats, sugars, and salts.^38^ In some low SDI regions, insufficient fluoride in drinking water exacerbates the issue.^39^ However, the observed correlations between SDI and caries burden were weak, suggesting that socioeconomic development accounts for only a small portion of the variation. This is exemplified by the Republic of Korea, a high-SDI country that paradoxically recorded the highest ASIR among WCBA. This paradox can be partly attributed to historical dietary trends, specifically Korea's marked increase in sugar consumption since the late 19th century, with per capita annual sugar intake reaching 23.7 kg in the early 2000s—higher than the global average.^40^ Such prolonged high sugar exposure during childhood and adolescence likely contributed to an elevated caries risk in adulthood. Furthermore, despite its high SDI, significant intra-national socioeconomic inequalities in dental caries persist among Korean adults,^41^ suggesting that the burden may be unevenly distributed, potentially elevating the overall incidence rate. Importantly, as correlation does not imply causation,^22^ the observed associations between SDI and caries-related indicators should not be interpreted as causal evidence. These findings highlight disparities in caries burden among WCBA across SDI quintiles but should be interpreted cautiously.

A higher prevalence of caries has been observed in pregnant women. A study in Thailand found that 74% of pregnant women had caries, and they were 2.9 times more likely to have caries than non-pregnant women.^42^ In Malaysia, 58.9% of pregnant women had caries.^43^ These underscore the need for restorative dentistry for this group. The increased risk of caries during pregnancy is attributed to higher carbohydrate intake, increased oral acidity from vomiting, and reduced or more acidic saliva.^44^ Gastric acid from vomiting erodes dental enamel, causing structural breakdown and surface roughening, which promotes bacterial adhesion and indirectly increases caries development.^45^ Additionally, pregnant women may prioritize prenatal care over dental checkups, delaying oral health maintenance. Smoking and iron-deficiency anaemia further increase caries risk, and in some developed regions, such as Western and Central Europe, smoking prevalence among women exceeds the global average.^11^^,^^46^^,^^47^

The implications of maternal oral health extend to the next generation. On the protective side, higher dairy and calcium intake during pregnancy is linked to a lower risk of caries in children.^48^ Conversely, maternal caries itself poses risks to both pregnancy and the child. Caries are associated with adverse pregnancy outcomes,^5^ which may be linked to systemic inflammation triggered by oral infections.^49^ Dental pain and poor oral health can also impair nutritional intake, negatively affecting both mother and child. Furthermore, vertical transmission of oral microbiota, including cariogenic bacteria, from mother to infant is a well-documented initial risk factor.^49^, ^50^, ^51^ The mother's oral flora is the primary source of the infant's initial colonization, with transmission shaped by maternal bacterial load and mode of delivery. Yet, the formation of a cariogenic biofilm and subsequent caries is multifactorial. The acquired bacteria must successfully colonize the emerging dentition, and their pathogenicity is strongly influenced by diet, saliva, and the broader oral environment.^50^^,^^51^ Thus, the high burden observed in WCBA represents a modifiable risk factor for childhood caries, underscoring the need for maternal–child studies to quantify this link and guide prevention. Within this framework, infant feeding patterns play a critical modulating role. While breastfeeding benefits infant dental health, prolonged or frequent breastfeeding after infancy can increase caries risk, as lactose exposure promotes cariogenic bacteria growth. To mitigate the risk of early caries in children, all-night breastfeeding should be avoided after the child's first milk teeth emerge, and oral care should be introduced gradually alongside weaning.^52^ Therefore, healthcare providers should emphasize regular dental checkups and good oral hygiene for WCBA, particularly during pregnancy.

Globally, the highest burden of caries is borne by WCBA aged 20-24 years. This group is at the peak of their reproductive years. Hormonal fluctuations during menstruation and pregnancy have been demonstrated to increase susceptibility to caries by altering the oral environment.^52^ Additionally, the consumption of SSB is high among this group, and it is well-documented that high sugar intake increases the risk of caries.^53^ Moreover, many in this group are financially dependent, limiting access to timely dental care. Over the past 32 years, the ASIR for WCBA has increased, possibly linked to the rising use of oral contraceptives (OCs). Research has indicated a higher prevalence of caries in women using OCs.^54^ The situation is also concerning in the 15-19 age group, where increased school pressure and rapid physical and mental changes result in the largest increase in the burden of caries. Furthermore, adolescents in this group are also more likely to experience bulimia nervosa. The disorder poses a major risk for erosive tooth wear, as self-induced vomiting exposes teeth to gastric acid. Binge-eating episodes often involve sugary foods and drinks, further compounding the risk of dental caries.^52^ Adolescent pregnancy is more common in low- and middle-income countries due to limited access to contraception and education, with a global total fertility rate of 41.3 births per 1,000 women in this age group as of 2023, particularly in sub-Saharan Africa and Latin America.^55^ Additionally, smoking prevalence among females aged 15-19 remains high, with stagnant or rising rates in countries like France, Italy, and Spain, despite tobacco control measures.^46^^,^^56^, ^57^, ^58^ These factors contribute to the high incidence of caries in this age group, particularly in the European High SDI region. To reduce this burden, governments should implement age-specific interventions targeting shared risk factors such as diet, smoking, and access to reproductive health services.

From 2022 to 2040, caries among WCBA are projected to increase in the case numbers of incidence, prevalence, and DALYs by 12.03%, 3.99%, and 3.87%, respectively, driven primarily by global population growth. According to the United Nations, the number of WCBA globally is projected to increase from 1.9 billion in 2022 to 2.3 billion in 2040.^55^ Meanwhile, global sugar consumption is projected to continue to increase over the next decade. Growing demand for sugar-rich confectionery products and soft drinks in low-income countries, particularly in Asia and Africa, will likely remain the key driver of the increase in the ASIR of caries in WCBA.^59^ However, the ASPR and ASDR are projected to decrease by 4.91% and 5.00%, respectively. These reductions are attributed mainly to improved therapeutic interventions. Governments should consider these trends and associated risk factors when revising health prevention strategies for WCBA.

It is important to note several limitations of the study. First, as this is a secondary analysis of observational data from the GBD study, its inherent limitations apply to our findings.^1^^,^^33^ Notably, causal relationships cannot be inferred from the observed associations, since these may be confounded by unmeasured factors not captured in this dataset. Second, primary epidemiological data are scarce in some low SDI regions, and GBD estimates for these areas largely rely on predictive modeling, which may compromise the accuracy of regional burden estimates. Third, as the GBD database lacks pregnancy-specific data on caries, our analysis considered WCBA as a whole. This gap highlights the urgent need for future epidemiological studies and surveillance systems to generate pregnancy-specific oral health data, which is essential for designing targeted interventions.

## Conclusion

While global trends in the ASPR and ASDR for caries among WCBA show gradual improvement, the ASIR continues to rise, particularly in lower SDI regions. Specifically, the 20-24 age group carried the highest burden of caries in 2021, while the 15-19 age group experienced the most substantial increase from 1990 to 2021. Projections for 2022-2040 indicate a continued rise in the case numbers of incidence, prevalence, and DALYs associated with caries in WCBA. To achieve the WHO's Oral Health 2023-2030 goals, it is imperative to strengthen healthcare policies and allocate more significant resources to prevent and manage caries in this population.

## Conflict of interest

None disclosed.

## Appendix Supplementary materials

Image, application 1

## Footnotes

Supplementary material associated with this article can be found in the online version at doi:10.1016/j.identj.2026.109658.
