# Supplementary material for: Global, Regional, and National Burden of Caries Among Women of Childbearing Age From 1990 to 2021 and Prediction
Source: Int Dent J. 2026 Jun 17;76(4):109658. doi: 10.1016/j.identj.2026.109658 (PMC13311817; doi:10.1016/j.identj.2026.109658)

**Global, regional, and national burden of caries among women of childbearing age from 1990-2021 and prediction**

**Table of contents**

[Supplementary Table 1 1](#_Toc224685379)

[Supplementary Table 2 14](#_Toc224685380)

[Supplementary Table 3 27](#_Toc224685381)

[Supplementary Table 4 40](#_Toc224685382)

[Supplementary Table 5 53](#_Toc224685383)

[Supplementary Table 6 55](#_Toc224685384)

[Supplementary Table 7 58](#_Toc224685385)

[Supplementary Table 8 61](#_Toc224685386)

[Supplementary Figure 1 64](#_Toc224685387)

[Supplementary Figure 2 65](#_Toc224685388)

Supplementary Table 1**.** Sensitivity analysis of Joinpoint models using lower/upper bounds of ASIR, ASPR, and ASDR for caries among WCBA, 1990-2021.

| **Location** | **ASIR** | | | **ASPR** | | | | **ASDR** | | |
| --- | --- | --- | --- | --- | --- | --- | --- | --- | --- | --- |
|  | **Original AAPC (95% CI)** | **Lower Bound AAPC (95%CI)** | **Upper Bound AAPC (95%CI)** | **Original AAPC (95% CI)** | **Lower Bound AAPC (95%CI)** | **Upper Bound AAPC (95%CI)** | **Original AAPC (95% CI)** | | **Lower Bound-AAPC (95%CI)** | **Upper Bound -AAPC (95%CI)** |
| Global | 0.27 (0.26 to 0.28) | 0.3 (0.29 to 0.32) | 0.24 (0.23 to 0.24) | -0.03 (-0.04 to -0.02) | 0.05 (0.03 to 0.06) | -0.07 (-0.09 to -0.06) | -0.03 (-0.04 to -0.02) | | -0.01 (-0.02 to 0) | -0.03 (-0.04 to -0.02) |
| **SDI quintiles** | | | | | | | | | | |
| High SDI | 0.05 (0.04 to 0.05) | 0.05 (0.04 to 0.06) | 0.03 (0.03 to 0.04) | -0.17 (-0.17 to -0.16) | -0.06 (-0.07 to -0.05) | -0.27 (-0.27 to -0.26) | -0.18 (-0.19 to -0.17) | | -0.12 (-0.14 to -0.1) | -0.15 (-0.17 to -0.14) |
| High-middle SDI | 0.35 (0.32 to 0.37) | 0.35 (0.33 to 0.38) | 0.33 (0.31 to 0.36) | -0.04 (-0.05 to -0.03) | 0.16 (0.14 to 0.17) | -0.13 (-0.14 to -0.13) | -0.04 (-0.05 to -0.03) | | 0.01 (0 to 0.04) | -0.03 (-0.05 to -0.02) |
| Middle SDI | 0.49 (0.47 to 0.5) | 0.56 (0.54 to 0.57) | 0.4 (0.39 to 0.42) | 0.02 (0 to 0.03) | 0.13 (0.11 to 0.15) | -0.04 (-0.05 to -0.03) | 0.02 (0-0.02) | | 0.04 (0.03 to 0.06) | 0.02 (0 to 0.04) |
| Low-middle SDI | 0.06 (0.05 to 0.07) | 0.13 (0.12 to 0.14) | 0.02 (0.01 to 0.03) | -0.16 (-0.17 to -0.14) | -0.15 (-0.17 to -0.13) | -0.16 (-0.17 to -0.14) | -0.15 (-0.17 to -0.14) | | -0.16 (-0.19 to -0.14) | -0.16 (-0.19 to -0.14) |
| Low SDI | 0.03 (0.03 to 0.04) | 0.04 (0.03 to 0.05) | 0.01 (0 to 0.02) | -0.15 (-0.16 to -0.14) | -0.17 (-0.19 to -0.15) | -0.1 (-0.12 to -0.1) | -0.14 (-0.15 to -0.13) | | -0.14 (-0.16 to -0.12) | -0.14 (-0.17 to -0.11) |
| **GBD regions** | | | | | | | | | | |
| Andean Latin America | 0.05 (0.03 to 0.07) | 0.07 (0.04 to 0.09) | 0.03 (0 to 0.05) | -0.05 (-0.08 to -0.03) | -0.09 (-0.12 to -0.06) | -0.03 (-0.04 to -0.02) | -0.06 (-0.08 to -0.03) | | -0.06 (-0.1 to -0.02) | -0.07 (-0.09 to -0.05) |
| Australasia | 0.51 (0.49 to 0.53) | 0.52 (0.48 to 0.56) | 0.47 (0.44 to 0.51) | -0.49 (-0.52 to -0.46) | -0.76 (-0.79 to -0.72) | -0.28 (-0.31 to -0.24) | -0.49 (-0.52 to -0.47) | | -0.57 (-0.61 to -0.53) | -0.38 (-0.43 to -0.33) |
| Caribbean | 0.06 (0.05 to 0.07) | 0.2 (0.18 to 0.21) | -0.04 (-0.06 to -0.03) | -0.23 (-0.25 to -0.22) | 0.06 (0.04 to 0.08) | -0.4 (-0.41 to -0.39) | -0.24 (-0.25 to -0.23) | | -0.22 (-0.26 to -0.19) | -0.29 (-0.31 to -0.26) |
| Central Asia | 0.07 (0.06 to 0.08) | 0.11 (0.1 to 0.12) | 0.04 (0.02 to 0.06) | -0.08 (-0.08 to -0.07) | -0.07 (-0.08 to -0.06) | -0.05 (-0.05 to -0.04) | -0.08 (-0.08 to -0.07) | | -0.07 (-0.09 to -0.05) | -0.08 (-0.1 to -0.06) |
| Central Europe | 0.23 (0.21 to 0.24) | 0.26 (0.24 to 0.27) | 0.16 (0.15 to 0.18) | -0.26 (-0.27 to -0.26) | -0.27 (-0.29 to -0.26) | -0.19 (-0.2 to -0.19) | -0.25 (-0.26 to -0.25) | | -0.3 (-0.33 to -0.27) | -0.22 (-0.23 to -0.2) |
| Central Latin America | 0.03 (-0.01 to 0.05) | 0.05 (0.04 to 0.06) | 0.02 (-0.01 to 0.05) | -0.16 (-0.24 to -0.11) | -0.2 (-0.4 to -0.1) | -0.13 (-0.14 to -0.11) | -0.16 (-0.25 to -0.12) | | -0.18 (-0.33 to -0.1) | -0.14 (-0.2 to -0.11) |
| Central Sub-Saharan Africa | -0.03 (-0.05 to -0.02) | -0.05 (-0.08 to -0.03) | -0.03 (-0.06 to 0) | 0.01 (-0.01 to 0.03) | -0.03 (-0.08 to 0) | 0.01 (-0.01 to 0.02) | 0.03 (0.02 to 0.05) | | 0 (-0.03 to 0.03) | 0.08 (0.05 to 0.11) |
| East Asia | 0.9 (0.83 to 0.97) | 0.92 (0.86 to 0.98) | 0.81 (0.74 to 0.88) | 0.11 (0.09 to 0.14) | 0.39 (0.37 to 0.42) | -0.04 (-0.06 to -0.01) | 0.12 (0.09 to 0.14) | | 0.23 (0.2 to 0.26) | 0.09 (0.06 to 0.11) |
| Eastern Europe | 0.03 (0.03 to 0.03) | 0.07 (0.06 to 0.09) | 0 (0 to 0.01) | -0.08 (-0.09 to -0.07) | -0.04 (-0.05 to -0.03) | -0.08 (-0.09 to -0.08) | -0.08 (-0.09 to -0.07) | | -0.1 (-0.11 to -0.08) | -0.07 (-0.09 to -0.06) |
| Eastern Sub-Saharan Africa | 0.06 (0.05 to 0.07) | 0.07 (0.07 to 0.08) | 0.03 (0.01 to 0.05) | -0.22 (-0.23 to -0.21) | -0.31 (-0.32 to -0.3) | -0.14 (-0.15 to -0.14) | -0.2 (-0.21 to -0.2) | | -0.22 (-0.24 to -0.19) | -0.18 (-0.19 to -0.17) |
| High-income Asia Pacific | -0.25 (-0.26 to -0.23) | -0.24 (-0.27 to -0.22) | -0.26 (-0.27 to -0.24) | -0.39 (-0.41 to -0.37) | -0.34 (-0.37 to -0.32) | -0.47 (-0.5 to -0.45) | -0.39 (-0.41 to -0.37) | | -0.33 (-0.36 to -0.3) | -0.4 (-0.43 to -0.37) |
| High-income North America | -0.01 (-0.03 to 0.01) | 0.03 (0.01 to 0.06) | -0.05 (-0.07 to -0.04) | -0.12 (-0.13 to -0.11) | 0.01 (0 to 0.03) | -0.22 (-0.23 to -0.21) | -0.14 (-0.15 to -0.13) | | -0.03 (-0.05 to -0.01) | -0.12 (-0.14 to -0.1) |
| North Africa and Middle East | 0.05 (0.04 to 0.05) | 0.04 (0.03 to 0.05) | 0.04 (0.03 to 0.04) | -0.08 (-0.09 to -0.07) | -0.15 (-0.17 to -0.14) | -0.03 (-0.04 to -0.02) | -0.08 (-0.09 to -0.08) | | -0.1 (-0.12 to -0.07) | -0.09 (-0.1 to -0.08) |
| Oceania | 0.01 (-0.02 to 0.02) | 0.02 (0 to 0.03) | -0.06 (-0.08 to -0.05) | -0.03 (-0.04 to -0.02) | 0.01 (-0.01 to 0.02) | -0.01 (-0.03 to 0.01) | -0.03 (-0.05 to -0.02) | | -0.04 (-0.07 to -0.02) | 0.02 (-0.01 to 0.04) |
| South Asia | 0.09 (0.07 to 0.11) | 0.19 (0.16 to 0.21) | 0.02 (0.01 to 0.03) | -0.22 (-0.25 to -0.19) | -0.17 (-0.21 to -0.14) | -0.21 (-0.23 to -0.18) | -0.21 (-0.24 to -0.18) | | -0.19 (-0.23 to -0.15) | -0.22 (-0.25 to -0.19) |
| Southeast Asia | 0.05 (0.04 to 0.06) | 0.14 (0.13 to 0.16) | 0 (-0.01 to 0.01) | -0.2 (-0.22 to -0.18) | -0.12 (-0.14 to -0.1) | -0.2 (-0.22 to -0.17) | -0.19 (-0.21 to -0.18) | | -0.22 (-0.25 to -0.2) | -0.19 (-0.22 to -0.17) |
| Southern Latin America | 0.03 (0 to 0.07) | 0.31 (0.27 to 0.36) | -0.11 (-0.14 to -0.08) | -0.02 (-0.05 to 0.01) | 0.3 (0.25 to 0.36) | -0.2 (-0.22 to -0.19) | -0.04 (-0.07 to -0.01) | | 0.09 (0.04 to 0.14) | -0.05 (-0.08 to -0.02) |
| Southern Sub-Saharan Africa | 0.04 (0.02 to 0.05) | 0.05 (0.04 to 0.07) | 0.04 (0.02 to 0.06) | -0.22 (-0.24 to -0.2) | -0.28 (-0.3 to -0.26) | -0.15 (-0.17 to -0.13) | -0.25 (-0.27 to -0.23) | | -0.28 (-0.32 to -0.25) | -0.27 (-0.29 to -0.24) |
| Tropical Latin America | 0.08 (0.07 to 0.09) | 0.14 (0.13 to 0.15) | 0.05 (0.04 to 0.05) | -0.04 (-0.06 to -0.02) | 0.04 (0.02 to 0.05) | -0.08 (-0.1 to -0.06) | -0.04 (-0.07 to -0.02) | | -0.03 (-0.05 to 0) | -0.01 (-0.04 to 0.01) |
| Western Europe | 0.15 (0.15 to 0.16) | 0.15 (0.14 to 0.17) | 0.14 (0.13 to 0.14) | -0.23 (-0.23 to -0.22) | -0.07 (-0.09 to -0.06) | -0.37 (-0.38 to -0.35) | -0.23 (-0.24 to -0.22) | | -0.16 (-0.17 to -0.14) | -0.21 (-0.24 to -0.19) |
| Western Sub-Saharan Africa | -0.05 (-0.06 to -0.05) | -0.03 (-0.03 to -0.02) | -0.05 (-0.06 to -0.04) | -0.21 (-0.22 to -0.2) | -0.17 (-0.19 to -0.15) | -0.21 (-0.21 to -0.2) | -0.2 (-0.21 to -0.19) | | -0.2 (-0.24 to -0.17) | -0.18 (-0.2 to -0.16) |
| **Countries** | | | | | | | | | | |
| Afghanistan | 0.06 (0.05 to 0.07) | 0.1 (0.07 to 0.13) | 0.07 (0.04 to 0.1) | -0.04 (-0.05 to -0.03) | -0.08 (-0.1 to -0.05) | -0.04 (-0.05 to -0.03) | -0.04 (-0.05 to -0.03) | | -0.05 (-0.08 to -0.01) | -0.05 (-0.1 to 0.01) |
| Albania | 0.16 (0.15 to 0.18) | 0.3 (0.26 to 0.33) | 0.09 (0.06 to 0.12) | -0.24 (-0.25 to -0.24) | -0.35 (-0.37 to -0.32) | -0.15 (-0.16 to -0.14) | -0.24 (-0.25 to -0.23) | | -0.35 (-0.38 to -0.32) | -0.19 (-0.24 to -0.15) |
| Algeria | 0.09 (0.08 to 0.1) | 0.13 (0.09 to 0.16) | 0.02 (-0.03 to 0.06) | -0.04 (-0.06 to -0.03) | -0.05 (-0.11 to 0) | -0.04 (-0.05 to -0.02) | -0.04 (-0.06 to -0.03) | | -0.11 (-0.13 to -0.08) | -0.06 (-0.1 to -0.02) |
| American Samoa | -0.04 (-0.08 to -0.02) | -0.07 (-0.1 to -0.04) | -0.02 (-0.05 to 0) | 0.07 (0.06 to 0.07) | 0.2 (0.16 to 0.24) | 0.05 (0.03 to 0.06) | 0.05 (0.04 to 0.06) | | 0.1 (0.06 to 0.13) | 0.05 (0.01 to 0.08) |
| Andorra | 0.06 (0.05 to 0.06) | 0.08 (0.02 to 0.13) | 0.06 (0.03 to 0.09) | -0.17 (-0.18 to -0.16) | -0.18 (-0.22 to -0.16) | -0.12 (-0.13 to -0.1) | -0.18 (-0.19 to -0.17) | | -0.16 (-0.2 to -0.12) | -0.16 (-0.19 to -0.13) |
| Angola | 0 (-0.01 to 0.01) | -0.01 (-0.04 to 0.01) | -0.02 (-0.07 to 0.01) | -0.12 (-0.13 to -0.12) | -0.17 (-0.2 to -0.14) | -0.11 (-0.13 to -0.09) | -0.12 (-0.13 to -0.12) | | -0.23 (-0.29 to -0.18) | -0.16 (-0.2 to -0.12) |
| Antigua and Barbuda | 0.11 (0.1 to 0.11) | 0.06 (0.03 to 0.09) | 0.16 (0.12 to 0.19) | -0.12 (-0.13 to -0.12) | -0.19 (-0.24 to -0.15) | -0.09 (-0.11 to -0.08) | -0.12 (-0.13 to -0.11) | | -0.13 (-0.17 to -0.09) | -0.07 (-0.12 to -0.04) |
| Argentina | 0.03 (0 to 0.06) | 0.07 (0.04 to 0.12) | 0 (-0.04 to 0.05) | -0.04 (-0.07 to -0.02) | -0.1 (-0.14 to -0.06) | -0.03 (-0.05 to -0.01) | -0.05 (-0.08 to -0.03) | | -0.07 (-0.1 to -0.04) | 0 (-0.06 to 0.04) |
| Armenia | 0.06 (0.05 to 0.08) | 0.12 (0.08 to 0.15) | 0.12 (0.04 to 0.19) | -0.09 (-0.09 to -0.08) | -0.04 (-0.06 to 0) | -0.05 (-0.06 to -0.03) | -0.09 (-0.1 to -0.08) | | -0.09 (-0.12 to -0.05) | -0.11 (-0.15 to -0.07) |
| Australia | 0.64 (0.61 to 0.66) | 0.66 (0.6 to 0.72) | 0.54 (0.5 to 0.59) | -0.57 (-0.6 to -0.54) | -0.84 (-0.9 to -0.8) | -0.29 (-0.33 to -0.25) | -0.56 (-0.59 to -0.53) | | -0.69 (-0.74 to -0.65) | -0.42 (-0.47 to -0.37) |
| Austria | 0.12 (0.04 to 0.19) | 0.19 (0.09 to 0.31) | 0.1 (0.05 to 0.15) | -0.22 (-0.26 to -0.19) | -0.12 (-0.25 to -0.01) | -0.21 (-0.25 to -0.19) | -0.22 (-0.27 to -0.18) | | -0.21 (-0.32 to -0.1) | -0.17 (-0.22 to -0.13) |
| Azerbaijan | 0.09 (0.07 to 0.1) | 0.12 (0.08 to 0.15) | 0.08 (0.04 to 0.12) | -0.08 (-0.11 to -0.05) | -0.04 (-0.09 to 0) | -0.07 (-0.1 to -0.04) | -0.08 (-0.11 to -0.06) | | -0.09 (-0.13 to -0.04) | -0.06 (-0.1 to -0.03) |
| Bahamas | -0.02 (-0.04 to 0) | 0.05 (0.02 to 0.07) | -0.04 (-0.06 to 0) | -0.08 (-0.09 to -0.07) | -0.05 (-0.08 to -0.01) | -0.05 (-0.08 to -0.03) | -0.08 (-0.09 to -0.07) | | -0.04 (-0.09 to 0) | 0 (-0.03 to 0.03) |
| Bahrain | 0.07 (0.06 to 0.07) | 0.04 (0 to 0.07) | 0.06 (0.03 to 0.09) | -0.13 (-0.14 to -0.12) | -0.15 (-0.18 to -0.13) | -0.09 (-0.11 to -0.07) | -0.14 (-0.15 to -0.14) | | -0.15 (-0.21 to -0.1) | -0.21 (-0.26 to -0.11) |
| Bangladesh | 0.12 (0.1 to 0.13) | 0.17 (0.14 to 0.2) | 0.09 (0.06 to 0.11) | -0.12 (-0.13 to -0.12) | -0.08 (-0.12 to -0.04) | -0.07 (-0.09 to -0.05) | -0.12 (-0.13 to -0.11) | | -0.1 (-0.14 to -0.05) | -0.09 (-0.11 to -0.06) |
| Barbados | 0.09 (0.06 to 0.1) | 0.08 (0.06 to 0.1) | 0.08 (0.04 to 0.11) | -0.02 (-0.03 to -0.01) | -0.03 (-0.06 to -0.01) | -0.01 (-0.03 to 0.01) | -0.02 (-0.03 to -0.02) | | 0.01 (-0.04 to 0.05) | -0.06 (-0.08 to -0.04) |
| Belarus | 0.03 (0.03 to 0.04) | 0.02 (-0.01 to 0.05) | 0.04 (0.01 to 0.1) | -0.07 (-0.09 to -0.05) | -0.11 (-0.16 to -0.07) | -0.02 (-0.03 to 0) | -0.07 (-0.09 to -0.05) | | -0.1 (-0.18 to -0.02) | -0.04 (-0.07 to 0) |
| Belgium | 0.1 (0.07 to 0.14) | 0.01 (-0.07 to 0.09) | 0.1 (0.06 to 0.14) | -0.19 (-0.22 to -0.17) | -0.19 (-0.28 to -0.14) | -0.13 (-0.17 to -0.08) | -0.2 (-0.22 to -0.18) | | -0.14 (-0.21 to -0.1) | -0.17 (-0.23 to -0.13) |
| Belize | 0.13 (0.1 to 0.15) | 0.2 (0.18 to 0.23) | 0.08 (0.03 to 0.12) | -0.62 (-0.65 to -0.58) | -0.68 (-0.74 to -0.59) | -0.42 (-0.48 to -0.35) | -0.62 (-0.63 to -0.6) | | -0.72 (-0.78 to -0.66) | -0.57 (-0.6 to -0.53) |
| Benin | 0.02 (0.01 to 0.03) | 0.07 (0.02 to 0.12) | 0.01 (-0.02 to 0.03) | -0.11 (-0.14 to -0.08) | -0.11 (-0.13 to -0.08) | -0.08 (-0.1 to -0.05) | -0.1 (-0.13 to -0.07) | | -0.1 (-0.15 to -0.04) | -0.05 (-0.08 to -0.02) |
| Bermuda | 0.06 (0.02 to 0.08) | 0.11 (0.04 to 0.15) | 0.08 (0.05 to 0.1) | -0.15 (-0.17 to -0.14) | -0.13 (-0.16 to -0.1) | -0.1 (-0.12 to -0.08) | -0.16 (-0.18 to -0.14) | | -0.23 (-0.29 to -0.18) | -0.13 (-0.17 to -0.09) |
| Bhutan | 0.09 (0.09 to 0.1) | 0.12 (0.05 to 0.19) | 0.1 (0.05 to 0.13) | -0.2 (-0.22 to -0.18) | -0.22 (-0.27 to -0.2) | -0.14 (-0.19 to -0.1) | -0.19 (-0.22 to -0.17) | | -0.23 (-0.27 to -0.2) | -0.22 (-0.31 to -0.15) |
| Bolivia (Plurinational State of) | 0.1 (0.08 to 0.1) | 0.08 (0.06 to 0.09) | 0.09 (0.08 to 0.11) | -0.06 (-0.1 to -0.02) | -0.02 (-0.05 to 0) | -0.05 (-0.08 to -0.02) | -0.04 (-0.07 to -0.01) | | -0.07 (-0.11 to -0.03) | -0.12 (-0.16 to -0.06) |
| Bosnia and Herzegovina | 0.32 (0.31 to 0.33) | 0.35 (0.31 to 0.38) | 0.25 (0.21 to 0.29) | -0.38 (-0.38 to -0.37) | -0.57 (-0.61 to -0.54) | -0.2 (-0.22 to -0.18) | -0.38 (-0.39 to -0.37) | | -0.45 (-0.48 to -0.43) | -0.32 (-0.35 to -0.29) |
| Botswana | -0.03 (-0.05 to -0.02) | 0.06 (0.02 to 0.09) | -0.09 (-0.13 to -0.05) | -0.19 (-0.19 to -0.18) | -0.23 (-0.3 to -0.19) | -0.18 (-0.2 to -0.16) | -0.21 (-0.21 to -0.2) | | -0.17 (-0.2 to -0.13) | -0.26 (-0.3 to -0.22) |
| Brazil | 0.09 (0.08 to 0.1) | 0.15 (0.14 to 0.16) | 0.06 (0.05 to 0.06) | -0.05 (-0.08 to -0.03) | 0.02 (0.01 to 0.04) | -0.09 (-0.11 to -0.07) | -0.06 (-0.08 to -0.03) | | -0.03 (-0.06 to -0.01) | -0.05 (-0.08 to -0.03) |
| Brunei Darussalam | 0.05 (0.03 to 0.07) | 0.02 (-0.05 to 0.08) | 0.08 (0.03 to 0.14) | 0.06 (0.05 to 0.07) | -0.02 (-0.07 to 0.03) | 0.03 (0 to 0.06) | 0.06 (0.05 to 0.07) | | 0.03 (-0.03 to 0.1) | 0.1 (0.06 to 0.13) |
| Bulgaria | 0.21 (0.2 to 0.22) | 0.27 (0.23 to 0.3) | 0.19 (0.16 to 0.21) | -0.16 (-0.16 to -0.15) | -0.22 (-0.25 to -0.19) | -0.09 (-0.11 to -0.08) | -0.15 (-0.16 to -0.14) | | -0.19 (-0.22 to -0.16) | -0.12 (-0.15 to -0.08) |
| Burkina Faso | 0.06 (0.04 to 0.07) | 0.09 (0.06 to 0.12) | 0.03 (-0.01 to 0.09) | -0.12 (-0.15 to -0.09) | -0.11 (-0.17 to -0.02) | -0.08 (-0.1 to -0.06) | -0.1 (-0.13 to -0.07) | | -0.16 (-0.22 to -0.09) | -0.03 (-0.08 to 0.03) |
| Burundi | -0.02 (-0.03 to -0.01) | -0.06 (-0.09 to -0.03) | -0.02 (-0.05 to 0.01) | 0.02 (0.02 to 0.03) | 0.08 (0.02 to 0.12) | 0.01 (0 to 0.02) | 0.03 (0.02 to 0.03) | | 0.08 (0.05 to 0.13) | 0.04 (0 to 0.07) |
| Cabo Verde | 0.07 (0.06 to 0.09) | 0.1 (0.06 to 0.14) | 0.07 (0.02 to 0.11) | -0.19 (-0.21 to -0.18) | -0.26 (-0.29 to -0.24) | -0.14 (-0.16 to -0.13) | -0.19 (-0.21 to -0.18) | | -0.27 (-0.31 to -0.21) | -0.17 (-0.21 to -0.13) |
| Cambodia | 0.18 (0.16 to 0.21) | 0.24 (0.19 to 0.27) | 0.14 (0.11 to 0.17) | -0.16 (-0.17 to -0.16) | -0.16 (-0.19 to -0.14) | -0.11 (-0.13 to -0.1) | -0.14 (-0.15 to -0.14) | | -0.16 (-0.2 to -0.11) | -0.17 (-0.19 to -0.14) |
| Cameroon | 0.05 (0.04 to 0.07) | 0.04 (0.02 to 0.07) | 0.06 (0.03 to 0.08) | -0.01 (-0.04 to 0.02) | 0.02 (-0.02 to 0.06) | 0.03 (0.01 to 0.05) | 0.01 (-0.02 to 0.04) | | 0 (-0.06 to 0.05) | 0.03 (-0.01 to 0.09) |
| Canada | -0.05 (-0.08 to 0) | -0.01 (-0.11 to 0.05) | -0.15 (-0.19 to -0.09) | -0.14 (-0.17 to -0.12) | -0.06 (-0.12 to -0.02) | -0.18 (-0.22 to -0.14) | -0.15 (-0.17 to -0.13) | | -0.05 (-0.11 to 0.02) | -0.06 (-0.12 to 0) |
| Central African Republic | -0.1 (-0.1 to -0.09) | -0.06 (-0.1 to -0.02) | -0.12 (-0.16 to -0.09) | 0.02 (0.01 to 0.02) | -0.04 (-0.07 to -0.02) | 0.01 (-0.01 to 0.03) | 0.03 (0.02 to 0.04) | | 0.01 (-0.09 to 0.11) | 0.03 (-0.01 to 0.06) |
| Chad | 0.09 (0.08 to 0.1) | 0.09 (0.04 to 0.14) | 0.05 (0.02 to 0.07) | -0.09 (-0.1 to -0.08) | -0.08 (-0.11 to -0.05) | -0.08 (-0.11 to -0.05) | -0.08 (-0.09 to -0.07) | | -0.08 (-0.12 to -0.04) | -0.04 (-0.09 to 0) |
| Chile | 0.07 (-0.01 to 0.19) | 0.6 (0.52 to 0.74) | -0.41 (-0.49 to -0.3) | -0.02 (-0.1 to 0.09) | 0.98 (0.85 to 1.17) | -0.66 (-0.7 to -0.6) | -0.03 (-0.09 to 0.03) | | 0.4 (0.32 to 0.5) | -0.12 (-0.19 to -0.04) |
| China | 0.93 (0.86 to 1) | 0.94 (0.88 to 1) | 0.85 (0.77 to 0.92) | 0.12 (0.09 to 0.14) | 0.4 (0.37 to 0.43) | -0.04 (-0.06 to -0.02) | 0.13 (0.1 to 0.15) | | 0.25 (0.22 to 0.28) | 0.09 (0.06 to 0.12) |
| Colombia | 0.16 (0.07 to 0.29) | 0.03 (-0.03 to 0.11) | 0.14 (-0.06 to 0.3) | -0.38 (-0.75 to -0.15) | -0.68 (-1.41 to -0.28) | -0.08 (-0.18 to -0.02) | -0.34 (-0.7 to -0.14) | | -0.44 (-0.97 to -0.15) | -0.24 (-0.56 to -0.06) |
| Comoros | 0.02 (0.01 to 0.03) | 0.06 (0.02 to 0.11) | -0.07 (-0.12 to -0.02) | -0.01 (-0.02 to 0) | -0.06 (-0.11 to -0.02) | 0.04 (0.02 to 0.07) | -0.01 (-0.02 to 0) | | -0.02 (-0.07 to 0.03) | 0.01 (-0.04 to 0.06) |
| Congo | 0.05 (0.04 to 0.05) | 0 (-0.04 to 0.03) | 0.1 (0.07 to 0.12) | -0.05 (-0.05 to -0.04) | -0.08 (-0.11 to -0.06) | -0.07 (-0.09 to -0.04) | -0.03 (-0.04 to -0.03) | | -0.15 (-0.19 to -0.1) | -0.03 (-0.08 to 0.01) |
| Cook Islands | 0.05 (0.04 to 0.06) | 0.15 (0.12 to 0.18) | 0.07 (0.05 to 0.09) | -0.1 (-0.11 to -0.09) | -0.15 (-0.19 to -0.12) | -0.08 (-0.1 to -0.07) | -0.09 (-0.1 to -0.08) | | -0.11 (-0.15 to -0.07) | -0.07 (-0.11 to -0.04) |
| Costa Rica | 0.07 (0.05 to 0.1) | 0.12 (0.1 to 0.14) | 0.06 (0.02 to 0.1) | -0.13 (-0.14 to -0.13) | -0.12 (-0.16 to -0.09) | -0.11 (-0.13 to -0.1) | -0.13 (-0.14 to -0.12) | | -0.13 (-0.18 to -0.09) | -0.16 (-0.21 to -0.12) |
| Coted'Ivoire | -0.02 (-0.03 to -0.01) | -0.01 (-0.03 to 0.02) | -0.01 (-0.06 to 0.04) | -0.04 (-0.05 to -0.03) | -0.01 (-0.04 to 0.02) | -0.04 (-0.06 to -0.02) | -0.02 (-0.02 to -0.01) | | -0.07 (-0.11 to -0.03) | -0.06 (-0.12 to 0) |
| Croatia | 0.23 (0.22 to 0.25) | 0.31 (0.28 to 0.35) | 0.11 (0.07 to 0.15) | -0.24 (-0.25 to -0.23) | -0.31 (-0.34 to -0.29) | -0.15 (-0.17 to -0.13) | -0.24 (-0.25 to -0.23) | | -0.32 (-0.36 to -0.29) | -0.23 (-0.27 to -0.19) |
| Cuba | 0.08 (0.04 to 0.11) | 0.06 (0.02 to 0.09) | 0.02 (-0.04 to 0.07) | -0.1 (-0.14 to -0.07) | -0.1 (-0.17 to -0.06) | -0.06 (-0.09 to -0.04) | -0.1 (-0.13 to -0.08) | | -0.14 (-0.19 to -0.1) | -0.12 (-0.17 to -0.08) |
| Cyprus | 0.1 (0.09 to 0.11) | 0.13 (0.1 to 0.16) | 0.05 (0.02 to 0.09) | -0.19 (-0.21 to -0.18) | -0.3 (-0.34 to -0.27) | -0.14 (-0.16 to -0.12) | -0.19 (-0.2 to -0.18) | | -0.3 (-0.34 to -0.27) | -0.15 (-0.18 to -0.12) |
| Czechia | 0.16 (0.15 to 0.17) | 0.22 (0.19 to 0.25) | 0.14 (0.12 to 0.15) | -0.14 (-0.15 to -0.13) | -0.19 (-0.21 to -0.16) | -0.09 (-0.13 to -0.07) | -0.15 (-0.16 to -0.13) | | -0.18 (-0.21 to -0.15) | -0.14 (-0.18 to -0.11) |
| Democratic People's Republic of Korea | 0.25 (0.18 to 0.32) | 0.21 (0.13 to 0.29) | 0.2 (0.14 to 0.27) | 0.29 (0.25 to 0.32) | 0.29 (0.22 to 0.35) | 0.22 (0.15 to 0.28) | 0.29 (0.25 to 0.33) | | 0.37 (0.29 to 0.44) | 0.28 (0.22 to 0.33) |
| Democratic Republic of the Congo | -0.05 (-0.08 to -0.04) | -0.07 (-0.11 to -0.05) | -0.01 (-0.05 to 0.03) | 0.05 (0.03 to 0.07) | 0.03 (-0.04 to 0.07) | 0.07 (0.04 to 0.1) | 0.08 (0.06 to 0.1) | | 0.11 (0.06 to 0.15) | 0.18 (0.15 to 0.22) |
| Denmark | 0.11 (0.1 to 0.12) | 0.18 (0.13 to 0.22) | 0.1 (0.06 to 0.14) | 0.38 (0.34 to 0.41) | 0.47 (0.3 to 0.63) | 0.33 (0.27 to 0.41) | 0.39 (0.36 to 0.42) | | 0.51 (0.43 to 0.62) | 0.5 (0.47 to 0.55) |
| Djibouti | -0.01 (-0.03 to 0.01) | -0.05 (-0.1 to -0.01) | 0.02 (-0.01 to 0.06) | -0.07 (-0.07 to -0.06) | -0.03 (-0.08 to 0.01) | -0.05 (-0.07 to -0.02) | -0.07 (-0.08 to -0.06) | | -0.04 (-0.12 to 0) | -0.1 (-0.15 to -0.05) |
| Dominica | 0.06 (0.05 to 0.07) | 0.02 (-0.01 to 0.05) | 0.06 (-0.01 to 0.16) | -0.11 (-0.11 to -0.1) | -0.11 (-0.14 to -0.09) | -0.1 (-0.11 to -0.07) | -0.11 (-0.12 to -0.1) | | -0.14 (-0.17 to -0.11) | -0.04 (-0.08 to 0) |
| Dominican Republic | 0.11 (0.1 to 0.12) | 0.13 (0.11 to 0.16) | 0.1 (0.05 to 0.13) | -0.21 (-0.21 to -0.2) | -0.3 (-0.33 to -0.28) | -0.11 (-0.13 to -0.1) | -0.21 (-0.22 to -0.21) | | -0.23 (-0.27 to -0.19) | -0.18 (-0.22 to -0.14) |
| Ecuador | 0.02 (0.01 to 0.03) | -0.01 (-0.05 to 0.02) | 0.02 (0 to 0.04) | -0.06 (-0.08 to -0.04) | -0.08 (-0.12 to -0.04) | -0.04 (-0.07 to -0.02) | -0.06 (-0.08 to -0.04) | | -0.06 (-0.09 to -0.02) | -0.09 (-0.12 to -0.06) |
| Egypt | 0.09 (0.08 to 0.1) | 0.08 (0.05 to 0.11) | 0.06 (0.01 to 0.11) | -0.17 (-0.2 to -0.14) | -0.24 (-0.29 to -0.2) | -0.11 (-0.13 to -0.1) | -0.17 (-0.2 to -0.13) | | -0.19 (-0.23 to -0.13) | -0.14 (-0.17 to -0.1) |
| El Salvador | 0.05 (0.04 to 0.06) | 0.1 (0.04 to 0.14) | 0.05 (0.03 to 0.07) | -0.11 (-0.15 to -0.09) | -0.17 (-0.2 to -0.15) | -0.06 (-0.11 to -0.03) | -0.14 (-0.15 to -0.12) | | -0.18 (-0.22 to -0.14) | -0.1 (-0.15 to -0.05) |
| Equatorial Guinea | 0.09 (0.07 to 0.11) | 0.13 (0.08 to 0.18) | 0.02 (-0.03 to 0.05) | -0.45 (-0.46 to -0.44) | -0.54 (-0.56 to -0.51) | -0.3 (-0.32 to -0.28) | -0.43 (-0.44 to -0.42) | | -0.56 (-0.6 to -0.53) | -0.42 (-0.47 to -0.38) |
| Eritrea | 0.01 (-0.03 to 0.06) | 0.02 (-0.01 to 0.05) | 0.01 (-0.01 to 0.04) | -0.04 (-0.06 to -0.01) | -0.08 (-0.12 to -0.05) | -0.01 (-0.02 to 0) | -0.01 (-0.04 to 0.01) | | -0.05 (-0.09 to -0.01) | 0.02 (-0.01 to 0.05) |
| Estonia | -0.02 (-0.03 to -0.01) | -0.05 (-0.12 to 0.01) | 0.02 (-0.02 to 0.07) | -0.21 (-0.22 to -0.2) | -0.2 (-0.25 to -0.14) | -0.17 (-0.21 to -0.14) | -0.2 (-0.22 to -0.19) | | -0.24 (-0.29 to -0.2) | -0.19 (-0.23 to -0.17) |
| Eswatini | -0.01 (-0.03 to 0.01) | 0.05 (0.02 to 0.08) | 0.08 (0.04 to 0.13) | -0.12 (-0.13 to -0.11) | -0.11 (-0.18 to -0.02) | -0.14 (-0.16 to -0.11) | -0.16 (-0.17 to -0.15) | | -0.11 (-0.18 to -0.01) | -0.2 (-0.24 to -0.16) |
| Ethiopia | 0 (0 to 0.01) | 0.02 (0 to 0.04) | -0.02 (-0.03 to 0) | -0.23 (-0.24 to -0.22) | -0.28 (-0.29 to -0.26) | -0.17 (-0.17 to -0.15) | -0.21 (-0.22 to -0.2) | | -0.17 (-0.2 to -0.14) | -0.2 (-0.23 to -0.18) |
| Fiji | 0.02 (0.01 to 0.03) | 0.04 (0.01 to 0.08) | 0.07 (0.04 to 0.11) | -0.06 (-0.08 to -0.05) | -0.1 (-0.16 to -0.05) | -0.04 (-0.06 to -0.02) | -0.06 (-0.08 to -0.05) | | -0.14 (-0.17 to -0.11) | -0.08 (-0.12 to -0.04) |
| Finland | 0.04 (-0.03 to 0.11) | 0.06 (-0.02 to 0.14) | 0.05 (-0.01 to 0.12) | -0.3 (-0.34 to -0.25) | -0.27 (-0.43 to -0.12) | -0.24 (-0.3 to -0.19) | -0.27 (-0.33 to -0.22) | | -0.29 (-0.41 to -0.14) | -0.22 (-0.28 to -0.14) |
| France | 0.12 (0.11 to 0.13) | 0.09 (0.05 to 0.12) | 0.12 (0.09 to 0.15) | -0.25 (-0.28 to -0.24) | -0.43 (-0.47 to -0.39) | -0.12 (-0.14 to -0.08) | -0.26 (-0.28 to -0.24) | | -0.28 (-0.34 to -0.2) | -0.19 (-0.24 to -0.14) |
| Gabon | 0 (-0.01 to 0) | 0.1 (0.06 to 0.13) | 0.09 (0.04 to 0.13) | -0.04 (-0.05 to -0.03) | -0.05 (-0.1 to -0.01) | -0.07 (-0.09 to -0.05) | -0.03 (-0.04 to -0.02) | | 0.02 (-0.05 to 0.07) | -0.01 (-0.04 to 0.02) |
| Gambia | 0.06 (0.05 to 0.07) | 0.09 (0.03 to 0.17) | 0.06 (0 to 0.1) | 0.02 (0.01 to 0.03) | -0.02 (-0.07 to 0.01) | 0.01 (-0.01 to 0.03) | 0.01 (-0.01 to 0.02) | | 0.04 (0 to 0.07) | -0.06 (-0.1 to -0.01) |
| Georgia | 0.15 (0.13 to 0.18) | 0.2 (0.16 to 0.24) | 0.13 (0.1 to 0.16) | -0.02 (-0.04 to -0.01) | 0.02 (-0.01 to 0.06) | 0 (-0.02 to 0.02) | -0.03 (-0.05 to -0.02) | | -0.02 (-0.06 to 0.02) | -0.06 (-0.08 to -0.03) |
| Germany | 0.22 (0.2 to 0.23) | 0.31 (0.28 to 0.35) | 0.28 (0.25 to 0.32) | -0.37 (-0.39 to -0.36) | -0.36 (-0.45 to -0.25) | -0.4 (-0.46 to -0.34) | -0.37 (-0.39 to -0.35) | | -0.36 (-0.43 to -0.27) | -0.38 (-0.43 to -0.31) |
| Ghana | -0.02 (-0.04 to -0.01) | -0.02 (-0.05 to 0.01) | -0.02 (-0.05 to 0.01) | -0.18 (-0.2 to -0.15) | -0.24 (-0.31 to -0.16) | -0.11 (-0.14 to -0.07) | -0.17 (-0.19 to -0.14) | | -0.12 (-0.22 to -0.03) | -0.16 (-0.19 to -0.12) |
| Greece | 0.32 (0.31 to 0.33) | 0.31 (0.27 to 0.35) | 0.3 (0.27 to 0.33) | -0.38 (-0.39 to -0.37) | -0.63 (-0.67 to -0.6) | -0.18 (-0.21 to -0.15) | -0.38 (-0.39 to -0.37) | | -0.56 (-0.58 to -0.54) | -0.31 (-0.36 to -0.26) |
| Greenland | -0.07 (-0.11 to -0.04) | -0.06 (-0.12 to 0.01) | -0.05 (-0.08 to -0.01) | -0.15 (-0.17 to -0.12) | -0.21 (-0.26 to -0.16) | -0.1 (-0.15 to -0.05) | -0.14 (-0.15 to -0.1) | | -0.23 (-0.32 to -0.14) | -0.07 (-0.1 to -0.03) |
| Grenada | 0.12 (0.12 to 0.13) | 0.15 (0.1 to 0.18) | 0.1 (0.08 to 0.11) | -0.16 (-0.16 to -0.15) | -0.19 (-0.24 to -0.15) | -0.09 (-0.11 to -0.07) | -0.16 (-0.16 to -0.15) | | -0.21 (-0.24 to -0.18) | -0.13 (-0.17 to -0.1) |
| Guam | -0.02 (-0.04 to 0) | 0 (-0.03 to 0.02) | -0.01 (-0.04 to 0.02) | 0 (-0.01 to 0.02) | -0.06 (-0.13 to -0.01) | -0.01 (-0.03 to 0.02) | 0.01 (-0.01 to 0.02) | | 0.02 (-0.03 to 0.07) | 0.02 (-0.01 to 0.06) |
| Guatemala | 0.07 (0.07 to 0.08) | 0.08 (0.05 to 0.11) | 0.09 (0.04 to 0.11) | -0.09 (-0.1 to -0.09) | -0.12 (-0.14 to -0.11) | -0.09 (-0.11 to -0.07) | -0.08 (-0.09 to -0.08) | | -0.16 (-0.19 to -0.11) | -0.04 (-0.07 to -0.01) |
| Guinea | -0.03 (-0.04 to -0.02) | -0.04 (-0.08 to 0) | 0.03 (-0.01 to 0.06) | -0.02 (-0.05 to 0.01) | -0.03 (-0.08 to 0.04) | 0.01 (-0.03 to 0.07) | -0.01 (-0.04 to 0.03) | | -0.07 (-0.11 to -0.02) | -0.01 (-0.05 to 0.03) |
| Guinea-Bissau | 0.02 (0.01 to 0.04) | 0.06 (0.03 to 0.1) | 0 (-0.03 to 0.02) | -0.05 (-0.09 to -0.01) | -0.08 (-0.13 to -0.04) | -0.01 (-0.03 to 0.01) | -0.05 (-0.08 to -0.01) | | -0.1 (-0.13 to -0.07) | -0.02 (-0.06 to 0.03) |
| Guyana | 0.11 (0.09 to 0.12) | 0.1 (0.08 to 0.12) | 0.14 (0.12 to 0.17) | -0.18 (-0.18 to -0.17) | -0.19 (-0.22 to -0.16) | -0.12 (-0.13 to -0.11) | -0.18 (-0.19 to -0.17) | | -0.21 (-0.25 to -0.18) | -0.15 (-0.18 to -0.12) |
| Haiti | 0 (0 to 0.01) | 0.04 (0.01 to 0.07) | 0.01 (-0.02 to 0.04) | -0.46 (-0.48 to -0.44) | -0.5 (-0.57 to -0.41) | -0.37 (-0.42 to -0.31) | -0.46 (-0.48 to -0.45) | | -0.54 (-0.59 to -0.48) | -0.34 (-0.38 to -0.29) |
| Honduras | 0.09 (0.08 to 0.1) | 0.07 (0.03 to 0.1) | 0.06 (0.04 to 0.08) | -0.08 (-0.11 to -0.06) | -0.13 (-0.16 to -0.11) | -0.06 (-0.08 to -0.04) | -0.07 (-0.09 to -0.04) | | -0.18 (-0.22 to -0.13) | -0.14 (-0.18 to -0.09) |
| Hungary | 0.14 (0.11 to 0.17) | 0.16 (0.12 to 0.22) | 0.12 (0.06 to 0.17) | -0.19 (-0.2 to -0.17) | -0.19 (-0.25 to -0.14) | -0.12 (-0.13 to -0.1) | -0.18 (-0.2 to -0.16) | | -0.18 (-0.21 to -0.16) | -0.05 (-0.1 to -0.03) |
| Iceland | 0.16 (0.12 to 0.19) | 0.16 (0.1 to 0.21) | 0.11 (0.07 to 0.15) | -0.29 (-0.31 to -0.28) | -0.4 (-0.45 to -0.34) | -0.19 (-0.22 to -0.16) | -0.29 (-0.31 to -0.28) | | -0.36 (-0.4 to -0.31) | -0.28 (-0.3 to -0.25) |
| India | 0.09 (0.07 to 0.11) | 0.22 (0.2 to 0.25) | 0.02 (0.01 to 0.03) | -0.22 (-0.26 to -0.19) | -0.17 (-0.22 to -0.12) | -0.22 (-0.25 to -0.19) | -0.21 (-0.25 to -0.18) | | -0.19 (-0.23 to -0.15) | -0.22 (-0.26 to -0.18) |
| Indonesia | 0.03 (0.01 to 0.05) | 0.08 (0.05 to 0.11) | 0.01 (-0.01 to 0.02) | -0.13 (-0.17 to -0.1) | -0.09 (-0.11 to -0.06) | -0.11 (-0.14 to -0.08) | -0.12 (-0.15 to -0.08) | | -0.15 (-0.18 to -0.11) | -0.13 (-0.16 to -0.1) |
| Iran (Islamic Republic of) | 0.01 (0 to 0.02) | 0 (-0.02 to 0.02) | 0 (-0.01 to 0.01) | -0.07 (-0.09 to -0.06) | -0.06 (-0.07 to -0.05) | -0.06 (-0.07 to -0.04) | -0.08 (-0.09 to -0.07) | | -0.07 (-0.09 to -0.05) | -0.07 (-0.09 to -0.05) |
| Iraq | 0.09 (0.08 to 0.1) | 0.15 (0.12 to 0.18) | 0.06 (0.03 to 0.09) | -0.13 (-0.14 to -0.12) | -0.15 (-0.19 to -0.12) | -0.12 (-0.13 to -0.11) | -0.14 (-0.15 to -0.13) | | -0.19 (-0.23 to -0.14) | -0.12 (-0.16 to -0.08) |
| Ireland | 0.16 (0.14 to 0.17) | 0.22 (0.18 to 0.26) | 0.05 (0.02 to 0.08) | -0.31 (-0.33 to -0.3) | -0.29 (-0.37 to -0.25) | -0.25 (-0.28 to -0.22) | -0.34 (-0.36 to -0.33) | | -0.32 (-0.37 to -0.26) | -0.36 (-0.4 to -0.32) |
| Israel | 0.18 (0.14 to 0.21) | 0.26 (0.23 to 0.3) | 0.13 (0.12 to 0.14) | -0.16 (-0.2 to -0.13) | -0.11 (-0.14 to -0.08) | -0.12 (-0.14 to -0.09) | -0.16 (-0.2 to -0.13) | | -0.12 (-0.19 to -0.06) | -0.18 (-0.22 to -0.14) |
| Italy | 0.04 (0.02 to 0.06) | 0.08 (0.07 to 0.1) | -0.03 (-0.04 to -0.01) | -0.18 (-0.19 to -0.16) | -0.05 (-0.07 to -0.04) | -0.22 (-0.24 to -0.19) | -0.17 (-0.19 to -0.14) | | -0.11 (-0.13 to -0.09) | -0.2 (-0.22 to -0.18) |
| Jamaica | 0.14 (0.11 to 0.16) | 0.08 (0.07 to 0.1) | 0.11 (0.09 to 0.14) | -0.09 (-0.1 to -0.08) | -0.1 (-0.13 to -0.08) | -0.02 (-0.04 to 0) | -0.09 (-0.11 to -0.08) | | -0.13 (-0.18 to -0.09) | -0.05 (-0.08 to 0) |
| Japan | -0.48 (-0.51 to -0.45) | -0.53 (-0.57 to -0.5) | -0.43 (-0.46 to -0.41) | -0.63 (-0.67 to -0.61) | -0.53 (-0.56 to -0.51) | -0.74 (-0.77 to -0.71) | -0.63 (-0.66 to -0.6) | | -0.58 (-0.62 to -0.55) | -0.67 (-0.7 to -0.64) |
| Jordan | 0.03 (0.01 to 0.03) | 0.1 (0.06 to 0.13) | 0.05 (0.02 to 0.07) | -0.1 (-0.11 to -0.09) | -0.14 (-0.17 to -0.11) | -0.08 (-0.09 to -0.07) | -0.1 (-0.11 to -0.1) | | -0.13 (-0.24 to -0.05) | -0.07 (-0.1 to -0.04) |
| Kazakhstan | 0.11 (0.09 to 0.12) | 0.12 (0.08 to 0.16) | 0.12 (0.09 to 0.14) | -0.09 (-0.1 to -0.08) | -0.16 (-0.2 to -0.12) | -0.07 (-0.08 to -0.05) | -0.09 (-0.1 to -0.08) | | -0.16 (-0.2 to -0.12) | -0.08 (-0.13 to -0.04) |
| Kenya | -0.03 (-0.04 to -0.03) | -0.01 (-0.01 to 0) | -0.06 (-0.06 to -0.05) | -0.09 (-0.1 to -0.08) | -0.07 (-0.09 to -0.06) | -0.1 (-0.11 to -0.1) | -0.09 (-0.1 to -0.09) | | -0.06 (-0.08 to -0.05) | -0.09 (-0.1 to -0.07) |
| Kiribati | 0.06 (0.04 to 0.1) | 0.03 (0.01 to 0.06) | 0.1 (0.08 to 0.12) | 0.04 (0.02 to 0.06) | 0.02 (-0.01 to 0.05) | 0.04 (0.01 to 0.06) | 0.04 (0.03 to 0.06) | | 0.05 (-0.06 to 0.12) | -0.02 (-0.05 to 0.01) |
| Kuwait | 0.03 (0.02 to 0.04) | 0.1 (0.07 to 0.13) | 0 (-0.03 to 0.04) | -0.09 (-0.1 to -0.08) | -0.2 (-0.25 to -0.16) | -0.04 (-0.06 to -0.02) | -0.08 (-0.09 to -0.07) | | -0.17 (-0.23 to -0.12) | -0.06 (-0.1 to -0.02) |
| Kyrgyzstan | 0.05 (0.05 to 0.06) | 0.06 (0.01 to 0.11) | 0.06 (0.04 to 0.08) | 0 (-0.01 to 0) | -0.01 (-0.04 to 0.03) | -0.01 (-0.02 to 0.01) | 0 (-0.01 to 0.01) | | -0.04 (-0.08 to 0) | 0 (-0.04 to 0.03) |
| Lao People's Democratic Republic | 0.08 (0.06 to 0.09) | 0.18 (0.14 to 0.21) | 0.06 (0.04 to 0.09) | -0.28 (-0.29 to -0.28) | -0.34 (-0.38 to -0.31) | -0.19 (-0.21 to -0.16) | -0.27 (-0.28 to -0.26) | | -0.4 (-0.45 to -0.35) | -0.26 (-0.29 to -0.23) |
| Latvia | 0.05 (0.01 to 0.07) | 0.05 (0 to 0.08) | 0.05 (0.01 to 0.09) | -0.13 (-0.14 to -0.12) | -0.21 (-0.23 to -0.19) | -0.04 (-0.07 to -0.01) | -0.13 (-0.14 to -0.11) | | -0.09 (-0.12 to -0.04) | -0.12 (-0.15 to -0.08) |
| Lebanon | 0.05 (0.04 to 0.06) | 0 (-0.03 to 0.03) | 0.04 (0.02 to 0.06) | -0.13 (-0.13 to -0.12) | -0.17 (-0.2 to -0.14) | -0.09 (-0.11 to -0.08) | -0.13 (-0.14 to -0.12) | | -0.19 (-0.23 to -0.16) | -0.14 (-0.16 to -0.12) |
| Lesotho | -0.02 (-0.03 to 0) | -0.07 (-0.13 to -0.01) | -0.02 (-0.09 to 0.03) | -0.14 (-0.15 to -0.13) | -0.24 (-0.28 to -0.21) | -0.11 (-0.13 to -0.09) | -0.17 (-0.19 to -0.16) | | -0.22 (-0.26 to -0.18) | -0.2 (-0.24 to -0.16) |
| Liberia | 0.03 (0.02 to 0.04) | 0.07 (0.02 to 0.11) | -0.02 (-0.06 to 0.01) | 0.03 (0.01 to 0.06) | 0.03 (-0.02 to 0.08) | 0 (-0.02 to 0.02) | 0.05 (0.02 to 0.08) | | 0.15 (0.1 to 0.22) | 0 (-0.04 to 0.03) |
| Libya | 0.06 (0.05 to 0.07) | -0.02 (-0.04 to 0) | 0.1 (0.05 to 0.14) | 0.14 (0.12 to 0.16) | 0.21 (0.17 to 0.25) | 0.07 (0.06 to 0.09) | 0.12 (0.1 to 0.13) | | 0.25 (0.2 to 0.31) | 0.07 (0.01 to 0.16) |
| Lithuania | 0.14 (0.13 to 0.15) | 0.15 (0.13 to 0.18) | 0.13 (0.1 to 0.15) | -0.28 (-0.28 to -0.27) | -0.4 (-0.45 to -0.34) | -0.14 (-0.15 to -0.13) | -0.28 (-0.29 to -0.27) | | -0.31 (-0.35 to -0.26) | -0.24 (-0.26 to -0.21) |
| Luxembourg | 0.16 (0.15 to 0.18) | 0.1 (0.07 to 0.12) | 0.11 (0.08 to 0.13) | -0.18 (-0.19 to -0.17) | -0.13 (-0.17 to -0.08) | -0.14 (-0.16 to -0.12) | -0.18 (-0.19 to -0.17) | | -0.15 (-0.19 to -0.11) | -0.17 (-0.21 to -0.12) |
| Madagascar | 0.26 (0.24 to 0.27) | 0.32 (0.27 to 0.36) | 0.22 (0.18 to 0.25) | -0.17 (-0.18 to -0.16) | -0.27 (-0.3 to -0.24) | -0.12 (-0.14 to -0.11) | -0.17 (-0.17 to -0.16) | | -0.21 (-0.24 to -0.18) | -0.16 (-0.2 to -0.13) |
| Malawi | -0.02 (-0.05 to 0.01) | -0.02 (-0.06 to 0.02) | 0 (-0.03 to 0.03) | -0.07 (-0.08 to -0.05) | -0.08 (-0.12 to -0.04) | -0.04 (-0.05 to -0.02) | -0.04 (-0.05 to -0.03) | | -0.07 (-0.12 to -0.03) | -0.06 (-0.1 to 0) |
| Malaysia | 0.03 (0 to 0.05) | 0.07 (-0.02 to 0.16) | -0.02 (-0.05 to 0.02) | -0.31 (-0.33 to -0.29) | -0.23 (-0.28 to -0.18) | -0.26 (-0.3 to -0.23) | -0.3 (-0.32 to -0.29) | | -0.33 (-0.42 to -0.26) | -0.23 (-0.31 to -0.16) |
| Maldives | 0.03 (0.03 to 0.04) | 0.05 (0.02 to 0.07) | 0.01 (-0.02 to 0.04) | -0.2 (-0.21 to -0.2) | -0.28 (-0.31 to -0.25) | -0.15 (-0.17 to -0.14) | -0.2 (-0.21 to -0.19) | | -0.2 (-0.26 to -0.15) | -0.21 (-0.24 to -0.18) |
| Mali | 0.08 (0.08 to 0.09) | 0.16 (0.13 to 0.2) | 0.1 (0.07 to 0.12) | -0.09 (-0.1 to -0.07) | -0.1 (-0.14 to -0.05) | -0.08 (-0.1 to -0.06) | -0.08 (-0.09 to -0.06) | | -0.11 (-0.16 to -0.07) | -0.05 (-0.08 to 0.01) |
| Malta | 0.09 (0.09 to 0.1) | 0.22 (0.19 to 0.25) | 0.04 (0.01 to 0.07) | -0.26 (-0.27 to -0.25) | -0.33 (-0.36 to -0.31) | -0.2 (-0.22 to -0.17) | -0.27 (-0.28 to -0.26) | | -0.27 (-0.33 to -0.23) | -0.25 (-0.28 to -0.23) |
| Marshall Islands | 0.08 (0.07 to 0.08) | 0.08 (0.04 to 0.11) | 0.15 (0.12 to 0.17) | -0.01 (-0.02 to 0) | -0.05 (-0.09 to 0) | 0 (-0.03 to 0.02) | -0.01 (-0.02 to 0) | | -0.03 (-0.07 to 0.01) | -0.04 (-0.06 to -0.02) |
| Mauritania | 0.03 (0.02 to 0.04) | 0.01 (-0.06 to 0.12) | -0.02 (-0.05 to 0.01) | -0.06 (-0.07 to -0.05) | -0.06 (-0.1 to -0.02) | -0.03 (-0.06 to -0.01) | -0.05 (-0.06 to -0.05) | | -0.05 (-0.09 to -0.02) | -0.01 (-0.05 to 0.02) |
| Mauritius | 0.04 (0.02 to 0.05) | 0.16 (0.13 to 0.21) | -0.01 (-0.05 to 0.03) | -0.21 (-0.21 to -0.2) | -0.28 (-0.32 to -0.24) | -0.12 (-0.14 to -0.11) | -0.21 (-0.22 to -0.2) | | -0.26 (-0.35 to -0.16) | -0.24 (-0.29 to -0.2) |
| Mexico | 0 (-0.01 to 0) | 0.04 (0.03 to 0.04) | 0 (-0.01 to 0) | -0.16 (-0.17 to -0.15) | -0.11 (-0.12 to -0.1) | -0.16 (-0.17 to -0.15) | -0.17 (-0.17 to -0.17) | | -0.12 (-0.12 to -0.11) | -0.18 (-0.2 to -0.16) |
| Micronesia (Federated States of) | -0.03 (-0.04 to -0.02) | 0 (-0.03 to 0.03) | 0.01 (-0.02 to 0.03) | -0.04 (-0.05 to -0.02) | -0.07 (-0.1 to -0.04) | 0 (-0.01 to 0.01) | -0.04 (-0.06 to -0.03) | | -0.05 (-0.07 to -0.02) | 0 (-0.04 to 0.05) |
| Monaco | 0.09 (0.08 to 0.1) | 0.18 (0.14 to 0.22) | 0.02 (-0.01 to 0.06) | -0.23 (-0.24 to -0.22) | -0.25 (-0.28 to -0.22) | -0.19 (-0.22 to -0.16) | -0.24 (-0.25 to -0.23) | | -0.21 (-0.25 to -0.19) | -0.18 (-0.22 to -0.14) |
| Mongolia | 0.12 (0.11 to 0.12) | 0.17 (0.14 to 0.2) | 0.04 (0.01 to 0.07) | -0.19 (-0.2 to -0.17) | -0.17 (-0.2 to -0.15) | -0.13 (-0.15 to -0.11) | -0.18 (-0.2 to -0.16) | | -0.2 (-0.22 to -0.18) | -0.16 (-0.19 to -0.13) |
| Montenegro | 0.29 (0.29 to 0.3) | 0.32 (0.27 to 0.36) | 0.25 (0.22 to 0.27) | -0.22 (-0.23 to -0.21) | -0.23 (-0.29 to -0.19) | -0.12 (-0.13 to -0.1) | -0.22 (-0.23 to -0.21) | | -0.28 (-0.31 to -0.25) | -0.19 (-0.22 to -0.14) |
| Morocco | -0.03 (-0.06 to -0.01) | 0 (-0.04 to 0.05) | -0.04 (-0.11 to 0.01) | -0.23 (-0.29 to -0.18) | -0.25 (-0.33 to -0.2) | -0.22 (-0.28 to -0.16) | -0.24 (-0.29 to -0.19) | | -0.2 (-0.26 to -0.15) | -0.26 (-0.34 to -0.2) |
| Mozambique | 0.69 (0.65 to 0.73) | 0.68 (0.62 to 0.73) | 0.64 (0.61 to 0.68) | -0.83 (-0.86 to -0.8) | -1.35 (-1.4 to -1.32) | -0.48 (-0.5 to -0.45) | -0.83 (-0.87 to -0.78) | | -1.1 (-1.14 to -1.07) | -0.74 (-0.78 to -0.69) |
| Myanmar | 0 (-0.02 to 0.02) | -0.02 (-0.06 to 0.01) | 0.06 (0.01 to 0.11) | -0.29 (-0.3 to -0.28) | -0.27 (-0.34 to -0.22) | -0.23 (-0.26 to -0.2) | -0.29 (-0.3 to -0.27) | | -0.35 (-0.42 to -0.29) | -0.38 (-0.42 to -0.34) |
| Namibia | 0.13 (0.11 to 0.16) | 0.12 (0.09 to 0.16) | 0.15 (0.1 to 0.2) | -0.06 (-0.07 to -0.05) | -0.04 (-0.09 to -0.01) | -0.04 (-0.07 to -0.01) | -0.05 (-0.07 to -0.04) | | -0.04 (-0.09 to 0.01) | -0.08 (-0.11 to -0.04) |
| Nauru | -0.05 (-0.08 to -0.02) | -0.13 (-0.16 to -0.11) | -0.01 (-0.04 to 0.04) | 0.06 (0.04 to 0.08) | 0.02 (-0.03 to 0.06) | 0.07 (0.04 to 0.1) | 0.05 (0.03 to 0.07) | | 0.09 (0.05 to 0.13) | 0.1 (0.05 to 0.13) |
| Nepal | 0.02 (-0.02 to 0.05) | -0.03 (-0.09 to 0.02) | -0.01 (-0.05 to 0.02) | -0.28 (-0.3 to -0.27) | -0.3 (-0.33 to -0.27) | -0.21 (-0.24 to -0.19) | -0.27 (-0.29 to -0.25) | | -0.26 (-0.29 to -0.22) | -0.27 (-0.31 to -0.22) |
| Netherlands | 0.01 (-0.01 to 0.02) | 0.05 (0 to 0.1) | 0.05 (0.01 to 0.09) | -0.28 (-0.3 to -0.25) | -0.27 (-0.31 to -0.21) | -0.25 (-0.29 to -0.22) | -0.29 (-0.31 to -0.26) | | -0.34 (-0.39 to -0.3) | -0.36 (-0.43 to -0.29) |
| New Zealand | 0.04 (0.03 to 0.05) | -0.02 (-0.05 to 0.01) | 0.07 (0.04 to 0.11) | -0.1 (-0.12 to -0.08) | -0.1 (-0.13 to -0.07) | -0.07 (-0.09 to -0.06) | -0.1 (-0.12 to -0.07) | | -0.09 (-0.13 to -0.05) | -0.06 (-0.09 to -0.03) |
| Nicaragua | 0.08 (0.05 to 0.1) | 0.13 (0.1 to 0.16) | 0.02 (-0.01 to 0.05) | -0.06 (-0.07 to -0.05) | -0.07 (-0.1 to -0.05) | -0.05 (-0.06 to -0.03) | -0.06 (-0.07 to -0.05) | | -0.09 (-0.13 to -0.05) | -0.11 (-0.14 to -0.09) |
| Niger | 0.02 (0.01 to 0.03) | -0.02 (-0.06 to 0.02) | 0.05 (0.03 to 0.08) | -0.03 (-0.04 to -0.03) | -0.09 (-0.12 to -0.06) | 0.01 (-0.01 to 0.03) | -0.02 (-0.03 to -0.01) | | -0.06 (-0.14 to 0) | -0.06 (-0.09 to -0.03) |
| Nigeria | -0.15 (-0.16 to -0.15) | -0.15 (-0.16 to -0.14) | -0.16 (-0.16 to -0.16) | -0.3 (-0.31 to -0.29) | -0.31 (-0.32 to -0.29) | -0.3 (-0.32 to -0.29) | -0.3 (-0.31 to -0.29) | | -0.25 (-0.27 to -0.23) | -0.29 (-0.31 to -0.27) |
| Niue | 0.08 (0.06 to 0.09) | 0.13 (0.09 to 0.15) | 0.04 (0.01 to 0.07) | -0.04 (-0.04 to -0.03) | -0.02 (-0.07 to 0) | -0.01 (-0.02 to 0) | -0.04 (-0.05 to -0.04) | | -0.09 (-0.12 to -0.06) | -0.09 (-0.14 to -0.05) |
| North Macedonia | 0.27 (0.25 to 0.3) | 0.29 (0.25 to 0.32) | 0.2 (0.18 to 0.22) | -0.23 (-0.24 to -0.22) | -0.38 (-0.41 to -0.36) | -0.12 (-0.14 to -0.1) | -0.23 (-0.24 to -0.21) | | -0.33 (-0.36 to -0.3) | -0.19 (-0.24 to -0.14) |
| Northern Mariana Islands | -0.04 (-0.05 to -0.04) | -0.05 (-0.09 to -0.03) | 0 (-0.05 to 0.05) | 0.06 (0.05 to 0.08) | 0.12 (0.07 to 0.15) | 0.03 (0.02 to 0.04) | 0.06 (0.05 to 0.07) | | 0.03 (0 to 0.06) | 0.03 (-0.03 to 0.07) |
| Norway | 0.09 (0.08 to 0.09) | 0.13 (0.11 to 0.15) | 0.05 (0.04 to 0.06) | -0.21 (-0.33 to -0.15) | -0.21 (-0.36 to -0.14) | -0.18 (-0.27 to -0.13) | -0.21 (-0.33 to -0.15) | | -0.23 (-0.41 to -0.14) | -0.2 (-0.32 to -0.14) |
| Oman | 0.06 (0.05 to 0.07) | 0.08 (0.06 to 0.1) | 0.07 (0.02 to 0.11) | -0.12 (-0.13 to -0.11) | -0.11 (-0.14 to -0.08) | -0.08 (-0.1 to -0.07) | -0.11 (-0.13 to -0.1) | | -0.08 (-0.13 to -0.03) | -0.12 (-0.15 to -0.08) |
| Pakistan | -0.02 (-0.04 to -0.02) | -0.06 (-0.08 to -0.04) | -0.03 (-0.05 to -0.01) | 0.02 (0 to 0.03) | 0.02 (-0.02 to 0.05) | 0.03 (0.02 to 0.04) | 0.02 (0 to 0.03) | | 0.01 (-0.03 to 0.05) | 0.02 (0 to 0.05) |
| Palau | 0.12 (0.08 to 0.15) | 0.09 (0.05 to 0.12) | 0.07 (0.04 to 0.11) | 0 (-0.03 to 0.03) | 0 (-0.07 to 0.07) | -0.02 (-0.04 to -0.01) | 0 (-0.03 to 0.03) | | -0.05 (-0.11 to -0.01) | 0.04 (0 to 0.08) |
| Palestine | 0.08 (0.05 to 0.11) | 0.14 (0.08 to 0.2) | 0.05 (0.03 to 0.08) | -0.17 (-0.18 to -0.16) | -0.24 (-0.28 to -0.2) | -0.1 (-0.12 to -0.08) | -0.16 (-0.18 to -0.15) | | -0.14 (-0.2 to -0.1) | -0.11 (-0.15 to -0.07) |
| Panama | 0.14 (0.13 to 0.15) | 0.13 (0.11 to 0.15) | 0.15 (0.13 to 0.16) | -0.2 (-0.21 to -0.19) | -0.26 (-0.29 to -0.23) | -0.1 (-0.11 to -0.09) | -0.2 (-0.21 to -0.19) | | -0.26 (-0.28 to -0.22) | -0.18 (-0.2 to -0.14) |
| Papua New Guinea | 0 (-0.02 to 0.02) | 0.04 (0.01 to 0.07) | -0.1 (-0.13 to -0.08) | -0.05 (-0.06 to -0.04) | -0.05 (-0.08 to -0.02) | -0.02 (-0.05 to 0) | -0.05 (-0.06 to -0.04) | | -0.06 (-0.11 to -0.01) | -0.01 (-0.05 to 0.03) |
| Paraguay | -0.06 (-0.08 to -0.04) | -0.07 (-0.13 to -0.03) | -0.02 (-0.05 to 0.02) | 0.06 (0.05 to 0.07) | 0.07 (0.03 to 0.1) | 0.01 (0 to 0.01) | 0.05 (0.03 to 0.06) | | -0.02 (-0.09 to 0.03) | 0.04 (0 to 0.07) |
| Peru | 0.06 (0.01 to 0.09) | 0.05 (0 to 0.09) | 0.04 (0 to 0.08) | -0.05 (-0.07 to -0.04) | -0.12 (-0.16 to -0.09) | 0 (-0.03 to 0.02) | -0.05 (-0.07 to -0.04) | | -0.04 (-0.11 to 0) | -0.03 (-0.06 to 0.01) |
| Philippines | 0.04 (0.03 to 0.04) | 0.11 (0.1 to 0.11) | 0 (-0.01 to 0.01) | -0.08 (-0.09 to -0.08) | -0.01 (-0.02 to 0) | -0.12 (-0.13 to -0.12) | -0.08 (-0.09 to -0.07) | | -0.04 (-0.06 to -0.02) | -0.1 (-0.12 to -0.09) |
| Poland | 0.13 (0.11 to 0.15) | 0.16 (0.14 to 0.17) | 0.1 (0.08 to 0.12) | -0.21 (-0.24 to -0.19) | -0.19 (-0.23 to -0.16) | -0.18 (-0.22 to -0.15) | -0.2 (-0.23 to -0.18) | | -0.24 (-0.27 to -0.22) | -0.18 (-0.2 to -0.16) |
| Portugal | 0.13 (0.11 to 0.15) | 0.12 (0.08 to 0.15) | 0.06 (0.01 to 0.09) | -0.19 (-0.21 to -0.17) | -0.11 (-0.18 to -0.06) | -0.13 (-0.15 to -0.11) | -0.19 (-0.21 to -0.17) | | -0.21 (-0.26 to -0.17) | -0.16 (-0.2 to -0.11) |
| Puerto Rico | 0.06 (0.05 to 0.07) | 0.14 (0.1 to 0.17) | 0.06 (0.05 to 0.09) | -0.12 (-0.14 to -0.1) | -0.14 (-0.18 to -0.09) | -0.06 (-0.08 to -0.03) | -0.12 (-0.14 to -0.11) | | -0.14 (-0.2 to -0.11) | -0.08 (-0.11 to -0.04) |
| Qatar | 0.1 (0.09 to 0.13) | 0.15 (0.13 to 0.17) | 0.07 (0.04 to 0.1) | -0.13 (-0.14 to -0.13) | -0.1 (-0.13 to -0.07) | -0.16 (-0.18 to -0.15) | -0.14 (-0.15 to -0.13) | | -0.16 (-0.18 to -0.13) | -0.13 (-0.16 to -0.1) |
| Republic of Korea | 0.03 (0.02 to 0.03) | 0.09 (0.03 to 0.13) | 0.03 (-0.01 to 0.07) | -0.19 (-0.2 to -0.18) | -0.14 (-0.21 to -0.08) | -0.17 (-0.2 to -0.15) | -0.19 (-0.2 to -0.17) | | -0.19 (-0.29 to -0.14) | -0.16 (-0.25 to -0.11) |
| Republic of Moldova | 0 (-0.01 to 0.01) | -0.04 (-0.06 to -0.02) | 0.02 (-0.01 to 0.05) | -0.04 (-0.07 to -0.02) | -0.1 (-0.14 to -0.05) | 0.01 (0 to 0.03) | -0.05 (-0.07 to -0.03) | | -0.07 (-0.1 to -0.04) | -0.1 (-0.13 to -0.07) |
| Romania | 0.3 (0.29 to 0.32) | 0.35 (0.32 to 0.38) | 0.22 (0.19 to 0.25) | -0.23 (-0.25 to -0.22) | -0.32 (-0.34 to -0.3) | -0.15 (-0.16 to -0.13) | -0.23 (-0.24 to -0.22) | | -0.23 (-0.26 to -0.19) | -0.18 (-0.24 to -0.13) |
| Russian Federation | 0.04 (0.03 to 0.04) | 0.09 (0.08 to 0.1) | -0.01 (-0.01 to 0) | -0.1 (-0.12 to -0.09) | -0.04 (-0.06 to -0.03) | -0.14 (-0.15 to -0.12) | -0.11 (-0.13 to -0.09) | | -0.13 (-0.15 to -0.11) | -0.12 (-0.14 to -0.11) |
| Rwanda | 0 (-0.01 to 0.01) | 0.05 (0.01 to 0.08) | 0.01 (-0.03 to 0.04) | -0.15 (-0.16 to -0.14) | -0.16 (-0.19 to -0.14) | -0.08 (-0.11 to -0.05) | -0.15 (-0.16 to -0.15) | | -0.14 (-0.19 to -0.11) | -0.17 (-0.19 to -0.15) |
| Saint Kitts and Nevis | 0.12 (0.11 to 0.13) | 0.11 (0.08 to 0.13) | 0.08 (0.06 to 0.11) | -0.15 (-0.16 to -0.15) | -0.21 (-0.24 to -0.18) | -0.11 (-0.13 to -0.1) | -0.15 (-0.15 to -0.15) | | -0.2 (-0.23 to -0.16) | -0.18 (-0.22 to -0.15) |
| Saint Lucia | 0.08 (0.06 to 0.09) | 0.1 (0.05 to 0.15) | 0.04 (0.02 to 0.05) | -0.14 (-0.15 to -0.11) | -0.21 (-0.25 to -0.19) | -0.09 (-0.13 to -0.06) | -0.13 (-0.14 to -0.11) | | -0.16 (-0.21 to -0.12) | -0.14 (-0.16 to -0.11) |
| Saint Vincent and the Grenadines | 0.3 (0.28 to 0.31) | 0.42 (0.38 to 0.46) | 0.21 (0.19 to 0.24) | -0.31 (-0.33 to -0.3) | -0.37 (-0.4 to -0.33) | -0.2 (-0.22 to -0.18) | -0.31 (-0.33 to -0.29) | | -0.33 (-0.36 to -0.3) | -0.26 (-0.31 to -0.22) |
| Samoa | 0.01 (0.01 to 0.02) | 0.01 (-0.05 to 0.04) | 0.09 (0.06 to 0.12) | -0.04 (-0.05 to -0.04) | -0.06 (-0.08 to -0.03) | -0.04 (-0.06 to -0.02) | -0.05 (-0.05 to -0.04) | | -0.1 (-0.13 to -0.07) | -0.07 (-0.09 to -0.04) |
| San Marino | 0.13 (0.12 to 0.14) | 0.15 (0.1 to 0.18) | 0.14 (0.08 to 0.19) | -0.12 (-0.14 to -0.11) | -0.13 (-0.16 to -0.11) | -0.1 (-0.12 to -0.07) | -0.13 (-0.15 to -0.12) | | -0.09 (-0.12 to -0.05) | -0.05 (-0.1 to -0.01) |
| Sao Tome and Principe | 0.09 (0.07 to 0.1) | 0.08 (0.03 to 0.11) | 0.08 (0.04 to 0.11) | -0.03 (-0.03 to -0.02) | -0.01 (-0.05 to 0.03) | -0.02 (-0.05 to -0.01) | -0.03 (-0.04 to -0.02) | | 0.03 (-0.03 to 0.08) | 0.03 (-0.01 to 0.06) |
| Saudi Arabia | 0.1 (0.09 to 0.1) | 0.1 (0.07 to 0.13) | 0.06 (0.04 to 0.07) | -0.13 (-0.18 to -0.11) | -0.11 (-0.14 to -0.08) | -0.12 (-0.18 to -0.09) | -0.14 (-0.19 to -0.11) | | -0.16 (-0.21 to -0.09) | -0.17 (-0.23 to -0.13) |
| Senegal | 0.04 (0.03 to 0.05) | 0.03 (-0.03 to 0.07) | 0.02 (-0.01 to 0.05) | -0.02 (-0.02 to -0.01) | -0.08 (-0.13 to -0.05) | 0.02 (0 to 0.04) | 0 (-0.01 to 0) | | -0.06 (-0.1 to -0.02) | -0.01 (-0.04 to 0.01) |
| Serbia | 0.26 (0.18 to 0.32) | 0.23 (0.15 to 0.29) | 0.23 (0.15 to 0.3) | -0.25 (-0.36 to -0.13) | -0.41 (-0.6 to -0.28) | -0.11 (-0.17 to -0.06) | -0.27 (-0.34 to -0.21) | | -0.32 (-0.41 to -0.24) | -0.21 (-0.29 to -0.14) |
| Seychelles | 0.01 (0 to 0.02) | 0.08 (0.05 to 0.11) | 0.01 (-0.02 to 0.05) | -0.16 (-0.17 to -0.16) | -0.07 (-0.1 to -0.06) | -0.09 (-0.11 to -0.07) | -0.17 (-0.18 to -0.16) | | -0.08 (-0.17 to -0.02) | -0.18 (-0.22 to -0.14) |
| Sierra Leone | 0.35 (0.34 to 0.37) | 0.49 (0.45 to 0.52) | 0.29 (0.25 to 0.33) | -0.41 (-0.43 to -0.39) | -0.5 (-0.55 to -0.46) | -0.32 (-0.35 to -0.3) | -0.41 (-0.43 to -0.38) | | -0.49 (-0.53 to -0.44) | -0.36 (-0.39 to -0.33) |
| Singapore | 0 (-0.02 to 0.02) | 0.01 (-0.06 to 0.08) | 0.01 (-0.05 to 0.06) | -0.13 (-0.15 to -0.11) | -0.13 (-0.18 to -0.06) | -0.18 (-0.21 to -0.15) | -0.12 (-0.14 to -0.1) | | -0.08 (-0.15 to -0.02) | -0.1 (-0.14 to -0.06) |
| Slovakia | 0.11 (0.1 to 0.13) | 0.24 (0.2 to 0.26) | 0.05 (0.02 to 0.07) | -0.23 (-0.24 to -0.22) | -0.32 (-0.34 to -0.3) | -0.16 (-0.17 to -0.14) | -0.23 (-0.24 to -0.21) | | -0.3 (-0.34 to -0.26) | -0.18 (-0.21 to -0.15) |
| Slovenia | 0.13 (0.12 to 0.15) | 0.11 (0.07 to 0.16) | 0.17 (0.12 to 0.2) | -0.17 (-0.18 to -0.17) | -0.42 (-0.44 to -0.39) | -0.05 (-0.08 to -0.03) | -0.17 (-0.18 to -0.16) | | -0.35 (-0.39 to -0.33) | -0.14 (-0.18 to -0.11) |
| Solomon Islands | -0.01 (-0.03 to 0) | 0.03 (0 to 0.05) | 0.04 (0.01 to 0.07) | -0.02 (-0.05 to 0) | 0 (-0.04 to 0.03) | 0.01 (-0.01 to 0.04) | -0.02 (-0.05 to 0) | | -0.11 (-0.19 to -0.03) | -0.02 (-0.05 to 0.01) |
| Somalia | -0.05 (-0.08 to -0.02) | -0.05 (-0.08 to -0.02) | -0.05 (-0.07 to -0.02) | 0.01 (0.01 to 0.02) | 0.01 (-0.02 to 0.04) | -0.01 (-0.03 to 0) | 0.03 (0.02 to 0.03) | | 0.02 (-0.03 to 0.07) | 0.04 (0.01 to 0.07) |
| South Africa | 0.08 (0.07 to 0.09) | 0.12 (0.1 to 0.13) | 0.07 (0.06 to 0.08) | -0.3 (-0.31 to -0.28) | -0.35 (-0.36 to -0.33) | -0.21 (-0.23 to -0.19) | -0.31 (-0.33 to -0.29) | | -0.36 (-0.41 to -0.31) | -0.31 (-0.34 to -0.28) |
| South Sudan | -0.06 (-0.06 to -0.05) | -0.13 (-0.14 to -0.11) | -0.04 (-0.06 to -0.02) | 0.13 (0.12 to 0.14) | 0.18 (0.14 to 0.21) | 0.11 (0.09 to 0.14) | 0.14 (0.13 to 0.15) | | 0.14 (0.11 to 0.17) | 0.08 (0.05 to 0.11) |
| Spain | 0.26 (0.25 to 0.28) | 0.33 (0.3 to 0.37) | 0.22 (0.18 to 0.25) | -0.27 (-0.29 to -0.26) | -0.31 (-0.35 to -0.27) | -0.17 (-0.2 to -0.14) | -0.28 (-0.29 to -0.26) | | -0.24 (-0.27 to -0.19) | -0.25 (-0.31 to -0.21) |
| Sri Lanka | 0.04 (0.02 to 0.04) | 0.1 (0.06 to 0.14) | 0.01 (-0.02 to 0.04) | -0.26 (-0.26 to -0.25) | -0.32 (-0.36 to -0.29) | -0.2 (-0.22 to -0.18) | -0.25 (-0.26 to -0.24) | | -0.34 (-0.38 to -0.29) | -0.27 (-0.32 to -0.22) |
| Sudan | 0.09 (0.07 to 0.11) | 0.09 (0.06 to 0.11) | 0.05 (0.01 to 0.09) | -0.12 (-0.13 to -0.11) | -0.19 (-0.23 to -0.13) | -0.03 (-0.04 to -0.02) | -0.12 (-0.12 to -0.11) | | -0.15 (-0.2 to -0.1) | -0.09 (-0.12 to -0.07) |
| Suriname | 0.01 (-0.03 to 0.04) | -0.01 (-0.06 to 0.02) | 0.04 (-0.01 to 0.1) | -0.13 (-0.16 to -0.11) | -0.16 (-0.2 to -0.11) | -0.15 (-0.17 to -0.12) | -0.14 (-0.16 to -0.11) | | -0.16 (-0.22 to -0.11) | -0.12 (-0.15 to -0.08) |
| Sweden | 0.4 (0.39 to 0.41) | 0.46 (0.43 to 0.48) | 0.26 (0.23 to 0.28) | 0.17 (0.15 to 0.19) | 0.35 (0.32 to 0.38) | 0.02 (0 to 0.04) | 0.16 (0.15 to 0.18) | | 0.26 (0.23 to 0.29) | 0.03 (-0.02 to 0.06) |
| Switzerland | 0.16 (0.16 to 0.17) | 0.16 (0.14 to 0.2) | 0.14 (0.13 to 0.16) | -0.12 (-0.12 to -0.11) | -0.13 (-0.16 to -0.1) | -0.06 (-0.08 to -0.04) | -0.12 (-0.13 to -0.11) | | -0.15 (-0.18 to -0.13) | -0.15 (-0.2 to -0.11) |
| Syrian Arab Republic | 0.04 (0.03 to 0.05) | 0.04 (0.01 to 0.07) | 0.03 (0.01 to 0.04) | -0.09 (-0.1 to -0.08) | -0.16 (-0.21 to -0.13) | -0.01 (-0.03 to 0.01) | -0.09 (-0.1 to -0.08) | | -0.07 (-0.11 to -0.03) | -0.13 (-0.17 to -0.08) |
| Taiwan (Province of China) | -0.07 (-0.14 to -0.01) | -0.09 (-0.18 to 0.01) | -0.1 (-0.2 to 0) | -0.17 (-0.21 to -0.13) | -0.2 (-0.26 to -0.15) | -0.15 (-0.19 to -0.09) | -0.17 (-0.21 to -0.12) | | -0.17 (-0.22 to -0.12) | -0.17 (-0.23 to -0.12) |
| Tajikistan | -0.03 (-0.05 to -0.01) | -0.07 (-0.1 to -0.03) | -0.09 (-0.14 to -0.02) | -0.04 (-0.04 to -0.04) | 0.01 (-0.03 to 0.07) | -0.04 (-0.07 to -0.01) | -0.04 (-0.05 to -0.04) | | -0.06 (-0.11 to -0.02) | -0.09 (-0.13 to -0.03) |
| Thailand | 0.15 (0.14 to 0.16) | 0.22 (0.2 to 0.24) | 0.17 (0.11 to 0.2) | -0.18 (-0.19 to -0.17) | -0.13 (-0.17 to -0.08) | -0.16 (-0.18 to -0.15) | -0.17 (-0.18 to -0.16) | | -0.17 (-0.25 to -0.11) | -0.17 (-0.21 to -0.13) |
| Timor-Leste | 0.11 (0.1 to 0.13) | 0.14 (0.11 to 0.18) | 0.1 (0.08 to 0.12) | -0.21 (-0.27 to -0.17) | -0.26 (-0.33 to -0.21) | -0.14 (-0.19 to -0.11) | -0.19 (-0.25 to -0.15) | | -0.17 (-0.23 to -0.12) | -0.16 (-0.21 to -0.11) |
| Togo | -0.04 (-0.05 to -0.03) | 0 (-0.03 to 0.02) | -0.04 (-0.08 to 0) | -0.59 (-0.61 to -0.56) | -0.51 (-0.58 to -0.41) | -0.53 (-0.6 to -0.44) | -0.58 (-0.61 to -0.56) | | -0.56 (-0.61 to -0.51) | -0.47 (-0.54 to -0.34) |
| Tokelau | 0.06 (0.05 to 0.08) | 0.04 (0 to 0.07) | 0.07 (0.05 to 0.09) | -0.11 (-0.12 to -0.1) | -0.17 (-0.19 to -0.16) | -0.06 (-0.08 to -0.04) | -0.1 (-0.11 to -0.09) | | -0.2 (-0.25 to -0.16) | -0.04 (-0.1 to 0.02) |
| Tonga | 0.03 (0.02 to 0.05) | 0.1 (0.03 to 0.16) | 0.09 (0.06 to 0.12) | -0.05 (-0.06 to -0.03) | -0.03 (-0.09 to 0.04) | -0.05 (-0.06 to -0.03) | -0.04 (-0.06 to -0.03) | | -0.1 (-0.14 to -0.06) | -0.03 (-0.06 to 0.01) |
| Trinidad and Tobago | 0.14 (0.13 to 0.14) | 0.28 (0.2 to 0.39) | 0 (-0.06 to 0.07) | -0.8 (-0.82 to -0.78) | -0.83 (-0.94 to -0.72) | -0.54 (-0.61 to -0.48) | -0.8 (-0.82 to -0.78) | | -0.92 (-0.98 to -0.85) | -0.68 (-0.72 to -0.64) |
| Tunisia | 0.1 (0.08 to 0.12) | 0.16 (0.13 to 0.19) | 0.07 (0.05 to 0.09) | -0.12 (-0.13 to -0.11) | -0.1 (-0.15 to -0.06) | -0.1 (-0.11 to -0.09) | -0.13 (-0.14 to -0.12) | | -0.26 (-0.32 to -0.21) | -0.11 (-0.14 to -0.07) |
| Turkey | 0.07 (0.06 to 0.08) | 0.08 (0.06 to 0.11) | 0.03 (0 to 0.06) | -0.19 (-0.21 to -0.16) | -0.26 (-0.32 to -0.2) | -0.12 (-0.13 to -0.1) | -0.19 (-0.21 to -0.16) | | -0.24 (-0.29 to -0.19) | -0.2 (-0.23 to -0.16) |
| Turkmenistan | 0.19 (0.17 to 0.21) | 0.23 (0.2 to 0.27) | 0.13 (0.1 to 0.16) | -0.11 (-0.13 to -0.1) | -0.13 (-0.15 to -0.1) | -0.08 (-0.1 to -0.07) | -0.11 (-0.12 to -0.1) | | -0.14 (-0.16 to -0.11) | -0.1 (-0.13 to -0.07) |
| Tuvalu | -0.03 (-0.06 to 0) | -0.03 (-0.06 to -0.01) | -0.01 (-0.04 to 0.02) | -0.1 (-0.13 to -0.06) | -0.2 (-0.26 to -0.16) | -0.02 (-0.06 to 0.01) | -0.08 (-0.11 to -0.06) | | -0.14 (-0.19 to -0.06) | -0.05 (-0.09 to -0.01) |
| Uganda | -0.03 (-0.11 to 0.03) | -0.1 (-0.17 to -0.06) | 0.02 (-0.03 to 0.09) | -0.14 (-0.16 to -0.1) | -0.26 (-0.3 to -0.22) | -0.1 (-0.14 to -0.06) | -0.1 (-0.14 to -0.06) | | -0.18 (-0.26 to -0.13) | -0.13 (-0.16 to -0.1) |
| Ukraine | 0 (-0.02 to 0.03) | -0.03 (-0.08 to 0.02) | 0.01 (-0.02 to 0.04) | 0.01 (0 to 0.02) | -0.06 (-0.09 to -0.01) | 0.01 (0 to 0.03) | 0.01 (0 to 0.02) | | 0.03 (-0.02 to 0.07) | -0.01 (-0.04 to 0.03) |
| United Arab Emirates | -0.02 (-0.02 to -0.01) | -0.05 (-0.09 to -0.01) | 0.01 (-0.02 to 0.04) | -0.03 (-0.06 to 0) | 0.02 (-0.02 to 0.06) | 0.01 (-0.02 to 0.04) | -0.02 (-0.06 to 0.02) | | 0 (-0.08 to 0.05) | 0 (-0.05 to 0.04) |
| United Kingdom | 0.05 (0.02 to 0.06) | 0.11 (0.09 to 0.13) | -0.01 (-0.03 to 0) | -0.24 (-0.24 to -0.23) | -0.17 (-0.19 to -0.16) | -0.27 (-0.28 to -0.26) | -0.24 (-0.24 to -0.23) | | -0.2 (-0.22 to -0.18) | -0.24 (-0.25 to -0.23) |
| United Republic of Tanzania | 0.1 (0.08 to 0.11) | 0.12 (0.08 to 0.15) | 0.08 (0.02 to 0.12) | -0.09 (-0.1 to -0.08) | -0.27 (-0.33 to -0.22) | 0 (-0.03 to 0.03) | -0.05 (-0.07 to -0.04) | | -0.08 (-0.12 to -0.04) | 0.01 (-0.06 to 0.07) |
| United States of America | 0 (-0.02 to 0.01) | 0.13 (0.09 to 0.17) | 0.05 (0 to 0.09) | -0.12 (-0.13 to -0.11) | -0.19 (-0.23 to -0.16) | -0.13 (-0.15 to -0.11) | -0.16 (-0.17 to -0.15) | | -0.02 (-0.04 to 0) | -0.13 (-0.15 to -0.1) |
| United States Virgin Islands | 0.08 (0.06 to 0.09) | 0.03 (0.01 to 0.05) | -0.03 (-0.04 to -0.01) | -0.15 (-0.16 to -0.14) | 0.03 (0.01 to 0.04) | -0.24 (-0.25 to -0.22) | -0.14 (-0.15 to -0.13) | | -0.26 (-0.32 to -0.21) | -0.17 (-0.21 to -0.13) |
| Uruguay | 0.16 (0.14 to 0.17) | 0.14 (0.1 to 0.18) | 0.16 (0.15 to 0.18) | -0.1 (-0.11 to -0.1) | -0.09 (-0.13 to -0.06) | -0.08 (-0.1 to -0.05) | -0.11 (-0.12 to -0.11) | | -0.05 (-0.1 to 0) | -0.17 (-0.21 to -0.13) |
| Uzbekistan | 0.07 (0.06 to 0.08) | 0.1 (0.08 to 0.14) | 0.06 (0.04 to 0.09) | -0.13 (-0.14 to -0.13) | -0.13 (-0.16 to -0.11) | -0.07 (-0.09 to -0.05) | -0.13 (-0.14 to -0.12) | | -0.11 (-0.16 to -0.05) | -0.1 (-0.15 to -0.05) |
| Vanuatu | -0.01 (-0.01 to 0) | -0.03 (-0.06 to 0) | 0.01 (-0.02 to 0.03) | 0.01 (0.01 to 0.02) | -0.04 (-0.08 to 0) | 0.01 (-0.01 to 0.03) | 0.01 (0 to 0.01) | | 0 (-0.05 to 0.04) | 0.01 (-0.02 to 0.05) |
| Venezuela (Bolivarian Republic of) | 0.02 (0.01 to 0.02) | 0 (-0.03 to 0.02) | 0.01 (-0.01 to 0.03) | -0.02 (-0.05 to 0) | 0.03 (-0.01 to 0.06) | -0.03 (-0.05 to -0.01) | -0.02 (-0.04 to 0) | | -0.07 (-0.12 to -0.02) | 0.04 (-0.01 to 0.09) |
| Viet Nam | 0.06 (0.05 to 0.07) | 0.18 (0.11 to 0.25) | 0.02 (-0.02 to 0.07) | -0.4 (-0.45 to -0.35) | -0.42 (-0.5 to -0.36) | -0.31 (-0.38 to -0.25) | -0.39 (-0.43 to -0.36) | | -0.41 (-0.46 to -0.37) | -0.37 (-0.41 to -0.34) |
| Yemen | 0.1 (0.08 to 0.12) | 0.1 (0.05 to 0.15) | 0.01 (-0.04 to 0.07) | -0.04 (-0.05 to -0.03) | -0.06 (-0.09 to -0.02) | -0.02 (-0.04 to 0) | -0.04 (-0.05 to -0.03) | | 0.04 (-0.02 to 0.08) | -0.02 (-0.05 to 0.01) |
| Zambia | 0.12 (0.11 to 0.14) | 0.12 (0.08 to 0.14) | 0.1 (0.06 to 0.13) | -0.36 (-0.37 to -0.35) | -0.49 (-0.54 to -0.43) | -0.24 (-0.27 to -0.22) | -0.36 (-0.37 to -0.34) | | -0.4 (-0.45 to -0.35) | -0.24 (-0.3 to -0.19) |
| Zimbabwe | -0.1 (-0.16 to -0.07) | -0.13 (-0.22 to -0.07) | 0 (-0.05 to 0.03) | -0.01 (-0.05 to 0.02) | -0.12 (-0.2 to -0.05) | 0.05 (0.02 to 0.07) | -0.01 (-0.05 to 0.02) | | -0.08 (-0.17 to -0.01) | 0.13 (0.08 to 0.17) |

AAPC: average annual percent change, ASIR: age-standardized incidence rate, ASPR: age-standardized prevalence rate, ASDR: age-standardized DALYs rate, DALYs: disability-adjusted life-years, GBD: Global Burden of Disease, SDI: socio-demographic index, WCBA: women of childbearing age.

Supplementary Table 2**.** The case number and ASR of incidence of caries among WCBA in 1990 and 2021 at 204 countries and territories, with AAPC from 1990 to 2021

| **Location** | **1990** | | **2021** | | **AAPC (95%CI)**  **1990–2021** | ***P*** |
| --- | --- | --- | --- | --- | --- | --- |
|  | **Number(95%UI)** | **ASR (95%UI)** | **Number (95%UI)** | **ASR (95%UI)** |  |  |
| Afghanistan | 843966  (700553-1026252) | 38320.5  (25979.7-52079.67) | 2885744  (2368757-3493296) | 39152.71  (26829.07-52598.89) | 0.06 (0.05 to 0.07) | <0.001 |
| Albania | 305730  (243414-373525) | 35912.28  (23990.6-49551.9) | 228551  (184533-274034) | 37617.77  (25575.64-50860.94) | 0.16 (0.15 to 0.18) | <0.001 |
| Algeria | 2368372  (1957664-2887098) | 39516.81  (27076.65-53323.03) | 4534778  (3757255-5384918) | 40689.64  (28141.25-53675.79) | 0.09 (0.08 to 0.1) | <0.001 |
| American Samoa | 4621  (3835-5707) | 37036.45  (25745.23-49714.64) | 4314  (3534-5172) | 37129.87  (25542.23-49882.16) | -0.04 (-0.08 to -0.02) | 0.008 |
| Andorra | 6035  (5017-7091) | 40770.49  (29115.41-52797.29) | 7597  (6362-9159) | 41491.63  (30102.4-53535.63) | 0.06 (0.05 to 0.06) | <0.001 |
| Angola | 981769  (819746-1174718) | 40909.76  (29180.86-53807.07) | 3263599  (2731194-3823052) | 40933.69  (29433.06-53597.84) | 0 (-0.01 to 0.01) | 0.869 |
| Antigua and Barbuda | 6671  (5553-8031) | 40418.13  (28349.46-53287.04) | 9921  (8276-12165) | 41816.81  (29345.22-55745.31) | 0.11 (0.1 to 0.11) | <0.001 |
| Argentina | 3176861  (2556931-3760302) | 39430.49  (26727.73-52700.14) | 4726991  (3818792-5768121) | 40239.8  (27792.99-53876.64) | 0.03 (0 to 0.06) | 0.064 |
| Armenia | 349982  (279626-436032) | 39795.44  (27436.05-53240.35) | 288930  (238981-348399) | 40408.6  (28441.6-53474.3) | 0.06 (0.05 to 0.08) | <0.001 |
| Australia | 1547567  (1288043-1908170) | 34663.53  (24983.63-46094.51) | 2447077  (2086656-2902520) | 41781.08  (30666.35-54290.36) | 0.64 (0.61 to 0.66) | <0.001 |
| Austria | 788864  (643013-935397) | 40197.28  (28912.98-52391.38) | 796108  (660413-950291) | 42313.63  (31383.7-54526.35) | 0.12 (0.04 to 0.19) | 0.001 |
| Azerbaijan | 773408  (623254-933418) | 39956.78  (27819.45-53085.87) | 1109735  (911094-1357514) | 41144.94  (28834.52-54574.26) | 0.09 (0.07 to 0.1) | <0.001 |
| Bahamas | 29764  (24216-36062) | 40217.98  (27537.37-53857) | 42616  (35661-51955) | 39931.26  (28009.9-53380.22) | -0.02 (-0.04 to 0) | 0.034 |
| Bahrain | 48895  (40278-59963) | 40776.52  (28413.17-54349.5) | 135094  (113034-163766) | 41599.55  (29260.75-55167.32) | 0.07 (0.06 to 0.07) | <0.001 |
| Bangladesh | 10027593  (8177542-11969921) | 38935.33  (27582.71-51441.88) | 18782149  (15535756-22133549) | 40411.21  (29060.63-52601.76) | 0.12 (0.1 to 0.13) | <0.001 |
| Barbados | 27817  (22710-33651) | 40270.64  (27941.71-53506.65) | 28865  (23732-34748) | 41515.73  (28665.2-55223.71) | 0.09 (0.06 to 0.1) | <0.001 |
| Belarus | 1038836  (850573-1256864) | 41164.32  (29212.46-54077.3) | 838070  (703290-1023944) | 41567.77  (29375.88-54968.8) | 0.03 (0.03 to 0.04) | <0.001 |
| Belgium | 937755  (787914-1114507) | 39122.91  (28593.46-50972.99) | 959633  (796910-1141964) | 40445.62  (29408.45-52287.17) | 0.1 (0.07 to 0.14) | <0.001 |
| Belize | 17975  (14321-21612) | 40107.85  (27918.63-52148.76) | 51846  (43550-61054) | 42027.84  (29911.29-53631.87) | 0.13 (0.1 to 0.15) | <0.001 |
| Benin | 448701  (371600-539432) | 39220.57  (27280.6-52684.65) | 1336266  (1108445-1609336) | 39480.75  (28089.87-52242.09) | 0.02 (0.01 to 0.03) | 0.006 |
| Bermuda | 6904  (5579-8496) | 40991.24  (28481.13-54491.15) | 5420  (4446-6676) | 42101.9  (29776.5-55823.76) | 0.06 (0.02 to 0.08) | 0.001 |
| Bhutan | 58674  (48595-69068) | 39196.79  (28024.26-51293.39) | 84728  (70968-101272) | 40420.31  (29006.87-52822) | 0.09 (0.09 to 0.1) | <0.001 |
| Bolivia (Plurinational State of) | 580403  (469197-712885) | 37264.26  (25134.44-50762.3) | 1203772  (967767-1480043) | 38344.38  (25769.79-52222.68) | 0.1 (0.08 to 0.1) | <0.001 |
| Bosnia and Herzegovina | 404728  (327365-499645) | 34653.03  (23467.37-47827.2) | 272182  (221914-333522) | 38295.32  (26229.99-51605.48) | 0.32 (0.31 to 0.33) | <0.001 |
| Botswana | 124830  (101196-150826) | 36773.11  (25733.14-49457.52) | 247799  (211324-302842) | 36270.42  (26258.99-47871.34) | -0.03 (-0.05 to -0.02) | <0.001 |
| Brazil | 18665777  (15903274-21287603) | 47133.98  (34902.62-58361.94) | 28180718  (24124720-32211297) | 48500.55  (36525.89-59434.7) | 0.09 (0.08 to 0.1) | <0.001 |
| Brunei Darussalam | 32481  (26647-36753) | 46260.6  (34758.52-57225.19) | 57977  (48559-66273) | 47052.76  (36052.19-57782.73) | 0.05 (0.03 to 0.07) | <0.001 |
| Bulgaria | 771250  (611235-934886) | 37701.09  (25171.52-50665.23) | 553154  (437266-662429) | 40186.61  (27354.54-53462.31) | 0.21 (0.2 to 0.22) | <0.001 |
| Burkina Faso | 852759  (705548-1049540) | 39293.34  (27461.83-52357.21) | 2276133  (1899286-2764286) | 39773.48  (28336.72-52394.53) | 0.06 (0.04 to 0.07) | <0.001 |
| Burundi | 500748  (405728-611262) | 38321.81  (26580.79-52096.86) | 1234059  (1017616-1531613) | 38031.22  (26058.28-51492.35) | -0.02 (-0.03 to -0.01) | 0.003 |
| Cabo Verde | 33176  (27105-39316) | 39636.06  (27750.8-52300.81) | 61133  (50790-74452) | 40174.71  (28573.48-52882.1) | 0.07 (0.06 to 0.09) | <0.001 |
| Cambodia | 1158649  (961905-1341274) | 45477.12  (31998.09-58022.77) | 2173122  (1786627-2546321) | 47768.73  (34131.32-60522.96) | 0.18 (0.16 to 0.21) | <0.001 |
| Cameroon | 982504  (811561-1183114) | 39412.32  (28169.99-51852.63) | 3264512  (2731240-3931752) | 40174.32  (28596.84-52849.5) | 0.05 (0.04 to 0.07) | <0.001 |
| Canada | 2939939  (2368847-3583681) | 40523.25  (29411.7-53465.82) | 3214393  (2691958-3742834) | 40179.91  (30033.74-51291.03) | -0.05 (-0.08 to 0) | 0.042 |
| Central African Republic | 270189  (222846-322966) | 40410.58  (28400.93-53868.31) | 562537  (464766-681383) | 39168.28  (27936.29-51739.99) | -0.1 (-0.1 to -0.09) | <0.001 |
| Chad | 532659  (443759-648187) | 38576.79  (27205.05-51605.43) | 1607563  (1353371-1941450) | 39653  (28137.76-52288.81) | 0.09 (0.08 to 0.1) | <0.001 |
| Chile | 1424522  (1143353-1700603) | 38611.02  (26413.85-51888.93) | 1828254  (1619533-1978067) | 39688.15  (32643.08-45994.97) | 0.07 (-0.01 to 0.19) | 0.089 |
| China | 92587392  (77761677-110636861) | 27819.21  (21227.6-35918.4) | 109492932  (94293847-127969708) | 36996.67  (28332.65-46596.79) | 0.93 (0.86 to 1) | <0.001 |
| Colombia | 3328768  (2692159-4155724) | 36816.16  (25564.79-50079.31) | 4928411  (4152301-6074461) | 37661.67  (26914.6-50460.57) | 0.16 (0.07 to 0.29) | 0.002 |
| Comoros | 42062  (34342-50185) | 36860.48  (25662.06-49254.61) | 74388  (59484-88192) | 37000.34  (25482.56-48221.74) | 0.02 (0.01 to 0.03) | <0.001 |
| Congo | 237634  (199242-280909) | 39975.55  (28429.22-52511.18) | 589387  (493088-710059) | 40464.6  (28397.06-53660.59) | 0.05 (0.04 to 0.05) | <0.001 |
| Cook Islands | 1713  (1395-2084) | 36396.46  (24741.1-49151.38) | 1586  (1298-1972) | 37097.94  (25970.19-50249.26) | 0.05 (0.04 to 0.06) | <0.001 |
| Costa Rica | 329692  (275653-393063) | 41154.56  (28883.58-53867.21) | 539364  (448350-646379) | 42012.16  (30096.98-54752.08) | 0.07 (0.05 to 0.1) | <0.001 |
| Coted'Ivoire | 1141165  (946339-1394181) | 39626.4  (28218.28-52968.4) | 2714736  (2265914-3282478) | 39502.69  (28150.67-52554.1) | -0.02 (-0.03 to -0.01) | <0.001 |
| Croatia | 411253  (331507-509875) | 34442.16  (23129.33-48079.64) | 321780  (255169-383229) | 36930.46  (25283.69-49721.81) | 0.23 (0.22 to 0.25) | <0.001 |
| Cuba | 1255935  (1033886-1548885) | 40353.42  (28051.39-54296.81) | 997565  (827711-1220681) | 41205.46  (28643.58-54643.59) | 0.08 (0.04 to 0.11) | <0.001 |
| Cyprus | 79357  (66415-92784) | 40432.63  (28715.38-52785.32) | 139769  (115409-167138) | 41728.91  (30043.65-53419.79) | 0.1 (0.09 to 0.11) | <0.001 |
| Czechia | 935816  (760597-1153620) | 36953.12  (24977.66-50784.69) | 851038  (696145-1059849) | 38772.33  (26824.51-52552.78) | 0.16 (0.15 to 0.17) | <0.001 |
| Democratic People's Republic of Korea | 1203134  (891055-1566522) | 20639.2  (14053.26-29820.21) | 1411503  (1070858-1778429) | 21930.06  (14945.52-31448.43) | 0.25 (0.18 to 0.32) | <0.001 |
| Democratic Republic of the Congo | 3571101  (2971645-4395385) | 39917.21  (28440.58-52577.42) | 8749087  (7341282-10515587) | 39398.17  (28131.46-52470.98) | -0.05 (-0.08 to -0.04) | <0.001 |
| Denmark | 402667  (318566-501743) | 32360.4  (22176.67-43396.4) | 401871  (326608-483238) | 33596.07  (23538.06-44427.96) | 0.11 (0.1 to 0.12) | <0.001 |
| Djibouti | 40108  (33397-47923) | 37465.21  (26314.93-48980.11) | 119222  (93271-144476) | 37143.69  (25557.18-48562.39) | -0.01 (-0.03 to 0.01) | 0.145 |
| Dominica | 6970  (5612-8529) | 39702.27  (27486.76-53015.95) | 6617  (5455-7842) | 40307.62  (27812.93-53121.75) | 0.06 (0.05 to 0.07) | <0.001 |
| Dominican Republic | 774926  (635678-929822) | 39606.95  (27592.22-52839.57) | 1192861  (978025-1432987) | 40984.9  (29062.75-54190.05) | 0.11 (0.1 to 0.12) | <0.001 |
| Ecuador | 987943  (794660-1191020) | 38336.26  (25914.42-51757.33) | 1832820  (1484634-2174044) | 38670.94  (26230.53-51967.49) | 0.02 (0.01 to 0.03) | 0.006 |
| Egypt | 5463993  (4614102-6476122) | 41013.29  (29285.46-54243) | 11065711  (9028020-13052439) | 42253.95  (30185.39-54851.33) | 0.09 (0.08 to 0.1) | <0.001 |
| El Salvador | 560327  (467366-674322) | 41322.62  (28556.16-54881.69) | 749795  (628379-896220) | 41939.37  (29577.39-55659.57) | 0.05 (0.04 to 0.06) | <0.001 |
| Equa torial Guinea | 40683  (34357-48122) | 39651.89  (28126.66-52230.04) | 154194  (126319-178390) | 40648.78  (29370.8-52215.99) | 0.09 (0.07 to 0.11) | <0.001 |
| Eritrea | 297707  (238180-369348) | 36659.89  (24772.5-50571.33) | 627083  (485691-764182) | 37115.62  (25183.03-50734.32) | 0.01 (-0.03 to 0.06) | 0.250 |
| Es tonia | 146565  (123114-175841) | 39343.44  (28319.83-51803.5) | 102185  (82858-123867) | 39053.04  (27893.92-51542.23) | -0.02 (-0.03 to -0.01) | 0.003 |
| Eswatini | 75638  (61692-91052) | 36825.15  (25465.91-48452.01) | 120071  (100366-145642) | 36804.64  (26017.99-49134.59) | -0.01 (-0.03 to 0.01) | 0.424 |
| Ethiopia | 5165119  (4493340-5813962) | 43327.34  (34427.79-51924.11) | 12703905  (11280342-14177197) | 43331.99  (34593.66-51701.74) | 0 (0 to 0.01) | 0.041 |
| Fiji | 72504  (58138-87648) | 36288.24  (24602.21-49115.25) | 83493  (66733-101825) | 36582.75  (25102.36-50004.89) | 0.02 (0.01 to 0.03) | <0.001 |
| Finland | 358821  (286624-441628) | 30092.71  (21112.18-40729.53) | 332321  (262424-405117) | 30822.92  (21592.57-41571.11) | 0.04 (-0.03 to 0.11) | 0.282 |
| France | 6046624  (4990753-7241703) | 42140.82  (29995.58-54382.29) | 6017226  (5023621-7173148) | 43738.05  (30827.9-56562.7) | 0.12 (0.11 to 0.13) | <0.001 |
| Gabon | 95965  (80002-113851) | 40972.43  (29338.28-53433.57) | 207215  (172689-252278) | 40990.66  (29870.42-54199.56) | 0 (-0.01 to 0) | 0.372 |
| Gambia | 93150  (76670-115547) | 38773.31  (27259.53-51629.93) | 253693  (212348-304557) | 39511.07  (28125.12-52844.39) | 0.06 (0.05 to 0.07) | <0.001 |
| Georgia | 546313  (441860-680570) | 39698.4  (27530.16-53386.82) | 316123  (262867-383027) | 41441.58  (29273.65-55658.79) | 0.15 (0.13 to 0.18) | <0.001 |
| Germany | 6451343  (5018693-7725077) | 34138.52  (23179.24-44989.54) | 5740219  (4725073-6953858) | 36502.96  (25814.38-48554.39) | 0.22 (0.2 to 0.23) | <0.001 |
| Ghana | 1495229  (1259436-1809486) | 40600.53  (29362.52-53206.84) | 3788648  (3191194-4473622) | 40409.02  (29380.36-52544.5) | -0.02 (-0.04 to -0.01) | <0.001 |
| Greece | 925701  (765611-1110231) | 37044.67  (26022.99-49283.22) | 829387  (687360-1015776) | 40937.43  (28866.41-53964.92) | 0.32 (0.31 to 0.33) | <0.001 |
| Greenland | 6189  (5086-7146) | 40234.94  (29681.28-52103.95) | 5127  (4196-6009) | 40301.44  (29280.51-52357.05) | -0.07 (-0.11 to -0.04) | <0.001 |
| Grenada | 7970  (6469-9600) | 39717.68  (27484.12-53097.22) | 10587  (8731-12607) | 41322.59  (28824.54-54633.64) | 0.12 (0.12 to 0.13) | <0.001 |
| Guam | 13411  (11003-16659) | 37603.36  (25832.53-51314.7) | 13418  (11019-16836) | 37545.28  (25927.06-50943.91) | -0.02 (-0.04 to 0) | 0.031 |
| Guatemala | 781565  (645376-941066) | 41114.79  (28593.06-54318.31) | 1889427  (1590847-2300597) | 42079.46  (29399.56-56139.47) | 0.07 (0.07 to 0.08) | <0.001 |
| Guinea | 551844  (459866-668863) | 39323.29  (27971.62-51784.33) | 1347203  (1133444-1645044) | 39027.53  (27600.11-52403.46) | -0.03 (-0.04 to -0.02) | <0.001 |
| Guinea-Bissau | 87973  (70535-109174) | 36479.82  (24955.77-49835.91) | 198852  (162095-244710) | 36681.8  (25391.24-49510.72) | 0.02 (0.01 to 0.04) | 0.004 |
| Guyana | 82833  (68075-101037) | 39211.18  (27217.1-52422.81) | 83693  (67639-100187) | 40769.29  (28390.56-54409.85) | 0.11 (0.09 to 0.12) | <0.001 |
| Haiti | 639417  (515413-763057) | 40063.9  (27717.7-52489.74) | 1438764  (1191685-1690208) | 40104.54  (28598.9-51966.83) | 0 (0 to 0.01) | 0.055 |
| Honduras | 441938  (365564-542648) | 40325.69  (28273.79-53446.79) | 1203582  (1004848-1468813) | 41492.8  (29035.58-54539.55) | 0.09 (0.08 to 0.1) | <0.001 |
| Hungary | 943504  (762254-1161268) | 37869.44  (25681.44-51690.92) | 802022  (667219-943791) | 39186.39  (26782.8-52130.33) | 0.14 (0.11 to 0.17) | <0.001 |
| Iceland | 21334  (17334-25460) | 32597.74  (22688.3-43885.41) | 26237  (21349-31098) | 34367.14  (23927.65-45338.41) | 0.16 (0.12 to 0.19) | <0.001 |
| India | 91400991  (79918415-104280884) | 44213.45  (34044.8-54672.69) | 174235083  (154223901-196602159) | 45719.05  (36372.88-55002.97) | 0.09 (0.07 to 0.11) | <0.001 |
| Indonesia | 23749232  (20487953-27152100) | 48548.67  (36418.62-59567.25) | 36793744  (32040021-41810235) | 49195.52  (37501.11-59760.35) | 0.03 (0.01 to 0.05) | 0.001 |
| Iran (Islamic Republic of) | 6001955  (5332808-6722258) | 45870.57  (36865.68-54870.92) | 10413461  (9202965-11846232) | 45978.17  (36761.93-54897.63) | 0.01 (0 to 0.02) | 0.091 |
| Iraq | 1658724  (1358979-2005078) | 39963.98  (27201.48-53271.89) | 4374491  (3637025-5217769) | 41064.54  (28624.88-54259.78) | 0.09 (0.08 to 0.1) | <0.001 |
| Ireland | 359249  (300331-428466) | 40489.75  (28719.62-53665.31) | 473259  (406053-558820) | 42641.96  (30765.24-54555) | 0.16 (0.14 to 0.17) | <0.001 |
| Israel | 512202  (419255-619056) | 41666.9  (28771.64-55206.09) | 978486  (820967-1162010) | 44471.92  (31140.87-57515.75) | 0.18 (0.14 to 0.21) | <0.001 |
| Italy | 5991482  (5187976-6963857) | 42313.2  (31860.97-53333.22) | 4820448  (4191835-5620265) | 42949.42  (32904.45-52999.65) | 0.04 (0.02 to 0.06) | 0.000 |
| Jamaica | 241959  (194237-290543) | 39159.88  (27351-52013.27) | 316840  (261848-380717) | 41006.72  (28301.7-54325.53) | 0.14 (0.11 to 0.16) | <0.001 |
| Japan | 13531866  (11974658-14959868) | 43822.72  (35697.85-51603.15) | 8498463  (7435109-9525902) | 37767.91  (30229.8-45076.74) | -0.48 (-0.51 to -0.45) | <0.001 |
| Jordan | 340319  (280259-411499) | 39732.87  (26857.81-53236.91) | 1250415  (1045235-1497343) | 40032.72  (27508.34-53351.47) | 0.03 (0.01 to 0.03) | <0.001 |
| Kazakhstan | 1673882  (1371360-2022444) | 40166.63  (27915.09-53213.45) | 1941999  (1610103-2359952) | 41781.97  (29013.91-55385.88) | 0.11 (0.09 to 0.12) | <0.001 |
| Kenya | 2364321  (2083218-2674264) | 42688.91  (33901.62-51234.36) | 5871251  (5212663-6591836) | 42215.8  (33826.47-50197.81) | -0.03 (-0.04 to -0.03) | <0.001 |
| Kiribati | 6738  (5520-8273) | 34945.98  (23960.71-47861.52) | 11622  (9240-14058) | 35931.3  (24283.56-49390.29) | 0.06 (0.04 to 0.1) | <0.001 |
| Kuwait | 174739  (141196-215028) | 40793.5  (28192.41-54248.89) | 585940  (475931-710722) | 41142.18  (29062.02-54139.03) | 0.03 (0.02 to 0.04) | <0.001 |
| Kyrgyzstan | 427282  (350862-512893) | 39593.21  (27351.03-52909.65) | 696132  (572018-842185) | 40255.37  (28157.37-53354.7) | 0.05 (0.05 to 0.06) | <0.001 |
| Lao People's Democratic Republic | 440138  (367401-520965) | 44509.48  (31872.05-57017.8) | 913986  (782260-1068391) | 45433.12  (33863.45-57901.89) | 0.08 (0.06 to 0.09) | <0.001 |
| Latvia | 263314  (218323-319867) | 41342.56  (29246.14-54429.77) | 155888  (127278-191589) | 42059.37  (29952.75-55115.81) | 0.05 (0.01 to 0.07) | 0.012 |
| Lebanon | 303332  (254611-364369) | 39974.83  (27716.8-53092.24) | 597925  (488788-716911) | 40584.88  (27726.43-53724.35) | 0.05 (0.04 to 0.06) | <0.001 |
| Lesotho | 140152  (116490-167154) | 36132.73  (25412.53-49205.41) | 189325  (153473-232326) | 35965.26  (25168.84-48533.19) | -0.02 (-0.03 to 0) | 0.089 |
| Liberia | 224720  (186630-269551) | 38643.21  (27166.4-51526.89) | 559785  (471137-669489) | 39102.56  (28125.41-51614.53) | 0.03 (0.02 to 0.04) | <0.001 |
| Libya | 376328  (312153-446278) | 40131.77  (27955.74-53180.68) | 803648  (648359-984971) | 40823.22  (27894.52-54828.41) | 0.06 (0.05 to 0.07) | <0.001 |
| Lithuania | 371498  (300497-449833) | 40496.21  (28184.58-53750.95) | 235328  (193037-291121) | 42401.46  (29754.18-55917.64) | 0.14 (0.13 to 0.15) | <0.001 |
| Luxembourg | 38248  (32521-45909) | 40216.23  (29152.29-52805.79) | 62483  (52374-75495) | 42172.45  (30297.7-54492.65) | 0.16 (0.15 to 0.18) | <0.001 |
| Madagascar | 961505  (743416-1175567) | 35488.51  (23307.65-49379.49) | 2854019  (2325560-3424542) | 38529.22  (26240.25-53060.91) | 0.26 (0.24 to 0.27) | <0.001 |
| Malawi | 905733  (745574-1133018) | 38585.8  (26638.12-52539.15) | 2006097  (1629914-2435392) | 38543.11  (26619.07-52044.25) | -0.02 (-0.05 to 0.01) | 0.132 |
| Malaysia | 1658125  (1324178-2018604) | 35749.61  (24793.03-48537.65) | 3041060  (2391614-3700594) | 35787.38  (25115.22-48134.39) | 0.03 (0 to 0.05) | 0.020 |
| Maldives | 22022  (18443-26121) | 44634.72  (32315.07-57788.45) | 51440  (42865-59987) | 45059.97  (32747.27-57106.36) | 0.03 (0.03 to 0.04) | <0.001 |
| Mali | 766992  (629145-924960) | 38594.33  (26859.74-50991.4) | 2259637  (1865134-2708856) | 39608.47  (27962.72-52522.4) | 0.08 (0.08 to 0.09) | <0.001 |
| Malta | 37051  (30621-43763) | 40444.58  (28031.98-52863.7) | 37166  (30904-44248) | 41599.68  (30060.46-53448.72) | 0.09 (0.09 to 0.1) | <0.001 |
| Marshall Islands | 3554  (2858-4288) | 34952.38  (23921.54-46993.24) | 5338  (4339-6690) | 35794.33  (24595.19-49085.62) | 0.08 (0.07 to 0.08) | <0.001 |
| Mauritania | 192988  (161586-234730) | 39362.66  (27641.73-53012.41) | 441623  (368562-526706) | 39664.6  (28256.55-52450.62) | 0.03 (0.02 to 0.04) | <0.001 |
| Mauritius | 135761  (112184-162840) | 44661.71  (32144.41-57627.55) | 140834  (120870-161089) | 45145.43  (33867.81-56644.17) | 0.04 (0.02 to 0.05) | <0.001 |
| Mexico | 10249709  (8989898-11565324) | 44802.06  (34816.1-54919.06) | 15577989  (13710373-17625241) | 44750.66  (35199.82-54901.49) | 0 (-0.01 to 0) | 0.148 |
| Micronesia (Federated States of) | 8553  (6851-10313) | 35739.95  (24215.04-48939.42) | 9399  (7591-11531) | 35418.95  (24251.52-48610.22) | -0.03 (-0.04 to -0.02) | 0.000 |
| Monaco | 2753  (2227-3305) | 41868.14  (29947.3-54137.82) | 2908  (2437-3371) | 43238.08  (31501.94-54527.67) | 0.09 (0.08 to 0.1) | <0.001 |
| Mongolia | 209227  (168396-261170) | 39357.05  (26900.37-53630.95) | 338970  (278323-410606) | 40805.1  (28369.98-54492.96) | 0.12 (0.11 to 0.12) | <0.001 |
| Montenegro | 54862  (44449-66413) | 35104.8  (23534.9-48229.21) | 54199  (43658-65766) | 38382.77  (25849.49-51940.22) | 0.29 (0.29 to 0.3) | <0.001 |
| Morocco | 2946754  (2502477-3428483) | 44885.91  (33309.7-56549.39) | 4248515  (3598350-4942137) | 44188.8  (33037.82-55434.95) | -0.03 (-0.06 to -0.01) | 0.020 |
| Mozambique | 989592  (802361-1219754) | 30571.94  (21149.58-41883.25) | 2983452  (2455955-3654557) | 37733.44  (26009.48-50905.25) | 0.69 (0.65 to 0.73) | <0.001 |
| Myanmar | 4954475  (4265736-5687151) | 46194.19  (34824.97-57805.93) | 6952613  (5926204-8078848) | 45946.36  (34708.28-58256.42) | 0 (-0.02 to 0.02) | 0.762 |
| Namibia | 124100  (100573-150128) | 35127.27  (24817.95-46816.46) | 247148  (202592-300216) | 36477.61  (25760.47-48992.47) | 0.13 (0.11 to 0.16) | <0.001 |
| Nauru | 919  (755-1148) | 36786.02  (25564.55-50406.99) | 1045  (846-1281) | 35983.63  (24588.52-49058.32) | -0.05 (-0.08 to -0.02) | 0.001 |
| Nepal | 1833226  (1502333-2219637) | 38875.36  (27772.97-51833.37) | 3642733  (2974245-4405360) | 39231.23  (27841.47-51793.02) | 0.02 (-0.02 to 0.05) | 0.344 |
| Netherlands | 1272020  (1010098-1500561) | 32943.87  (22631.76-43563.8) | 1146867  (928628-1357677) | 33154.63  (23196.74-43711.11) | 0.01 (-0.01 to 0.02) | 0.336 |
| New Zealand | 416603  (365491-480395) | 46072.56  (35270.83-57214.42) | 546423  (457851-647944) | 46512.51  (34976.21-58349.29) | 0.04 (0.03 to 0.05) | <0.001 |
| Nicaragua | 379441  (313355-461710) | 40243.3  (27963.04-53836.44) | 751752  (628325-911346) | 41137.33  (28942.61-54454.71) | 0.08 (0.05 to 0.1) | 0.001 |
| Niger | 691294  (570157-813862) | 38242.51  (26867.6-51258.96) | 2150248  (1746822-2632060) | 38402.15  (26561.09-51805.55) | 0.02 (0.01 to 0.03) | 0.000 |
| Nigeria | 9646566  (8709246-10575695) | 44755.27  (36952.85-52384.7) | 25942806  (23142510-28338919) | 42742.12  (35400.47-49843.57) | -0.15 (-0.16 to -0.15) | <0.001 |
| Niue | 179  (145-223) | 35930.98  (24412.24-48984.74) | 139  (113-169) | 36821.88  (25479.33-49622.47) | 0.08 (0.06 to 0.09) | <0.001 |
| North Macedonia | 175974  (143600-216890) | 34571.71  (23444.14-47919.01) | 192927  (154777-236303) | 37407.23  (25574.39-50312.24) | 0.27 (0.25 to 0.3) | <0.001 |
| Northern Mariana Islands | 5381  (4309-6602) | 37697.33  (26170.76-50418.57) | 4122  (3425-4931) | 37231.83  (25716.78-49818.93) | -0.04 (-0.05 to -0.04) | <0.001 |
| Norway | 469065  (409623-531206) | 44961.04  (34837.77-54849.5) | 543242  (477357-615630) | 46226.51  (36128.01-55851.75) | 0.09 (0.08 to 0.09) | <0.001 |
| Oman | 136477  (113784-162082) | 39419.41  (27094.05-51990.89) | 406152  (331436-487946) | 40017.27  (27774.47-52749.57) | 0.06 (0.05 to 0.07) | <0.001 |
| Pakistan | 11350350  (9902401-12766588) | 46687.49  (36220.09-57339.99) | 28691333  (24907061-32261706) | 46362.75  (35643.4-56734.16) | -0.02 (-0.04 to -0.02) | <0.001 |
| Palau | 1505  (1253-1838) | 36060.81  (24739.23-49100.54) | 1331  (1098-1611) | 37267.95  (25554.25-50184.88) | 0.12 (0.08 to 0.15) | <0.001 |
| Palestine | 185845  (154288-223534) | 40714.8  (28920.91-53667.65) | 553725  (465871-655725) | 41562.61  (29788.74-54013.35) | 0.08 (0.05 to 0.11) | <0.001 |
| Panama | 245436  (200179-291909) | 39294.98  (26747.86-52567.3) | 438980  (359271-527332) | 41032.65  (27937.35-54865.12) | 0.14 (0.13 to 0.15) | <0.001 |
| Papua New Guinea | 353369  (279432-444020) | 35516.98  (23907.79-50027.8) | 947099  (753899-1179250) | 35648.48  (24403.34-48190.29) | 0 (-0.02 to 0.02) | 0.853 |
| Paraguay | 357981  (286535-434813) | 37371.18  (25338.59-50456.2) | 706495  (580563-866166) | 37009.39  (25175.59-49949.67) | -0.06 (-0.08 to -0.04) | <0.001 |
| Peru | 2111243  (1694522-2619837) | 37983.45  (25575.1-52047.43) | 3748589  (3044609-4595425) | 38955.97  (26413.19-53216.16) | 0.06 (0.01 to 0.09) | 0.015 |
| Philippines | 7425151  (6617618-8285805) | 46530  (37172.59-55490.1) | 13943052  (12531035-15499248) | 47012.31  (38384.38-55366.71) | 0.04 (0.03 to 0.04) | <0.001 |
| Poland | 3940144  (3478858-4503678) | 42574.82  (33825.98-51458.37) | 3706640  (3301074-4165024) | 44109.67  (35427.91-52711.84) | 0.13 (0.11 to 0.15) | <0.001 |
| Portugal | 1013296  (848519-1229531) | 40267.81  (28721.38-53251.76) | 913527  (746545-1090408) | 42050.05  (30020.49-53841.94) | 0.13 (0.11 to 0.15) | <0.001 |
| Puer to Rico | 395123  (327320-475913) | 41207.33  (28648.56-54489.2) | 307164  (253805-368892) | 41847.58  (29765.24-55225.39) | 0.06 (0.05 to 0.07) | <0.001 |
| Qatar | 32708  (27048-39021) | 40851.24  (28770.86-54124.86) | 230914  (192607-280217) | 42023.77  (30124.38-54596.97) | 0.1 (0.09 to 0.13) | <0.001 |
| Republic of Korea | 6502680  (5633072-7354840) | 50693.66  (39237.33-61341.75) | 5718346  (4989288-6496627) | 51060.49  (40155.27-61407.19) | 0.03 (0.02 to 0.03) | <0.001 |
| Republic of Moldova | 459676  (377273-552673) | 41038.16  (28817.75-54400.41) | 345980  (287837-420584) | 41115.01  (28545.71-54453.27) | 0 (-0.01 to 0.01) | 0.516 |
| Romania | 1922366  (1534633-2351725) | 34238.15  (22607.07-47629.88) | 1468019  (1180504-1792045) | 37410.88  (25368.41-50948.84) | 0.3 (0.29 to 0.32) | <0.001 |
| Russian Federation | 16427069  (13970573-19093350) | 44638.36  (33635.03-55700.52) | 14545828  (12521502-16808300) | 45144.74  (34539.92-55580.71) | 0.04 (0.03 to 0.04) | <0.001 |
| Rwanda | 655876  (541325-788849) | 38979.16  (26735.9-51847.25) | 1399195  (1137638-1691295) | 38758.78  (26677.95-51605.15) | 0 (-0.01 to 0.01) | 0.315 |
| Saint Kitts and Nevis | 4167  (3412-5009) | 40370.11  (28107.12-53766.88) | 6460  (5372-7813) | 42094.03  (29651.81-55673.27) | 0.12 (0.11 to 0.13) | <0.001 |
| Saint Lucia | 14060  (11598-17183) | 39756.92  (27559.35-53216.65) | 18329  (15404-21881) | 40985.93  (28537.62-53872.51) | 0.08 (0.06 to 0.09) | <0.001 |
| Saint Vincent and the Grenadines | 9383  (7555-11481) | 35118.85  (23238.98-48464.97) | 10641  (8712-13018) | 38634.6  (26577.77-51651.2) | 0.3 (0.28 to 0.31) | <0.001 |
| Samoa | 13795  (11054-16834) | 36111.52  (24796.04-49449.86) | 17994  (14712-22130) | 36269.67  (24847.02-50586.55) | 0.01 (0.01 to 0.02) | <0.001 |
| San Marino | 2535  (2140-3039) | 40622.75  (28694.1-53532.96) | 2825  (2348-3363) | 42264.47  (30191.49-55465.43) | 0.13 (0.12 to 0.14) | <0.001 |
| Sao Tome and Principe | 10463  (8652-12434) | 38788.06  (27466.78-51378.54) | 22942  (19211-26987) | 39935.44  (28360.82-51992.54) | 0.09 (0.07 to 0.1) | <0.001 |
| Saudi Arabia | 1453004  (1155974-1725263) | 44001.78  (30113.72-57684.58) | 4584964  (3756647-5399100) | 45244.68  (31209.69-58747.53) | 0.1 (0.09 to 0.1) | <0.001 |
| Senegal | 731835  (608667-862547) | 38935.43  (27845.76-50529.4) | 1638514  (1358476-1932114) | 39354.53  (28538.92-50430.06) | 0.04 (0.03 to 0.05) | <0.001 |
| Serbia | 753485  (605693-933366) | 32488.88  (22252.83-44987.97) | 689911  (561287-858918) | 34968.88  (23995.74-47448.22) | 0.26 (0.18 to 0.32) | <0.001 |
| Seychelles | 8433  (7035-10036) | 45182.21  (32749.61-58248.65) | 10749  (9145-12505) | 45202.69  (33313.36-57122.52) | 0.01 (0 to 0.02) | 0.106 |
| Sierra Leone | 367285  (296553-435211) | 34501.6  (23633.15-45918.46) | 947615  (773640-1093766) | 38544.08  (27554.53-49837.18) | 0.35 (0.34 to 0.37) | <0.001 |
| Singapore | 445676  (376696-509435) | 47243.06  (35429.69-58513.02) | 641083  (527804-760465) | 47184.33  (36323.12-58347.21) | 0 (-0.02 to 0.02) | 0.791 |
| Slovakia | 488911  (382539-596089) | 37081.78  (24494.15-51139.21) | 463403  (373656-569377) | 38248.35  (26369.13-51494.73) | 0.11 (0.1 to 0.13) | <0.001 |
| Slovenia | 197816  (162768-242853) | 40159.68  (28161.15-53297.48) | 169534  (136019-204853) | 41778.99  (29145.81-55729.79) | 0.13 (0.12 to 0.15) | <0.001 |
| Solomon Islands | 27264  (21659-33621) | 35238.79  (23794.26-47884.29) | 60950  (48638-75044) | 35002.15  (24029.35-48423.41) | -0.01 (-0.03 to 0) | 0.032 |
| Somalia | 647253  (531825-787000) | 37544.99  (25597.56-50725.07) | 1854272  (1506996-2232713) | 37147.63  (25495.73-50091.67) | -0.05 (-0.08 to -0.02) | 0.002 |
| South Africa | 4199715  (3714726-4778944) | 41491.15  (32399.81-50994.24) | 6574625  (5812269-7549698) | 42655.51  (33638.43-52077.95) | 0.08 (0.07 to 0.09) | <0.001 |
| South Sudan | 534996  (442808-649837) | 39549.8  (27818.45-52675.33) | 918907  (751137-1103030) | 38814.38  (26747.48-51661.19) | -0.06 (-0.06 to -0.05) | <0.001 |
| Spain | 3808653  (3239997-4430025) | 39421.84  (27730.51-51730.24) | 3934657  (3284228-4725572) | 42845.56  (30545.78-55303.6) | 0.26 (0.25 to 0.28) | <0.001 |
| Sri Lanka | 2078987  (1742923-2464240) | 44680.41  (32070.76-57449.15) | 2515583  (2150244-2977951) | 45111.66  (32947.54-57754.64) | 0.04 (0.02 to 0.04) | <0.001 |
| Sudan | 1849389  (1493658-2262113) | 38711.25  (26543.28-52685.59) | 4565811  (3746873-5570050) | 39796.81  (27340.01-52659.37) | 0.09 (0.07 to 0.11) | <0.001 |
| Suriname | 43195  (36267-50988) | 43215.67  (31348.5-55853.32) | 62771  (52729-74466) | 43587.2  (31315.95-55815.47) | 0.01 (-0.03 to 0.04) | 0.597 |
| Sweden | 730261  (599517-896344) | 36806.77  (26447.21-48870.91) | 877449  (747821-1015937) | 41679.45  (30566.94-52821.7) | 0.4 (0.39 to 0.41) | <0.001 |
| Switzerland | 636501  (527549-758643) | 36990.33  (25582.39-49437.47) | 723761  (590270-865512) | 38901.73  (27161.55-51311.49) | 0.16 (0.16 to 0.17) | <0.001 |
| Syrian Arab Republic | 1104766  (906882-1352240) | 39106.13  (26590.9-52915.61) | 1515713  (1237923-1857600) | 39577.18  (26899.98-53399.05) | 0.04 (0.03 to 0.05) | <0.001 |
| Taiwan (Province of China) | 1213215  (923490-1579077) | 21451.67  (14763.75-31240.25) | 1085229  (836541-1342779) | 20727.69  (14148.96-29322.14) | -0.07 (-0.14 to -0.01) | 0.040 |
| Tajikistan | 504206  (412820-613057) | 40205.03  (27877.78-54439.48) | 1031565  (831080-1269286) | 40117.17  (27665.46-53416.68) | -0.03 (-0.05 to -0.01) | 0.019 |
| Thailand | 7111126  (5858394-8110891) | 43621.65  (31171.83-55630.26) | 7203040  (6031269-8580120) | 45636.95  (33673.53-58367.57) | 0.15 (0.14 to 0.16) | <0.001 |
| Timor-Leste | 78488  (63928-94075) | 41154.61  (28499.07-54050.68) | 151379  (126485-179389) | 42499.33  (30022.77-55526.27) | 0.11 (0.1 to 0.13) | <0.001 |
| Togo | 365225  (304810-428651) | 39441.27  (27991.77-51355.2) | 872639  (720626-1023894) | 39059.53  (28050.08-50101) | -0.04 (-0.05 to -0.03) | <0.001 |
| Tokelau | 127  (102-153) | 35212.16  (24029.47-47850.72) | 115  (95-138) | 35904.12  (24690.13-48989.59) | 0.06 (0.05 to 0.08) | <0.001 |
| Tonga | 8232  (6573-10095) | 35687.61  (24062.72-48938.79) | 9273  (7512-11648) | 36182.24  (24555.53-50310.75) | 0.03 (0.02 to 0.05) | 0.002 |
| Trinidad and tobago | 126465  (103170-157321) | 40536.85  (28094.85-54783.69) | 138069  (114948-162005) | 42252.8  (30323.26-54428.41) | 0.14 (0.13 to 0.14) | <0.001 |
| Tunisia | 821553  (675088-976247) | 39221.62  (27064.36-52040.94) | 1214762  (1002270-1443261) | 40339.56  (28297.68-53044.3) | 0.1 (0.08 to 0.12) | <0.001 |
| Turkey | 5599409  (4517081-6846087) | 38689.11  (26096.42-52267.69) | 8472596  (6986554-10306704) | 39557.72  (26901.51-52699.44) | 0.07 (0.06 to 0.08) | <0.001 |
| Turkmenistan | 360159  (292189-450563) | 39220.73  (27302.11-52919.29) | 525341  (441066-628851) | 41693.23  (29589.85-54843.2) | 0.19 (0.17 to 0.21) | <0.001 |
| Tuvalu | 880  (721-1080) | 35914.13  (24255.74-49350.42) | 1040  (825-1301) | 35196.17  (24059.62-48391.18) | -0.03 (-0.06 to 0) | 0.034 |
| Uganda | 1688986  (1446677-1964609) | 41713.61  (30646.44-54176.82) | 4560708  (3773696-5386731) | 41904.51  (30178.78-54882.62) | -0.03 (-0.11 to 0.03) | 0.246 |
| Ukraine | 5798105  (4987735-6682852) | 46384.76  (33988.1-58142.82) | 4438303  (3675627-5171492) | 46263.97  (34003.86-58330.89) | 0 (-0.02 to 0.03) | 0.539 |
| United Arab Emirates | 148252  (119793-177501) | 41838.81  (29597.34-54803.19) | 674498  (536198-810706) | 41623.03  (29117.61-54443.28) | -0.02 (-0.02 to -0.01) | <0.001 |
| United Kingdom | 6032329  (5334706-6818064) | 43063.26  (33997.31-51832.24) | 6479926  (5764665-7228760) | 43818.02  (35324.3-51716.97) | 0.05 (0.02 to 0.06) | <0.001 |
| United Republic of Tanzania | 1983672  (1611369-2496623) | 31433.05  (21701.33-43470.77) | 5037736  (4092576-6297859) | 32438.73  (22564.74-44526.5) | 0.1 (0.08 to 0.11) | <0.001 |
| United States of America | 30508411  (26877327-34400041) | 46035.35  (36528.97-55685.88) | 34220033  (30284179-38145585) | 46100.04  (37091.92-55265.01) | 0 (-0.02 to 0.01) | 0.434 |
| United States Virgin Islands | 11642  (9712-13924) | 41007.07  (28639.07-54538.84) | 7055  (5871-8380) | 42275.22  (30082.42-55618.26) | 0.08 (0.06 to 0.09) | <0.001 |
| Uruguay | 288131  (237783-345624) | 38400.71  (26568.08-50979.65) | 330449  (275746-401504) | 40413.56  (28020.11-53449.61) | 0.16 (0.14 to 0.17) | <0.001 |
| Uzbekistan | 1998697  (1619323-2397098) | 39366.06  (27298.54-52416.36) | 3545512  (2886397-4344669) | 40144.29  (28362.23-53613.96) | 0.07 (0.06 to 0.08) | <0.001 |
| Vanuatu | 12882  (10592-16131) | 35604.19  (24099.36-48882.85) | 28439  (22834-34693) | 35583.88  (24220.19-49035.93) | -0.01 (-0.01 to 0) | 0.021 |
| Venezuela (Bolivarian Republic of) | 2255610  (1872932-2624182) | 45571.72  (32025.62-58664.94) | 3091784  (2576192-3601704) | 45847.66  (32046.71-59225.85) | 0.02 (0.01 to 0.02) | 0.002 |
| Viet Nam | 7964585  (6679847-9374940) | 45186.99  (32783.11-57780.28) | 11496040  (9702245-13692526) | 45781.42  (34011.95-58651.3) | 0.06 (0.05 to 0.07) | <0.001 |
| Yemen | 986161  (781118-1240670) | 34976.48  (23797.79-48379.07) | 3064077  (2538341-3782969) | 36006.82  (24837.66-48976.92) | 0.1 (0.08 to 0.12) | <0.001 |
| Zambia | 732899  (609801-861236) | 36351.52  (25572.49-47853.01) | 2011960  (1692839-2404815) | 37827.85  (26944.71-49311.89) | 0.12 (0.11 to 0.14) | <0.001 |
| Zimbabwe | 955901  (800086-1112047) | 37413.52  (26781.07-49132.74) | 1532021  (1277463-1844364) | 36440.77  (26085.43-48935.95) | -0.1 (-0.16 to -0.07) | <0.001 |

AAPC: average annual percent change, ASR: age-standardized rate, WCBA: women of childbearing age.

Supplementary Table 3**.** The case number and ASR of prevalence of caries among WCBA in 1990 and 2021 at 204 countries and territories, with AAPC from 1990 to 2021

| **Location** | **1990** | | **2021** | | **AAPC (95%CI)**  **1990–2021** | ***P*** |
| --- | --- | --- | --- | --- | --- | --- |
|  | **Number (95%UI)** | **ASR (95%UI)** | **Number (95%UI)** | **ASR (95%UI)** |  |  |
| Afghanistan | 1041455  (843768-1286374) | 48155.41  (32299.11-65048.62) | 3375367  (2710547-4112385) | 47609.31  (31489.95-64268.77) | -0.04 (-0.05 to -0.03) | <0.001 |
| Albania | 452092  (366014-536311) | 54211.87  (38032.23-69806.78) | 310030  (250906-374470) | 50212.12  (33990.72-66575.43) | -0.24 (-0.25 to -0.24) | <0.001 |
| Algeria | 2578953  (2030974-3220734) | 44787.97  (29587.11-61658.4) | 5012509  (4060223-6306885) | 44410.6  (29613.89-61183.83) | -0.04 (-0.06 to -0.03) | <0.001 |
| American Samoa | 5568  (4345-6940) | 46265.94  (29164.77-63858.07) | 5510  (4489-6839) | 47269.05  (31083.07-64520.96) | 0.07 (0.06 to 0.07) | <0.001 |
| Andorra | 6251  (4818-7808) | 41323.59  (26632.08-57820.3) | 8194  (6268-10303) | 39276.45  (25150.38-55666.25) | -0.17 (-0.18 to -0.16) | <0.001 |
| Angola | 937745  (749744-1187878) | 41345.84  (26267.74-58461.5) | 3010110  (2359681-3867269) | 39836.3  (25136.65-56725.15) | -0.12 (-0.13 to -0.12) | <0.001 |
| Antigua and Barbuda | 7653  (6157-9321) | 47144.4  (31793.12-62961.09) | 11117  (9076-13633) | 45510.15  (30331.91-61275.36) | -0.12 (-0.13 to -0.12) | <0.001 |
| Argentina | 3900809  (3158135-4727439) | 48833.45  (33307.26-64914.57) | 5811688  (4767745-6985099) | 48419.51  (32586.57-64159.24) | -0.04 (-0.07 to -0.02) | <0.001 |
| Armenia | 415940  (337199-507812) | 47878.05  (31418.09-64056.44) | 348290  (278611-426373) | 46547.84  (30930.04-62707.46) | -0.09 (-0.09 to -0.08) | <0.001 |
| Australia | 1871258  (1534783-2224101) | 41772.26  (29114.35-55789.07) | 2138163  (1634726-2783859) | 35111.2  (22200.5-50880.8) | -0.57 (-0.6 to -0.54) | <0.001 |
| Austria | 640601  (482891-851138) | 31984.35  (19685.83-48012.94) | 605975  (461356-793918) | 30216.38  (19303.24-44823.92) | -0.22 (-0.26 to -0.19) | <0.001 |
| Azerbaijan | 876123  (696809-1094845) | 46771.79  (30621.95-63266.24) | 1273141  (1027692-1561435) | 45827.95  (30706.85-61981.63) | -0.08 (-0.11 to -0.05) | <0.001 |
| Bahamas | 36003  (29157-43515) | 49938.59  (33619.48-65685.82) | 52666  (43156-63145) | 48713.31  (32957.81-64312.59) | -0.08 (-0.09 to -0.07) | <0.001 |
| Bahrain | 50413  (39669-63860) | 43721.63  (28667.45-60591.38) | 137494  (109178-173230) | 41970.74  (27353.04-58965.94) | -0.13 (-0.14 to -0.12) | <0.001 |
| Bangladesh | 10046077  (7738032-12610927) | 41179.58  (26293.07-58002.39) | 18197607  (14051694-23115108) | 39596.55  (25504.38-56663.65) | -0.12 (-0.13 to -0.12) | <0.001 |
| Barbados | 32117  (25660-39799) | 46746.27  (31200.82-62831.02) | 33427  (27152-40474) | 46489.55  (31050.21-62721.79) | -0.02 (-0.03 to -0.01) | <0.001 |
| Belarus | 1094827  (870046-1353930) | 42839.26  (27948.02-58790.57) | 910502  (705455-1139878) | 42163.33  (27421.86-58548.58) | -0.07 (-0.09 to -0.05) | <0.001 |
| Belgium | 860030  (663077-1117360) | 34932.74  (22242.88-51430.48) | 836805  (640754-1094402) | 33197.62  (21052.28-49506.8) | -0.19 (-0.22 to -0.17) | <0.001 |
| Belize | 20144  (16149-24436) | 49128.9  (33103.57-64924.51) | 48435  (39533-58732) | 40366.21  (27496.94-55938.2) | -0.62 (-0.65 to -0.58) | <0.001 |
| Benin | 478750  (372598-589296) | 43863.27  (28209.43-60700.15) | 1373080  (1062312-1703239) | 42624.38  (27637.88-59663.69) | -0.11 (-0.14 to -0.08) | <0.001 |
| Bermuda | 7885  (6357-9682) | 45314.85  (29605.12-61626.98) | 5937  (4818-7344) | 43409.85  (28516.31-59900.63) | -0.15 (-0.17 to -0.14) | <0.001 |
| Bhutan | 57355  (44386-72501) | 40581.6  (25510.76-58232.55) | 78536  (60063-99960) | 37959.03  (24100.12-55080.99) | -0.2 (-0.22 to -0.18) | <0.001 |
| Bolivia (Plurinational State of) | 800164  (655310-959990) | 52783.6  (35980.45-68740.55) | 1598560  (1327754-1921594) | 51321.95  (35582.58-67117.95) | -0.06 (-0.1 to -0.02) | 0.007 |
| Bosnia and Herzegovina | 655473  (550539-762443) | 56227.58  (39623.65-70422.41) | 369477  (304039-437676) | 50096.39  (33005.22-66314.11) | -0.38 (-0.38 to -0.37) | <0.001 |
| Botswana | 122029  (92135-156205) | 38365.23  (24081.61-55635.27) | 247310  (189815-310761) | 36235.76  (22605.21-52542.5) | -0.19 (-0.19 to -0.18) | <0.001 |
| Brazil | 15086328  (12267749-18825487) | 38934.46  (26085.15-54486.13) | 22701092  (18612989-28239493) | 38323.13  (26127.21-53317.38) | -0.05 (-0.08 to -0.03) | 0.001 |
| Brunei Darussalam | 16335  (13040-20883) | 23324.23  (15823.69-34002.84) | 29489  (22812-37947) | 23764.26  (16066.72-34285.43) | 0.06 (0.05 to 0.07) | <0.001 |
| Bulgaria | 1118215  (924915-1329665) | 53872.39  (37097.56-69269.93) | 739646  (605111-891618) | 51260.08  (34680.82-67249.34) | -0.16 (-0.16 to -0.15) | <0.001 |
| Burkina Faso | 898966  (721912-1102145) | 43262.74  (27734.84-60345.25) | 2296079  (1762801-2889038) | 42151.51  (26725.42-58690.64) | -0.12 (-0.15 to -0.09) | <0.001 |
| Burundi | 585982  (461422-727588) | 47238.91  (30877.79-63929.77) | 1460554  (1169223-1802168) | 47634.9  (31899.24-63854.88) | 0.02 (0.02 to 0.03) | <0.001 |
| Cabo Verde | 34102  (26836-43561) | 43338.33  (28412.47-60320.96) | 61591  (48252-78140) | 40973.28  (26144.78-57810.34) | -0.19 (-0.21 to -0.18) | <0.001 |
| Cambodia | 1144209  (935102-1381189) | 46243.07  (31191.32-61947.24) | 1984993  (1605126-2437725) | 44036.02  (29643.98-60068.54) | -0.16 (-0.17 to -0.16) | <0.001 |
| Cameroon | 991861  (766898-1248706) | 41881.59  (26661.92-58718.67) | 3295303  (2562997-4085773) | 42438.62  (27333.84-59286.05) | -0.01 (-0.04 to 0.02) | 0.360 |
| Canada | 1963740  (1441717-2658703) | 26316.75  (16319.45-40136.31) | 2097497  (1633796-2743470) | 25147.52  (16165.57-37870.61) | -0.14 (-0.17 to -0.12) | <0.001 |
| Central African Republic | 274893  (218514-339678) | 43490.57  (28685.1-60283.24) | 594464  (478925-736326) | 43558.3  (28369.8-60337.23) | 0.02 (0.01 to 0.02) | 0.000 |
| Chad | 579129  (454410-709408) | 43850.74  (28465.25-60435.25) | 1635201  (1306965-2059198) | 42795.74  (27999.46-59116.48) | -0.09 (-0.1 to -0.08) | <0.001 |
| Chile | 1770689  (1441617-2139939) | 49440.72  (33592.04-65175.63) | 2356611  (2294738-2419172) | 48998.71  (45341.89-52819.14) | -0.02 (-0.1 to 0.09) | 0.676 |
| China | 90807478  (68616482-119879949) | 27342.69  (17110.96-41045.43) | 87742989  (70235319-113049835) | 28202.77  (19365.61-40386.57) | 0.12 (0.09 to 0.14) | <0.001 |
| Colombia | 3689416  (2968306-4565618) | 42864.32  (27428.89-59614.19) | 5395614  (4192351-6735194) | 40953.8  (25750.51-58463.69) | -0.38 (-0.75 to -0.15) | 0.006 |
| Comoros | 41232  (33048-50987) | 40107.84  (26927.12-55584.8) | 77324  (61138-96671) | 39895.3  (26214.13-56206.94) | -0.01 (-0.02 to 0) | 0.004 |
| Congo | 224565  (173647-284401) | 40572.51  (26075.89-57792.77) | 568942  (432688-722651) | 39974.11  (25149.81-56913.92) | -0.05 (-0.05 to -0.04) | <0.001 |
| Cook Islands | 2154  (1678-2688) | 46995.85  (30623.98-64048.01) | 1967  (1557-2449) | 45697.46  (29412.85-62476.88) | -0.1 (-0.11 to -0.09) | <0.001 |
| Costa Rica | 348073  (277206-424801) | 45268.7  (29767.03-61634.56) | 567486  (457545-697416) | 43406.6  (29009.19-59358.93) | -0.13 (-0.14 to -0.13) | <0.001 |
| Coted'Ivoire | 1149783  (900218-1468844) | 42187.58  (26755.22-59655.6) | 2762534  (2147178-3512465) | 41666.67  (26854.87-58876.86) | -0.04 (-0.05 to -0.03) | <0.001 |
| Croatia | 676710  (562756-794179) | 56389.82  (39518.9-70911.82) | 473531  (388347-566277) | 52298.16  (35965.52-67795.4) | -0.24 (-0.25 to -0.23) | <0.001 |
| Cuba | 1479256  (1213564-1775941) | 48528.58  (33141.65-64138.32) | 1180372  (967841-1446463) | 46752.84  (31583.47-62761.39) | -0.1 (-0.14 to -0.07) | <0.001 |
| Cyprus | 86370  (68288-109083) | 43404.32  (28551.33-59699.39) | 151190  (118801-190619) | 40662  (26007.28-56806.47) | -0.19 (-0.21 to -0.18) | <0.001 |
| Czechia | 1322155  (1059689-1583451) | 51440.39  (34694.88-67533.73) | 1136543  (925184-1360656) | 49155.55  (33353.89-65355.12) | -0.14 (-0.15 to -0.13) | <0.001 |
| Democratic People's Republic of Korea | 1025054  (698518-1452128) | 17658.32  (9862.99-29699.13) | 1247486  (894592-1698177) | 19168.56  (10628.42-31487.39) | 0.29 (0.25 to 0.32) | <0.001 |
| Democratic Republic of the Congo | 3558649  (2833632-4347991) | 42375.82  (27448.43-59174.48) | 9057798  (7367701-11188950) | 43192.54  (27887.81-60211.22) | 0.05 (0.03 to 0.07) | <0.001 |
| Denmark | 322415  (233492-431174) | 24351.64  (14512.23-37635.96) | 347128  (266410-449249) | 27542.99  (17459.94-41432.17) | 0.38 (0.34 to 0.41) | <0.001 |
| Djibouti | 37962  (30607-47430) | 39553.41  (26248.13-54927.69) | 124939  (99575-153182) | 38719.09  (25992.03-53826.15) | -0.07 (-0.07 to -0.06) | <0.001 |
| Dominica | 8107  (6552-9837) | 48415.41  (32559.07-64569.56) | 7670  (6120-9250) | 46680.35  (31279.55-62486.87) | -0.11 (-0.11 to -0.1) | <0.001 |
| Dominican Republic | 904043  (738817-1095165) | 48648.1  (32971.39-64349.99) | 1327715  (1063141-1624068) | 45758.55  (30357.6-61952.17) | -0.21 (-0.21 to -0.2) | <0.001 |
| Ecuador | 1286434  (1039727-1547099) | 51545.43  (34849.41-67723.54) | 2380980  (1939474-2892037) | 50476.53  (34160.46-66738.7) | -0.06 (-0.08 to -0.04) | <0.001 |
| Egypt | 5297114  (4117084-6739803) | 40612.2  (26140.48-57852.95) | 10020424  (7923990-13028991) | 38640.87  (24712.85-55694.15) | -0.17 (-0.2 to -0.14) | <0.001 |
| El Salvador | 595094  (487136-729985) | 46532.78  (31615.44-62768.28) | 800692  (641337-998505) | 45013.8  (30016.25-61663.15) | -0.11 (-0.15 to -0.09) | <0.001 |
| Equatorial Guinea | 41371  (33508-51099) | 42516.75  (27663.39-59149.57) | 133874  (103532-173642) | 36978.27  (23401.1-53958.58) | -0.45 (-0.46 to -0.44) | <0.001 |
| Eritrea | 398373  (332658-481445) | 51385.02  (35048.91-66949.37) | 841554  (682326-1026589) | 51328.04  (34845.18-66892.8) | -0.04 (-0.06 to -0.01) | 0.028 |
| Estonia | 120328  (89437-156354) | 31520.14  (19249.23-47118.56) | 81523  (59085-106254) | 29492.45  (17840.36-44355.19) | -0.21 (-0.22 to -0.2) | <0.001 |
| Eswatini | 75279  (55025-96465) | 39157.11  (24176.92-56714.24) | 118734  (91845-150819) | 37725.36  (23369.18-54368.74) | -0.12 (-0.13 to -0.11) | <0.001 |
| Ethiopia | 4312562  (3721037-5038138) | 39252.85  (29799.11-50376.29) | 9895970  (8419501-11868940) | 36530.33  (27406.34-47752.63) | -0.23 (-0.24 to -0.22) | <0.001 |
| Fiji | 95317  (75862-117365) | 48821.23  (32271.38-66109.02) | 109072  (87637-133804) | 47752.11  (31207.26-64672.64) | -0.06 (-0.08 to -0.05) | <0.001 |
| Finland | 375921  (280577-496078) | 29348.55  (17340.71-44626.67) | 310422  (237820-408006) | 27034.69  (16358.91-40996.43) | -0.3 (-0.34 to -0.25) | <0.001 |
| France | 7179786  (6021749-8490534) | 49327.33  (34621.13-63581.97) | 6630785  (5443359-8027582) | 45656.83  (30587.61-61525.3) | -0.25 (-0.28 to -0.24) | <0.001 |
| Gabon | 83511  (64819-106836) | 38222.61  (23915.34-55513.73) | 183783  (144961-237438) | 37801.7  (23877.7-54504.74) | -0.04 (-0.05 to -0.03) | <0.001 |
| Gambia | 96265  (74297-121251) | 42526.18  (27484.57-59248.97) | 261421  (209896-325273) | 42801.65  (27507.23-59649.46) | 0.02 (0.01 to 0.03) | 0.002 |
| Georgia | 647567  (528601-809062) | 46943.84  (30960.24-63013.19) | 373636  (297489-464154) | 46750.88  (31113.52-62886.2) | -0.02 (-0.04 to -0.01) | 0.006 |
| Germany | 7757161  (5945890-9745085) | 39336.96  (25291.06-56349.49) | 6030649  (4823637-7526226) | 35059.04  (23126.26-50367.54) | -0.37 (-0.39 to -0.36) | <0.001 |
| Ghana | 1352646  (1030008-1764224) | 38386.93  (23985.61-55293.08) | 3369322  (2589739-4369644) | 36840.31  (23240.52-53198.73) | -0.18 (-0.2 to -0.15) | <0.001 |
| Greece | 1279996  (1053985-1507154) | 50678.09  (36348.51-64631.05) | 1009763  (815927-1238341) | 44899.43  (30083.24-60839.16) | -0.38 (-0.39 to -0.37) | <0.001 |
| Greenland | 3916  (2985-5320) | 25722.75  (16202.29-38799.44) | 3175  (2355-4141) | 24686.46  (15346.09-37303.85) | -0.15 (-0.17 to -0.12) | <0.001 |
| Grenada | 9310  (7578-11297) | 48579.92  (32536.76-64262.04) | 11908  (9614-14561) | 46301.35  (30822.54-62648.34) | -0.16 (-0.16 to -0.15) | <0.001 |
| Guam | 15670  (12405-19752) | 44723.72  (28869.52-62514.81) | 16140  (12617-20007) | 44731.48  (28820.7-62112.33) | 0 (-0.01 to 0.02) | 0.336 |
| Guatemala | 827065  (672032-1019158) | 46163.98  (30696.72-62524.61) | 1949279  (1536786-2397968) | 44839.58  (29629.36-60631.82) | -0.09 (-0.1 to -0.09) | <0.001 |
| Guinea | 585577  (456903-734290) | 43077.03  (27929.58-59584.09) | 1419107  (1117269-1753540) | 43024.24  (28017.09-60266.05) | -0.02 (-0.05 to 0.01) | 0.116 |
| Guinea-Bissau | 118843  (97594-144233) | 51281.01  (34749.26-66873.67) | 264693  (215520-320036) | 50768.81  (34392.68-66528.88) | -0.05 (-0.09 to -0.01) | 0.012 |
| Guyana | 99388  (80615-120237) | 49351.66  (33698.68-65228.49) | 95029  (76760-116724) | 46750.76  (31726.99-62688.49) | -0.18 (-0.18 to -0.17) | <0.001 |
| Haiti | 768021  (628334-913236) | 50620.29  (34687.62-66209.76) | 1544699  (1252089-1898511) | 43776.26  (30008.33-59555.13) | -0.46 (-0.48 to -0.44) | <0.001 |
| Honduras | 479434  (388540-586757) | 46687.74  (31415.82-62921.32) | 1291077  (1043837-1571359) | 45966  (30605.73-62161.92) | -0.08 (-0.11 to -0.06) | <0.001 |
| Hungary | 1258505  (1025273-1522616) | 49714.51  (33256.76-66252.43) | 1008584  (797587-1259919) | 46921.26  (30816.48-64138.06) | -0.19 (-0.2 to -0.17) | <0.001 |
| Iceland | 29874  (24602-35638) | 46047.7  (31649.49-61274.32) | 33960  (27397-41669) | 42263.02  (28405.67-57757.33) | -0.29 (-0.31 to -0.28) | <0.001 |
| India | 81841634  (68345057-99822255) | 41024.1  (29079.04-54833.7) | 144517572  (120619717-176640550) | 38260.84  (27734.83-50968.98) | -0.22 (-0.26 to -0.19) | <0.001 |
| Indonesia | 18172137  (14841424-22405687) | 38291.77  (25837.11-53252.51) | 28037376  (22967945-34993475) | 37048.53  (25316.26-51846.23) | -0.13 (-0.17 to -0.1) | <0.001 |
| Iran (Islamic Republic of) | 4806382  (4039155-5726735) | 38590.79  (28292.77-50001.73) | 8843531  (7462553-10678148) | 37699.1  (27775.96-49179.14) | -0.07 (-0.09 to -0.06) | <0.001 |
| Iraq | 1822563  (1440907-2281903) | 45477.56  (29969.25-62569.35) | 4592194  (3645161-5778375) | 43775.84  (28653.31-60256.05) | -0.13 (-0.14 to -0.12) | <0.001 |
| Ireland | 383853  (305407-487952) | 43802.96  (28376.81-60284.36) | 476941  (369984-599812) | 39857.28  (26226.62-55745.81) | -0.31 (-0.33 to -0.3) | <0.001 |
| Israel | 573720  (469618-696580) | 47483.39  (31879.44-63556.93) | 1016005  (843823-1246493) | 45370.38  (30842.75-61004.86) | -0.16 (-0.2 to -0.13) | <0.001 |
| Italy | 5308025  (4188628-6675432) | 36932.11  (23931.5-52660.26) | 4350863  (3451944-5467171) | 34918.26  (23596.39-49239.58) | -0.18 (-0.19 to -0.16) | <0.001 |
| Jamaica | 283547  (232049-341418) | 48267.18  (32598.8-63657.38) | 366385  (296449-445536) | 47127.13  (31708.43-63411.32) | -0.09 (-0.1 to -0.08) | <0.001 |
| Japan | 6842070  (5693746-8647202) | 22130.85  (16331.01-30437.19) | 4100456  (3486178-4884529) | 18137.21  (13869.4-24113.82) | -0.63 (-0.67 to -0.61) | <0.001 |
| Jordan | 379133  (297626-473659) | 46135.18  (30294.91-63031.92) | 1374919  (1082881-1715355) | 44575.6  (28818.64-61229.02) | -0.1 (-0.11 to -0.09) | <0.001 |
| Kazakhstan | 1921928  (1527629-2367536) | 46662.17  (30887.27-63036.64) | 2163225  (1737163-2680599) | 45262.08  (29460.41-61748.42) | -0.09 (-0.1 to -0.08) | <0.001 |
| Kenya | 1762678  (1469083-2125660) | 34431.53  (24934.53-45561.11) | 4407401  (3682721-5307260) | 33533.85  (24428.97-44153.8) | -0.09 (-0.1 to -0.08) | <0.001 |
| Kiribati | 9403  (7592-11436) | 50254.7  (33865.39-66841.22) | 16188  (13234-19718) | 50702.41  (34262.12-67174.97) | 0.04 (0.02 to 0.06) | 0.000 |
| Kuwait | 175338  (139643-222545) | 42230.55  (27687.97-59308.38) | 609656  (466526-778850) | 41136.15  (26184.67-58406.6) | -0.09 (-0.1 to -0.08) | <0.001 |
| Kyrgyzstan | 502251  (398692-610393) | 48220.75  (32426.31-64563.9) | 831628  (684939-1007348) | 48099.24  (32176.19-64244.16) | 0 (-0.01 to 0) | 0.309 |
| Lao People's Democratic Republic | 391622  (311917-490686) | 40778.09  (26854.51-57125.55) | 739409  (572506-958529) | 37306.59  (24436.77-53652.12) | -0.28 (-0.29 to -0.28) | <0.001 |
| Latvia | 276911  (222807-342633) | 42346.48  (28291.12-58668.59) | 161584  (128512-203921) | 40896.63  (26652.74-57446.54) | -0.13 (-0.14 to -0.12) | <0.001 |
| Lebanon | 341722  (279043-421661) | 45737.95  (29851.05-62721.63) | 655285  (508628-811810) | 43891.56  (28617.33-60941.09) | -0.13 (-0.13 to -0.12) | <0.001 |
| Lesotho | 154332  (118405-199065) | 41226.02  (26276.91-58206.6) | 199609  (156454-253577) | 39572.8  (24551.48-56327.15) | -0.14 (-0.15 to -0.13) | <0.001 |
| Liberia | 240034  (186381-297768) | 43448.37  (27912.18-60245.48) | 609846  (490930-752653) | 44013.35  (28337.2-60472.21) | 0.03 (0.01 to 0.06) | 0.007 |
| Libya | 385199  (302420-485720) | 42895.26  (27235.57-60058.91) | 898009  (712226-1109727) | 44927.81  (29314.74-61753.75) | 0.14 (0.12 to 0.16) | <0.001 |
| Lithuania | 451799  (371119-533987) | 48905.91  (33672.14-63668.14) | 261809  (216564-322437) | 44848.55  (29549.03-60939.68) | -0.28 (-0.28 to -0.27) | <0.001 |
| Luxembourg | 41391  (32865-51579) | 41660.16  (26906.33-58138.03) | 63218  (49948-78446) | 39318.48  (25952.68-55391.39) | -0.18 (-0.19 to -0.17) | <0.001 |
| Madagascar | 1562834  (1290762-1832961) | 58186.76  (41867.38-72731.28) | 3923457  (3243679-4596033) | 55226.22  (38861.86-69825.09) | -0.17 (-0.18 to -0.16) | <0.001 |
| Malawi | 1055368  (843113-1300387) | 47651.46  (31856.82-64025.66) | 2292670  (1824438-2829182) | 46839.42  (31236.89-62894.99) | -0.07 (-0.08 to -0.05) | <0.001 |
| Malaysia | 1548483  (1179681-2043777) | 34411.98  (20994.96-51545.86) | 2642449  (2016707-3476154) | 31242.71  (18845.29-47194.57) | -0.31 (-0.33 to -0.29) | <0.001 |
| Maldives | 18981  (15368-23935) | 40331.45  (26635.56-56527.1) | 43897  (34203-55205) | 37870.62  (24614.95-53707.3) | -0.2 (-0.21 to -0.2) | <0.001 |
| Mali | 843134  (664735-1043492) | 44168.33  (28579.32-61163.54) | 2327550  (1843417-2943171) | 43167.75  (28137.15-59943.74) | -0.09 (-0.1 to -0.07) | <0.001 |
| Malta | 42870  (34052-53412) | 44343.85  (29462.4-60777.46) | 39755  (31280-49696) | 40804.78  (26710.16-57279.94) | -0.26 (-0.27 to -0.25) | <0.001 |
| Marshall Islands | 4837  (3913-5942) | 49710.53  (33078.09-66220.6) | 7326  (5817-8949) | 49516.59  (32643.39-66145.23) | -0.01 (-0.02 to 0) | 0.124 |
| Mauritania | 198919  (157348-247417) | 42633.1  (27278.98-59319.25) | 447739  (352117-560916) | 41993.57  (26869.3-58425.27) | -0.06 (-0.07 to -0.05) | <0.001 |
| Mauritius | 118725  (94182-150281) | 39744.53  (26200.54-56035.79) | 118312  (92105-149919) | 37207.92  (23706.1-53865.96) | -0.21 (-0.21 to -0.2) | <0.001 |
| Mexico | 7019007  (5660504-8956125) | 32336.94  (21799.48-46087) | 10793054  (8625327-13634433) | 30708.64  (21046.67-43684.92) | -0.16 (-0.17 to -0.15) | <0.001 |
| Micronesia (Federated States of) | 11557  (9472-14231) | 50301.05  (34475.99-66540.71) | 12938  (10529-15989) | 49679.02  (33894.76-66318.08) | -0.04 (-0.05 to -0.02) | <0.001 |
| Monaco | 2868  (2233-3665) | 39611.58  (25488.9-56117.24) | 2719  (2126-3464) | 36935  (23675.91-52736.43) | -0.23 (-0.24 to -0.22) | <0.001 |
| Mongolia | 246383  (197374-298669) | 48743.3  (32306.84-64844.52) | 393764  (314812-487411) | 46226.71  (30378.71-62405.26) | -0.19 (-0.2 to -0.17) | <0.001 |
| Montenegro | 87298  (72250-102052) | 55899.7  (39146.58-70580.76) | 76008  (64166-90336) | 52381.21  (36612.99-67745.17) | -0.22 (-0.23 to -0.21) | <0.001 |
| Morocco | 1724031  (1381618-2193843) | 26816.74  (18249.15-39827.44) | 2399223  (1897528-3019851) | 24864.62  (16938.87-36732.73) | -0.23 (-0.29 to -0.18) | <0.001 |
| Mozambique | 1923910  (1629911-2175853) | 61814.4  (47745.68-73630.53) | 3523838  (2796911-4321405) | 47322.02  (31246.95-63946.95) | -0.83 (-0.86 to -0.8) | <0.001 |
| Myanmar | 3487631  (2740003-4560550) | 33434.95  (21677.99-48513.62) | 4603064  (3572537-6017144) | 30446.8  (19701.35-45187.76) | -0.29 (-0.3 to -0.28) | <0.001 |
| Namibia | 141777  (109767-178659) | 42833.54  (26945.83-59638.64) | 276493  (214131-346050) | 41989.62  (26903.24-58188.25) | -0.06 (-0.07 to -0.05) | <0.001 |
| Nauru | 1127  (905-1385) | 46409.93  (30446.9-63196.18) | 1338  (1062-1643) | 47100.92  (30505.49-63692.82) | 0.06 (0.04 to 0.08) | <0.001 |
| Nepal | 1847902  (1456263-2378501) | 40745.45  (25714.7-57618.8) | 3373511  (2590475-4296664) | 37236.04  (23734.46-53562.94) | -0.28 (-0.3 to -0.27) | <0.001 |
| Netherlands | 1203173  (916566-1564190) | 30319.3  (19017.18-45250.93) | 1012861  (773821-1293337) | 27705.95  (17346.08-41531.57) | -0.28 (-0.3 to -0.25) | <0.001 |
| New Zealand | 287947  (227568-374521) | 31798.26  (21140.37-45993.95) | 372320  (292318-475517) | 31024.2  (20285.81-44971.93) | -0.1 (-0.12 to -0.08) | <0.001 |
| Nicaragua | 408714  (326856-503193) | 46590.91  (31289.01-62820.7) | 825683  (665674-1005669) | 45615.68  (30672.21-61695.39) | -0.06 (-0.07 to -0.05) | <0.001 |
| Niger | 804573  (649081-982396) | 46680.93  (31090.58-62795.68) | 2429134  (1963414-3016296) | 46289.97  (30637.09-63048.63) | -0.03 (-0.04 to -0.03) | <0.001 |
| Nigeria | 5821283  (4895793-7001289) | 28467.74  (20988.11-37920.33) | 15107649  (12847173-18190279) | 25921.85  (19227.95-34511.28) | -0.3 (-0.31 to -0.29) | <0.001 |
| Niue | 238  (192-295) | 48220.95  (31885.42-65136.46) | 183  (152-226) | 47692.83  (32003.33-64479.69) | -0.04 (-0.04 to -0.03) | <0.001 |
| North Macedonia | 279678  (229494-327327) | 54944.94  (38669.91-69514.72) | 274215  (226369-333071) | 51114.16  (34573.51-66479.02) | -0.23 (-0.24 to -0.22) | <0.001 |
| Northern Mariana Islands | 6211  (4864-7865) | 44707.88  (28726.67-62111.23) | 5200  (4039-6530) | 45575.1  (29719.05-62899.85) | 0.06 (0.05 to 0.08) | <0.001 |
| Norway | 391035  (323738-481752) | 36743.29  (25807.81-49968.53) | 432005  (353959-536273) | 35026.37  (24765.77-47939.42) | -0.21 (-0.33 to -0.15) | <0.001 |
| Oman | 154029  (122575-192085) | 45878.18  (29816.57-63151.79) | 452567  (361945-556243) | 44320.77  (29115.62-61427.11) | -0.12 (-0.13 to -0.11) | <0.001 |
| Pakistan | 7711544  (6204299-9766546) | 32567.3  (22005.92-46736.46) | 19837530  (15811277-24994849) | 32842.11  (22226.54-46997.83) | 0.02 (0 to 0.03) | 0.018 |
| Palau | 1941  (1548-2418) | 47391.56  (30850.97-64608.29) | 1774  (1345-2181) | 47373.94  (31091.02-64366.74) | 0 (-0.03 to 0.03) | 0.849 |
| Palestine | 180845  (140882-231658) | 41334.49  (26860.45-58593.16) | 510432  (397181-658937) | 39396.37  (24861.36-56766.47) | -0.17 (-0.18 to -0.16) | <0.001 |
| Panama | 324932  (267977-383925) | 53684.71  (37518.05-68513.09) | 539450  (439332-641662) | 50422.01  (34830.72-66276.08) | -0.2 (-0.21 to -0.19) | <0.001 |
| Papua New Guinea | 480942  (382992-596806) | 50105.83  (32986.8-67283.55) | 1286834  (1038935-1579974) | 49190.9  (33096.81-65305.26) | -0.05 (-0.06 to -0.04) | <0.001 |
| Paraguay | 465762  (378971-566272) | 49650.73  (33352.94-66048.01) | 962798  (769182-1161186) | 50679.58  (34087.97-65883.1) | 0.06 (0.05 to 0.07) | <0.001 |
| Peru | 2782086  (2280412-3352845) | 51764.41  (35708.13-67284.89) | 4897508  (4004717-5865820) | 50723.34  (34234.35-67163.36) | -0.05 (-0.07 to -0.04) | <0.001 |
| Philippines | 5990498  (5015273-7146044) | 39201.65  (28782.07-50960.2) | 11140426  (9501786-13139669) | 38186.54  (28724.09-49059.82) | -0.08 (-0.09 to -0.08) | <0.001 |
| Poland | 3873392  (3300938-4582552) | 41095.97  (30625.74-52732.83) | 3463902  (2947517-4090381) | 38726.4  (29039.22-50178.19) | -0.21 (-0.24 to -0.19) | <0.001 |
| Portugal | 949871  (746663-1212764) | 37506.97  (23406.42-54251.89) | 847475  (656429-1092416) | 35448  (22724.65-51708.45) | -0.19 (-0.21 to -0.17) | <0.001 |
| Puerto Rico | 438915  (354349-532571) | 45895.19  (30229.78-61861.57) | 335177  (272028-408088) | 44379.38  (29032.02-60560.37) | -0.12 (-0.14 to -0.1) | <0.001 |
| Qatar | 32910  (25926-42085) | 41604.88  (26632.43-59535.07) | 223999  (177996-285275) | 40013.49  (25887.07-56558.58) | -0.13 (-0.14 to -0.13) | <0.001 |
| Republic of Korea | 3851900  (3064822-4845779) | 30155.5  (20684.05-43100.25) | 3235232  (2600460-4033254) | 28459.64  (19925.36-40835) | -0.19 (-0.2 to -0.18) | <0.001 |
| Republic of Moldova | 504391  (410541-621826) | 44501.4  (29487.1-60356.73) | 397860  (311625-497428) | 44106.67  (28849.92-60777.69) | -0.04 (-0.07 to -0.02) | 0.001 |
| Romania | 3161680  (2625995-3689953) | 56273.44  (39964.11-71180.03) | 2143353  (1768913-2536288) | 52457.88  (36336.14-67884.49) | -0.23 (-0.25 to -0.22) | <0.001 |
| Russian Federation | 15207608  (12431397-18846704) | 40796.35  (27932.55-55588.52) | 13721567  (11264787-16899905) | 39591.15  (27658.72-53526.81) | -0.1 (-0.12 to -0.09) | <0.001 |
| Rwanda | 758485  (608779-931135) | 47673.67  (31622.31-63773.2) | 1577137  (1261962-1948475) | 45317.21  (30028.73-62045.49) | -0.15 (-0.16 to -0.14) | <0.001 |
| Saint Kitts and Nevis | 4685  (3727-5769) | 47457.2  (31741.95-63768.42) | 7144  (5604-8776) | 45296.47  (29817.85-61681.22) | -0.15 (-0.16 to -0.15) | <0.001 |
| Saint Lucia | 16126  (12945-19692) | 48094.74  (32514.24-64241.85) | 21209  (17291-25844) | 45919.81  (30731.37-61823.64) | -0.14 (-0.15 to -0.11) | <0.001 |
| Saint Vincent and the Grenadines | 15131  (12634-17516) | 57294.28  (40753.11-71789.98) | 14562  (12106-17097) | 52182.68  (36179.81-67149.08) | -0.31 (-0.33 to -0.3) | <0.001 |
| Samoa | 18112  (14081-22415) | 49663.41  (32701.36-66422.54) | 23647  (18794-29127) | 48867.14  (32314.71-65541.36) | -0.04 (-0.05 to -0.04) | <0.001 |
| San Marino | 2611  (2104-3254) | 41820.94  (27769.93-58323.46) | 2953  (2401-3710) | 40164.83  (26611.28-56553.08) | -0.12 (-0.14 to -0.11) | <0.001 |
| Sao Tome and Principe | 10880  (8503-13799) | 42735.89  (27528.38-60097.36) | 23637  (18566-29567) | 42433.32  (27439.41-59515.78) | -0.03 (-0.03 to -0.02) | <0.001 |
| Saudi Arabia | 1543177  (1259138-1902967) | 48093.4  (31927.67-64477.36) | 4769926  (3849751-5841851) | 46456.61  (31090.04-62710.11) | -0.13 (-0.18 to -0.11) | <0.001 |
| Senegal | 587696  (471665-733905) | 34890.5  (23080.45-49429.6) | 1333468  (1061354-1669720) | 34721.45  (22589.41-49669.71) | -0.02 (-0.02 to -0.01) | <0.001 |
| Serbia | 1269050  (1024712-1500513) | 54473.04  (38225.46-69412.65) | 1017495  (829195-1232640) | 49942.6  (33611.16-66492.1) | -0.25 (-0.36 to -0.13) | <0.001 |
| Seychelles | 7008  (5547-9004) | 38954.65  (25094.91-54941.73) | 8993  (7217-11629) | 37008.37  (24573.42-53023.89) | -0.16 (-0.17 to -0.16) | <0.001 |
| Sierra Leone | 480775  (399902-577311) | 47538.11  (33133.22-63325.46) | 923811  (752366-1131216) | 41754.66  (28606.04-57450.44) | -0.41 (-0.43 to -0.39) | <0.001 |
| Singapore | 233822  (177642-295974) | 24682.73  (16547.03-36106.05) | 328665  (250885-415977) | 23721.02  (16249.18-34021.13) | -0.13 (-0.15 to -0.11) | <0.001 |
| Slovakia | 694585  (575017-824550) | 52316.94  (35783.86-68377.85) | 619656  (505298-748416) | 48658.23  (32492.58-64943.89) | -0.23 (-0.24 to -0.22) | <0.001 |
| Slovenia | 245614  (205321-293190) | 49256.15  (34561.37-64305.89) | 198102  (157744-244176) | 46595.57  (30448.62-63043.32) | -0.17 (-0.18 to -0.17) | <0.001 |
| Solomon Islands | 37314  (29622-45590) | 50595.2  (34171.12-67154.91) | 85311  (69821-105563) | 49923.41  (33584.25-66526.3) | -0.02 (-0.05 to 0) | 0.022 |
| Somalia | 832919  (676920-1004431) | 49935.96  (33946.2-65892.87) | 2374457  (1931515-2867002) | 50107.15  (33877.82-65630.03) | 0.01 (0.01 to 0.02) | <0.001 |
| South Africa | 3508954  (2889444-4271041) | 37254.81  (26605.71-49794.34) | 5276019  (4329383-6575515) | 33921.92  (23839.75-46624.75) | -0.3 (-0.31 to -0.28) | <0.001 |
| South Sudan | 559948  (440959-698134) | 44131.46  (28311.46-60919.75) | 1048916  (825018-1308886) | 46084.32  (29994.11-63326.6) | 0.13 (0.12 to 0.14) | <0.001 |
| Spain | 4410561  (3527802-5335947) | 45849.54  (30446.95-61987.53) | 4340153  (3464723-5407793) | 42067.65  (27864.48-58348.23) | -0.27 (-0.29 to -0.26) | <0.001 |
| Sri Lanka | 1898588  (1527230-2379773) | 41473.36  (27241.02-57784.81) | 2166632  (1698891-2784335) | 38286.13  (24773.57-54210.22) | -0.26 (-0.26 to -0.25) | <0.001 |
| Sudan | 2239910  (1772713-2770831) | 48313.78  (32504.3-64186.35) | 5213022  (4180837-6388783) | 46559.33  (30875.19-63449.17) | -0.12 (-0.13 to -0.11) | <0.001 |
| Suriname | 36794  (29252-47318) | 37992.03  (24265.58-55122.42) | 52599  (40636-66959) | 36205.99  (22885.65-52256.44) | -0.13 (-0.16 to -0.11) | <0.001 |
| Sweden | 745346  (574968-971674) | 36234.59  (21931.66-53655) | 850072  (661498-1083788) | 37943.34  (24337.5-54108.96) | 0.17 (0.15 to 0.19) | <0.001 |
| Switzerland | 968729  (816304-1122911) | 53849.78  (38239.13-67870.71) | 1047958  (891712-1230645) | 51811.57  (36850.42-66142.01) | -0.12 (-0.12 to -0.11) | <0.001 |
| Syrian Arab Republic | 1280920  (1025991-1584959) | 47111.53  (31603.83-63593.01) | 1768801  (1438452-2166292) | 45756.02  (30088.36-62553.28) | -0.09 (-0.1 to -0.08) | <0.001 |
| Taiwan (Province of China) | 1081808  (779791-1519614) | 19340.85  (10890.48-32120.73) | 979394  (708475-1396331) | 18139.08  (10177.35-30105.34) | -0.17 (-0.21 to -0.13) | <0.001 |
| Tajikistan | 579255  (468446-706313) | 48737.12  (32982.36-64800.71) | 1222368  (1017502-1477478) | 48109.26  (32956.01-63730.14) | -0.04 (-0.04 to -0.04) | <0.001 |
| Thailand | 6410958  (4975832-8138666) | 40198.35  (25754.66-56740.9) | 6264513  (4991583-7803829) | 38067.5  (25091.11-53843.9) | -0.18 (-0.19 to -0.17) | <0.001 |
| Timor-Leste | 88520  (71519-106830) | 47810.78  (32207.46-63668.38) | 154160  (122950-189175) | 45116.72  (29970.58-61241.85) | -0.21 (-0.27 to -0.17) | <0.001 |
| Togo | 371898  (297931-458298) | 43916.12  (28067.81-60787.78) | 775987  (624759-966130) | 36332.06  (24196.69-51062.94) | -0.59 (-0.61 to -0.56) | <0.001 |
| Tokelau | 175  (143-213) | 49896.88  (33079.14-66703.46) | 155  (123-189) | 48237.86  (31386.89-65328.73) | -0.11 (-0.12 to -0.1) | <0.001 |
| Tonga | 10923  (8706-13583) | 49245.02  (32240.6-66243.19) | 12141  (10060-14974) | 48239.32  (31705.34-64996.62) | -0.05 (-0.06 to -0.03) | <0.001 |
| Trinidad and Tobago | 144759  (117163-177705) | 47167.42  (31587.48-63232.87) | 124446  (100211-155172) | 36421.65  (24362.69-52381.13) | -0.8 (-0.82 to -0.78) | <0.001 |
| Tunisia | 919256  (725561-1147848) | 45270.66  (29568.88-62314.11) | 1347524  (1065554-1667802) | 43596.55  (28667.99-60490.01) | -0.12 (-0.13 to -0.11) | <0.001 |
| Turkey | 6852469  (5519474-8384559) | 48546.8  (32956.42-64563.48) | 9955347  (8062476-12213189) | 45705.01  (29769.39-62233.43) | -0.19 (-0.21 to -0.16) | <0.001 |
| Turkmenistan | 409532  (329565-509419) | 46677.33  (31212.8-62902.88) | 571361  (458434-705688) | 45456.39  (30220.19-62033.01) | -0.11 (-0.13 to -0.1) | <0.001 |
| Tuvalu | 1241  (1019-1505) | 50946.19  (34940.63-67267.53) | 1429  (1134-1739) | 49308.67  (32725.68-65983.1) | -0.1 (-0.13 to -0.06) | <0.001 |
| Uganda | 1276829  (991301-1696034) | 33640.54  (21082.45-49656.34) | 3342149  (2482298-4446200) | 32446.33  (19527.44-49121.64) | -0.14 (-0.16 to -0.1) | <0.001 |
| Ukraine | 4719919  (3697178-5994106) | 36987.22  (24431.18-52528.89) | 3819847  (2950474-4880630) | 37023.28  (24216.22-52754.99) | 0.01 (0 to 0.02) | 0.076 |
| United Arab Emirates | 139581  (109190-178548) | 40747.92  (26184.04-57844.1) | 716368  (547055-898078) | 40872.91  (26686.21-57889.59) | -0.03 (-0.06 to 0) | 0.025 |
| United Kingdom | 5634687  (4821938-6698267) | 39248.96  (28895.61-50664.18) | 5698412  (4887422-6708724) | 36486.53  (27382.39-46448.86) | -0.24 (-0.24 to -0.23) | <0.001 |
| United Republic of Tanzania | 2597725  (2035271-3225473) | 44105.7  (28517.89-59477.39) | 6311821  (4988131-7908569) | 42866.01  (27002.05-59572.19) | -0.09 (-0.1 to -0.08) | <0.001 |
| United States of America | 18211066  (14327338-22923716) | 27207.23  (18593.96-39292.67) | 19718387  (16021386-24351325) | 26176.87  (18797.43-36466.68) | -0.12 (-0.13 to -0.11) | <0.001 |
| United States Virgin Islands | 13300  (10852-16320) | 46346.33  (31072.84-62507.24) | 7638  (6218-9397) | 44075.18  (29535.09-60001.7) | -0.15 (-0.16 to -0.14) | <0.001 |
| Uruguay | 367565  (297166-449340) | 48959.69  (32448.48-65491.81) | 398818  (325865-488631) | 47363.71  (31776.61-63928.82) | -0.1 (-0.11 to -0.1) | <0.001 |
| Uzbekistan | 2366094  (1917970-2891135) | 48799.28  (32747.98-64800.04) | 4197091  (3446980-5164740) | 46809.8  (31383.47-63140.36) | -0.13 (-0.14 to -0.13) | <0.001 |
| Vanuatu | 17530  (14148-21550) | 50174.31  (33347.89-66758.93) | 39385  (31852-48092) | 50343.76  (33887.83-66804.15) | 0.01 (0.01 to 0.02) | <0.001 |
| Venezuela (Bolivarian Republic of) | 2231218  (1827040-2743697) | 47047.02  (31686.99-63116.01) | 3265160  (2664203-3917323) | 47009.06  (32001.52-62789.13) | -0.02 (-0.05 to 0) | 0.057 |
| Viet Nam | 6678544  (5306963-8394210) | 39416.15  (25861.52-55764.28) | 9020231  (6987002-11669324) | 35207.07  (22552.64-51323.06) | -0.4 (-0.45 to -0.35) | <0.001 |
| Yemen | 1221941  (973737-1534977) | 44884.01  (28854.1-61394.41) | 3670960  (2940808-4533008) | 44289.26  (28694.92-60626.51) | -0.04 (-0.05 to -0.03) | <0.001 |
| Zambia | 774714  (640240-927412) | 42233.43  (29243.27-57737.96) | 1814929  (1438892-2278260) | 37587.29  (24970.9-53289.41) | -0.36 (-0.37 to -0.35) | <0.001 |
| Zimbabwe | 795110  (601010-1016053) | 33374.29  (20532.59-49358.25) | 1354825  (1025261-1806311) | 33488.94  (20065.46-50112.82) | -0.01 (-0.05 to 0.02) | 0.550 |

AAPC: average annual percent change, ASR: age-standardized rate, WCBA: women of childbearing age.

Supplementary Table 4**.** The case number and ASR of DALYs of caries among WCBA in 1990 and 2021 in 204 countries and territories, with AAPC from 1990 to 2021

| **Location** | **1990** | | **2021** | | **AAPC (95%CI)**  **1990–2021** | ***P*** |
| --- | --- | --- | --- | --- | --- | --- |
|  | **Number(95%UI)** | **ASR (95%UI)** | **Number (95%UI)** | **ASR (95%UI)** |  |  |
| Afghanistan | 1021  (425-1999) | 47.01  (18.63-94.19) | 3304  (1387-6164) | 46.42  (18.56-91.6) | -0.04 (-0.05 to -0.03) | <0.001 |
| Albania | 448  (197-841) | 53.63  (22.34-104.88) | 307  (133-593) | 49.72  (20.09-98.49) | -0.24 (-0.25 to -0.23) | <0.001 |
| Algeria | 2544  (1065-5000) | 44.04  (17.51-89.41) | 4926  (1980-9640) | 43.7  (16.98-88.93) | -0.04 (-0.06 to -0.03) | <0.001 |
| American Samoa | 6  (2-11) | 45.89  (17.6-93.34) | 5  (2-11) | 46.64  (18.21-94.33) | 0.05 (0.04 to 0.06) | <0.001 |
| Andorra | 6  (3-12) | 40.7  (15.88-82.5) | 8  (3-16) | 38.6  (15.31-78.11) | -0.18 (-0.19 to -0.17) | <0.001 |
| Angola | 921  (379-1854) | 40.51  (15.68-83.15) | 2957  (1180-5793) | 39.05  (14.71-80.43) | -0.12 (-0.13 to -0.12) | <0.001 |
| Antigua and Barbuda | 8  (3-14) | 46.5  (18.69-92.29) | 11  (5-21) | 44.93  (18.02-89.94) | -0.12 (-0.13 to -0.11) | <0.001 |
| Argentina | 3843  (1634-7304) | 48.1  (19.59-94.66) | 5705  (2441-10932) | 47.56  (19.23-94.14) | -0.05 (-0.08 to -0.03) | <0.001 |
| Armenia | 412  (175-796) | 47.4  (18.99-95.81) | 344  (144-658) | 46.08  (18.56-91.54) | -0.09 (-0.1 to -0.08) | <0.001 |
| Australia | 1837  (774-3528) | 41.02  (16.38-81.06) | 2100  (859-4018) | 34.52  (13.13-69.83) | -0.56 (-0.59 to -0.53) | <0.001 |
| Austria | 631  (259-1210) | 31.52  (11.84-65) | 596  (253-1165) | 29.77  (11.49-61.33) | -0.22 (-0.27 to -0.18) | <0.001 |
| Azerbaijan | 870  (371-1703) | 46.35  (18.75-92.37) | 1258  (546-2397) | 45.35  (18.21-90.59) | -0.08 (-0.11 to -0.06) | <0.001 |
| Bahamas | 36  (16-68) | 49.21  (19.89-96.8) | 52  (23-100) | 47.98  (19.83-96.92) | -0.08 (-0.09 to -0.07) | <0.001 |
| Bahrain | 50  (21-97) | 43.02  (16.81-88.62) | 135  (55-267) | 41.18  (16-82.92) | -0.14 (-0.15 to -0.14) | <0.001 |
| Bangladesh | 9892  (3824-18981) | 40.41  (15.4-81.51) | 17907  (7396-34640) | 38.94  (14.95-78.51) | -0.12 (-0.13 to -0.11) | <0.001 |
| Barbados | 32  (13-60) | 46.14  (18.65-92.96) | 33  (14-63) | 45.81  (18.58-91.39) | -0.02 (-0.03 to -0.02) | <0.001 |
| Belarus | 1082  (461-2081) | 42.34  (16.61-84.82) | 898  (384-1723) | 41.69  (16.41-83.5) | -0.07 (-0.09 to -0.05) | <0.001 |
| Belgium | 848  (355-1694) | 34.47  (13.27-71.39) | 821  (346-1630) | 32.65  (12.78-67.34) | -0.2 (-0.22 to -0.18) | <0.001 |
| Belize | 20  (9-39) | 48.44  (19.63-97.41) | 48  (21-90) | 39.86  (16.21-82.58) | -0.62 (-0.63 to -0.6) | <0.001 |
| Benin | 470  (192-902) | 42.93  (16.75-85.9) | 1352  (574-2672) | 41.86  (16.42-84.98) | -0.1 (-0.13 to -0.07) | <0.001 |
| Bermuda | 8  (3-15) | 44.81  (18.02-90.57) | 6  (2-11) | 42.92  (17.03-86.6) | -0.16 (-0.18 to -0.14) | <0.001 |
| Bhutan | 57  (23-109) | 39.89  (15.09-81.68) | 77  (31-150) | 37.38  (14.14-75.43) | -0.19 (-0.22 to -0.17) | <0.001 |
| Bolivia (Plurinational State of) | 789  (328-1527) | 51.98  (21.1-104.11) | 1575  (691-3075) | 50.55  (20.65-100.62) | -0.04 (-0.07 to -0.01) | 0.022 |
| Bosnia and Herzegovina | 650  (284-1241) | 55.74  (23.33-108.52) | 365  (153-702) | 49.61  (20.14-99.04) | -0.38 (-0.39 to -0.37) | <0.001 |
| Botswana | 120  (49-228) | 37.74  (14.37-79.04) | 242  (100-451) | 35.42  (13.61-72.17) | -0.21 (-0.21 to -0.2) | <0.001 |
| Brazil | 14817  (6136-28718) | 38.18  (15.08-76.13) | 22223  (9340-42962) | 37.55  (14.95-75) | -0.06 (-0.08 to -0.03) | 0.000 |
| Brunei Darussalam | 16  (7-31) | 23.18  (9.05-46.75) | 29  (12-57) | 23.62  (9.13-48.09) | 0.06 (0.05 to 0.07) | <0.001 |
| Bulgaria | 1106  (482-2091) | 53.32  (22-106.26) | 730  (311-1391) | 50.72  (20.81-102.37) | -0.15 (-0.16 to -0.14) | <0.001 |
| Burkina Faso | 882  (374-1688) | 42.36  (16.76-85.08) | 2269  (947-4458) | 41.55  (15.97-84.34) | -0.1 (-0.13 to -0.07) | <0.001 |
| Burundi | 577  (240-1116) | 46.38  (18.29-92.22) | 1440  (609-2718) | 46.85  (18.82-92.1) | 0.03 (0.02 to 0.03) | <0.001 |
| Cabo Verde | 34  (14-66) | 42.77  (16.9-86.46) | 61  (25-121) | 40.43  (15.66-84.28) | -0.19 (-0.21 to -0.18) | <0.001 |
| Cambodia | 1129  (478-2247) | 45.57  (18.19-92.48) | 1967  (842-3800) | 43.61  (17.43-89.03) | -0.14 (-0.15 to -0.14) | <0.001 |
| Cameroon | 974  (405-1899) | 41.03  (15.85-82.86) | 3249  (1324-6041) | 41.74  (16.31-84.01) | 0.01 (-0.02 to 0.04) | 0.612 |
| Canada | 1936  (812-3778) | 25.96  (9.76-53.43) | 2065  (860-3966) | 24.78  (9.5-51.57) | -0.15 (-0.17 to -0.13) | <0.001 |
| Central African Republic | 268  (112-532) | 42.35  (16.64-86.3) | 582  (246-1135) | 42.52  (16.7-86.76) | 0.03 (0.02 to 0.04) | <0.001 |
| Chad | 570  (241-1089) | 43.04  (17.03-86.44) | 1611  (687-3117) | 42.02  (16.65-85.59) | -0.08 (-0.09 to -0.07) | <0.001 |
| Chile | 1744  (735-3343) | 48.65  (19.74-96.62) | 2313  (1082-4382) | 48.14  (22.24-91.22) | -0.03 (-0.09 to 0.03) | 0.262 |
| China | 90343  (36221-172989) | 27.17  (10.29-56.03) | 87212  (36535-170421) | 28.09  (11.02-57.66) | 0.13 (0.1 to 0.15) | <0.001 |
| Colombia | 3649  (1535-6897) | 42.32  (16.41-85.62) | 5330  (2235-10291) | 40.47  (15.89-82.98) | -0.34 (-0.7 to -0.14) | 0.002 |
| Comoros | 41  (18-78) | 39.55  (15.72-81.59) | 76  (34-149) | 39.44  (15.38-82.29) | -0.01 (-0.02 to 0) | 0.078 |
| Congo | 220  (92-431) | 39.59  (15.59-80.94) | 558  (228-1063) | 39.17  (15.06-81.22) | -0.03 (-0.04 to -0.03) | <0.001 |
| Cook Islands | 2  (1-4) | 46.47  (17.97-93.09) | 2  (1-4) | 45.25  (17.15-91.86) | -0.09 (-0.1 to -0.08) | <0.001 |
| Costa Rica | 344  (143-655) | 44.69  (17.65-89.42) | 560  (236-1085) | 42.86  (16.94-86.29) | -0.13 (-0.14 to -0.12) | <0.001 |
| Coted'Ivoire | 1125  (475-2112) | 41.14  (16.24-84.57) | 2719  (1112-5249) | 40.94  (15.89-82.88) | -0.02 (-0.02 to -0.01) | 0.000 |
| Croatia | 670  (289-1288) | 55.87  (23.11-109.57) | 468  (201-902) | 51.77  (21.12-102.09) | -0.24 (-0.25 to -0.23) | <0.001 |
| Cuba | 1459  (618-2768) | 47.82  (19.52-93.99) | 1162  (506-2242) | 46.09  (18.75-91.26) | -0.1 (-0.13 to -0.08) | <0.001 |
| Cyprus | 85  (36-161) | 42.71  (17.13-84.95) | 149  (63-292) | 40.07  (15.68-81.28) | -0.19 (-0.2 to -0.18) | <0.001 |
| Czechia | 1308  (553-2458) | 50.96  (20.78-102.11) | 1120  (473-2189) | 48.61  (19.72-97.17) | -0.15 (-0.16 to -0.13) | <0.001 |
| Democratic People's Republic of Korea | 1019  (384-1958) | 17.55  (6.01-38.69) | 1239  (486-2477) | 19.06  (6.68-41.84) | 0.29 (0.25 to 0.33) | <0.001 |
| Democratic Republic of the Congo | 3461  (1447-6560) | 41.08  (16.25-82.56) | 8880  (3674-17234) | 42.24  (16.56-86.86) | 0.08 (0.06 to 0.1) | <0.001 |
| Denmark | 318  (131-624) | 24.01  (8.98-50.38) | 342  (148-667) | 27.2  (10.42-57.95) | 0.39 (0.36 to 0.42) | <0.001 |
| Djibouti | 38  (17-71) | 39.06  (15.56-81.35) | 123  (55-236) | 38.26  (15.25-79.37) | -0.07 (-0.08 to -0.06) | <0.001 |
| Dominica | 8  (3-15) | 47.72  (19.29-95.34) | 8  (3-15) | 45.95  (18.57-92.74) | -0.11 (-0.12 to -0.1) | <0.001 |
| Dominican Republic | 894  (379-1689) | 48.01  (19.42-95.23) | 1307  (555-2554) | 45.04  (18.08-89.44) | -0.21 (-0.22 to -0.21) | <0.001 |
| Ecuador | 1272  (530-2441) | 50.86  (20.4-101.22) | 2347  (987-4453) | 49.75  (20.03-98.53) | -0.06 (-0.08 to -0.04) | <0.001 |
| Egypt | 5208  (2218-10175) | 39.86  (15.37-81.65) | 9870  (4126-19405) | 38.03  (14.89-78.45) | -0.17 (-0.2 to -0.13) | <0.001 |
| El Salvador | 587  (249-1141) | 45.86  (18.58-91.63) | 790  (328-1562) | 44.43  (17.67-89.07) | -0.14 (-0.15 to -0.12) | <0.001 |
| Equatorial Guinea | 40  (17-78) | 41.31  (16.4-83.23) | 131  (53-253) | 36.16  (13.69-72.74) | -0.43 (-0.44 to -0.42) | <0.001 |
| Eritrea | 391  (171-755) | 50.3  (20.48-98.38) | 830  (357-1577) | 50.54  (20.41-99.59) | -0.01 (-0.04 to 0.01) | 0.245 |
| Estonia | 119  (48-235) | 31.19  (11.63-64.91) | 81  (33-153) | 29.21  (10.79-60.94) | -0.2 (-0.22 to -0.19) | <0.001 |
| Eswatini | 74  (30-144) | 38.58  (14.77-79.64) | 116  (47-224) | 36.74  (14.2-75.32) | -0.16 (-0.17 to -0.15) | <0.001 |
| Ethiopia | 4238  (1917-8253) | 38.48  (16.37-75.45) | 9753  (4442-18968) | 35.92  (15.17-71.02) | -0.21 (-0.22 to -0.2) | <0.001 |
| Fiji | 94  (39-182) | 48.32  (19.04-96.64) | 108  (45-202) | 47.14  (18.62-94.34) | -0.06 (-0.08 to -0.05) | <0.001 |
| Finland | 370  (155-720) | 28.93  (10.58-60.31) | 305  (127-600) | 26.63  (9.85-56.19) | -0.27 (-0.33 to -0.22) | <0.001 |
| France | 7066  (3002-13435) | 48.56  (20.11-95.24) | 6505  (2773-12396) | 44.84  (18.31-89.11) | -0.26 (-0.28 to -0.24) | <0.001 |
| Gabon | 82  (33-158) | 37.29  (14.1-77.18) | 180  (73-347) | 37.02  (14.38-76.42) | -0.03 (-0.04 to -0.02) | <0.001 |
| Gambia | 95  (40-182) | 41.7  (16.26-86.43) | 257  (108-492) | 41.91  (16.49-84.96) | 0.01 (-0.01 to 0.02) | 0.199 |
| Georgia | 642  (280-1247) | 46.55  (18.72-93.33) | 369  (153-711) | 46.3  (18.48-91.8) | -0.03 (-0.05 to -0.02) | <0.001 |
| Germany | 7630  (3112-14939) | 38.73  (15.08-79.78) | 5933  (2626-11231) | 34.53  (13.75-71.94) | -0.37 (-0.39 to -0.35) | <0.001 |
| Ghana | 1328  (563-2543) | 37.6  (14.32-79.06) | 3312  (1350-6487) | 36.18  (14.15-75.24) | -0.17 (-0.19 to -0.14) | <0.001 |
| Greece | 1260  (543-2402) | 49.91  (20.46-97.04) | 992  (406-1973) | 44.21  (17.23-88.68) | -0.38 (-0.39 to -0.37) | <0.001 |
| Greenland | 4  (2-7) | 25.22  (9.53-51.59) | 3  (1-6) | 24.27  (9.2-50.74) | -0.14 (-0.15 to -0.1) | <0.001 |
| Grenada | 9  (4-18) | 47.84  (19.49-95.06) | 12  (5-23) | 45.55  (18.41-91.18) | -0.16 (-0.16 to -0.15) | <0.001 |
| Guam | 16  (7-30) | 44.33  (17.09-89.45) | 16  (6-31) | 44.3  (17.14-90.5) | 0.01 (-0.01 to 0.02) | 0.300 |
| Guatemala | 812  (344-1526) | 45.22  (18.02-90.47) | 1918  (789-3723) | 44.06  (17.36-89.57) | -0.08 (-0.09 to -0.08) | <0.001 |
| Guinea | 576  (241-1125) | 42.29  (16.76-85.93) | 1396  (569-2620) | 42.22  (16.42-85.15) | -0.01 (-0.04 to 0.03) | 0.268 |
| Guinea-Bissau | 117  (50-225) | 50.26  (20.38-99.89) | 260  (109-505) | 49.81  (19.92-99.17) | -0.05 (-0.08 to -0.01) | 0.021 |
| Guyana | 98  (42-187) | 48.44  (19.72-97.61) | 93  (39-179) | 45.8  (18.51-91.42) | -0.18 (-0.19 to -0.17) | <0.001 |
| Haiti | 751  (322-1448) | 49.41  (20.4-97.67) | 1510  (676-2824) | 42.77  (17.27-88.16) | -0.46 (-0.48 to -0.45) | <0.001 |
| Honduras | 474  (208-906) | 46.04  (18.94-92.78) | 1274  (543-2469) | 45.3  (18.14-90.81) | -0.07 (-0.09 to -0.04) | <0.001 |
| Hungary | 1243  (525-2399) | 49.16  (19.69-96.47) | 998  (417-1948) | 46.53  (18.66-94.98) | -0.18 (-0.2 to -0.16) | <0.001 |
| Iceland | 30  (13-57) | 45.56  (18.51-91.81) | 34  (15-64) | 41.74  (16.73-84.79) | -0.29 (-0.31 to -0.28) | <0.001 |
| India | 80104  (33579-153297) | 40.09  (16.11-79.23) | 141745  (59968-269166) | 37.51  (15.24-73.89) | -0.21 (-0.25 to -0.18) | <0.001 |
| Indonesia | 18022  (7648-34628) | 37.92  (15.09-76.2) | 27778  (11640-53876) | 36.72  (14.51-73.15) | -0.12 (-0.15 to -0.08) | <0.001 |
| Iran (Islamic Republic of) | 4732  (2009-9199) | 37.88  (15.5-74.76) | 8660  (3770-16772) | 37  (15.2-73.91) | -0.08 (-0.09 to -0.07) | <0.001 |
| Iraq | 1797  (758-3522) | 44.67  (17.57-90.05) | 4505  (1842-8619) | 42.89  (16.72-85.86) | -0.14 (-0.15 to -0.13) | <0.001 |
| Ireland | 379  (161-741) | 43.18  (16.85-88.22) | 467  (203-895) | 39.06  (15.49-79.7) | -0.34 (-0.36 to -0.33) | <0.001 |
| Israel | 566  (240-1096) | 46.84  (18.75-94.76) | 1001  (429-1923) | 44.73  (18.27-89.56) | -0.16 (-0.2 to -0.13) | <0.001 |
| Italy | 5224  (2175-10089) | 36.36  (14.02-73.8) | 4280  (1802-8372) | 34.41  (13.53-69.17) | -0.17 (-0.19 to -0.14) | <0.001 |
| Jamaica | 280  (118-537) | 47.65  (19.16-94.98) | 361  (154-686) | 46.44  (18.5-93.39) | -0.09 (-0.11 to -0.08) | <0.001 |
| Japan | 6793  (2884-13275) | 21.99  (8.92-44.19) | 4072  (1786-8155) | 18.04  (7.47-35.9) | -0.63 (-0.66 to -0.6) | <0.001 |
| Jordan | 374  (155-714) | 45.36  (18.21-91.35) | 1352  (567-2573) | 43.78  (17.18-88.93) | -0.1 (-0.11 to -0.1) | <0.001 |
| Kazakhstan | 1902  (839-3638) | 46.15  (18.82-93) | 2136  (898-4157) | 44.74  (17.77-90.4) | -0.09 (-0.1 to -0.08) | <0.001 |
| Kenya | 1741  (743-3390) | 33.89  (13.9-67.04) | 4347  (1879-8440) | 33  (13.66-64.94) | -0.09 (-0.1 to -0.09) | <0.001 |
| Kiribati | 9  (4-18) | 49.5  (19.63-98.19) | 16  (7-30) | 49.97  (20-98.29) | 0.04 (0.03 to 0.06) | <0.001 |
| Kuwait | 173  (73-334) | 41.58  (16.7-85.38) | 599  (241-1172) | 40.56  (15.46-83.93) | -0.08 (-0.09 to -0.07) | <0.001 |
| Kyrgyzstan | 498  (212-970) | 47.71  (19.41-95.34) | 824  (360-1560) | 47.66  (19.27-95.3) | 0 (-0.01 to 0.01) | 0.856 |
| Lao People's Democratic Republic | 388  (159-746) | 40.28  (16.01-81.72) | 733  (295-1452) | 36.96  (14.2-75.85) | -0.27 (-0.28 to -0.26) | <0.001 |
| Latvia | 274  (112-528) | 41.9  (16.36-85.51) | 159  (68-311) | 40.47  (15.86-82.38) | -0.13 (-0.14 to -0.11) | <0.001 |
| Lebanon | 336  (141-662) | 44.9  (17.99-90.9) | 641  (263-1248) | 43.05  (16.97-86.93) | -0.13 (-0.14 to -0.12) | <0.001 |
| Lesotho | 152  (65-292) | 40.49  (15.89-83.23) | 195  (81-375) | 38.48  (14.82-78.36) | -0.17 (-0.19 to -0.16) | <0.001 |
| Liberia | 233  (95-443) | 42.02  (16.12-84.76) | 593  (255-1129) | 42.71  (17.01-85.84) | 0.05 (0.02 to 0.08) | 0.002 |
| Libya | 380  (163-723) | 42.2  (15.96-86.31) | 879  (369-1681) | 44.02  (17.4-87.54) | 0.12 (0.1 to 0.13) | <0.001 |
| Lithuania | 447  (186-876) | 48.37  (19.52-96.25) | 258  (109-516) | 44.32  (17.5-89.89) | -0.28 (-0.29 to -0.27) | <0.001 |
| Luxembourg | 41  (17-78) | 41.01  (16.06-82.16) | 62  (26-121) | 38.73  (15.45-76.92) | -0.18 (-0.19 to -0.17) | <0.001 |
| Madagascar | 1538  (658-2938) | 57.1  (23.68-111.16) | 3863  (1673-7443) | 54.27  (22.29-107.04) | -0.17 (-0.17 to -0.16) | <0.001 |
| Malawi | 1031  (425-1995) | 46.38  (18.69-93.18) | 2257  (952-4318) | 45.94  (18.22-91.37) | -0.04 (-0.05 to -0.03) | <0.001 |
| Malaysia | 1532  (610-3030) | 34.02  (12.68-70.72) | 2618  (1050-5260) | 30.95  (11.25-65.53) | -0.3 (-0.32 to -0.29) | <0.001 |
| Maldives | 19  (8-37) | 39.76  (15.5-81.75) | 43  (18-83) | 37.4  (14.55-75.15) | -0.2 (-0.21 to -0.19) | <0.001 |
| Mali | 827  (344-1597) | 43.22  (16.88-87.06) | 2291  (943-4362) | 42.35  (16.36-85.11) | -0.08 (-0.09 to -0.06) | <0.001 |
| Malta | 42  (18-83) | 43.75  (17.36-88.23) | 39  (17-75) | 40.17  (15.86-81) | -0.27 (-0.28 to -0.26) | <0.001 |
| Marshall Islands | 5  (2-9) | 49.06  (19.34-97.32) | 7  (3-14) | 48.81  (19.02-96.01) | -0.01 (-0.02 to 0) | 0.030 |
| Mauritania | 196  (82-374) | 41.93  (16.41-85.38) | 442  (185-862) | 41.4  (16.12-85.6) | -0.05 (-0.06 to -0.05) | <0.001 |
| Mauritius | 117  (48-224) | 39.27  (15.2-79.86) | 117  (49-227) | 36.71  (13.98-74.65) | -0.21 (-0.22 to -0.2) | <0.001 |
| Mexico | 6945  (2873-13446) | 31.92  (12.42-64.39) | 10630  (4503-20364) | 30.26  (11.98-60.53) | -0.17 (-0.17 to -0.17) | <0.001 |
| Micronesia (Federated States of) | 11  (5-22) | 49.71  (19.77-98.96) | 13  (5-25) | 49.04  (19.38-97.69) | -0.04 (-0.06 to -0.03) | <0.001 |
| Monaco | 3  (1-5) | 39.11  (15.19-78.83) | 3  (1-5) | 36.35  (14.32-74.79) | -0.24 (-0.25 to -0.23) | <0.001 |
| Mongolia | 244  (101-477) | 48.2  (19.28-96.46) | 389  (167-753) | 45.74  (18.26-92.51) | -0.18 (-0.2 to -0.16) | <0.001 |
| Montenegro | 87  (37-165) | 55.45  (22.91-108.98) | 75  (32-143) | 51.91  (21.07-102.8) | -0.22 (-0.23 to -0.21) | <0.001 |
| Morocco | 1698  (751-3292) | 26.35  (10.67-56.01) | 2353  (1067-4488) | 24.4  (9.95-51.3) | -0.24 (-0.29 to -0.19) | <0.001 |
| Mozambique | 1877  (796-3581) | 60.2  (25.7-116.05) | 3430  (1424-6485) | 45.9  (18.11-91.4) | -0.83 (-0.87 to -0.78) | <0.001 |
| Myanmar | 3454  (1382-6787) | 33.06  (12.48-68.8) | 4562  (1834-8783) | 30.17  (11.28-61.99) | -0.29 (-0.3 to -0.27) | <0.001 |
| Namibia | 140  (59-272) | 42.09  (16.15-85.41) | 272  (115-532) | 41.23  (16.09-83.16) | -0.05 (-0.07 to -0.04) | <0.001 |
| Nauru | 1  (0-2) | 45.91  (17.86-92.33) | 1  (1-3) | 46.53  (18.43-95.04) | 0.05 (0.03 to 0.07) | <0.001 |
| Nepal | 1810  (721-3462) | 39.84  (15.3-82.72) | 3316  (1346-6240) | 36.56  (14.2-75.19) | -0.27 (-0.29 to -0.25) | <0.001 |
| Netherlands | 1194  (505-2372) | 30.1  (11.6-64.17) | 1002  (426-1915) | 27.43  (10.59-57.91) | -0.29 (-0.31 to -0.26) | <0.001 |
| New Zealand | 283  (115-547) | 31.2  (12.05-63.09) | 366  (153-724) | 30.51  (11.9-62.3) | -0.1 (-0.12 to -0.07) | <0.001 |
| Nicaragua | 404  (160-770) | 45.97  (18.41-93.64) | 815  (362-1575) | 45  (18.23-89.59) | -0.06 (-0.07 to -0.05) | <0.001 |
| Niger | 791  (335-1520) | 45.76  (18.23-92.6) | 2397  (1020-4579) | 45.52  (17.87-91.56) | -0.02 (-0.03 to -0.01) | <0.001 |
| Nigeria | 5716  (2472-11078) | 27.86  (11.38-56.2) | 14894  (6474-29344) | 25.49  (10.65-51.39) | -0.3 (-0.31 to -0.29) | <0.001 |
| Niue | 0  (0-0) | 47.77  (18.8-95.9) | 0  (0-0) | 47.11  (18.38-93.83) | -0.04 (-0.05 to -0.04) | <0.001 |
| North Macedonia | 277  (119-516) | 54.43  (22.73-107.08) | 271  (113-520) | 50.6  (20.22-100.5) | -0.23 (-0.24 to -0.21) | <0.001 |
| Northern Mariana Islands | 6  (3-12) | 44.32  (17.39-90.08) | 5  (2-10) | 45.14  (17.56-91.17) | 0.06 (0.05 to 0.07) | <0.001 |
| Norway | 385  (163-742) | 36.23  (14.44-72.49) | 426  (181-829) | 34.54  (13.89-69.24) | -0.21 (-0.33 to -0.15) | <0.001 |
| Oman | 152  (65-292) | 45.1  (17.59-92.04) | 445  (191-854) | 43.62  (17.05-88.8) | -0.11 (-0.13 to -0.1) | <0.001 |
| Pakistan | 7594  (3194-14527) | 32  (12.53-63.97) | 19506  (7904-37742) | 32.25  (12.42-64.51) | 0.02 (0 to 0.03) | 0.017 |
| Palau | 2  (1-4) | 46.86  (18.41-92.73) | 2  (1-3) | 46.85  (18.18-93) | 0 (-0.03 to 0.03) | 0.723 |
| Palestine | 179  (72-346) | 40.71  (15.64-84.83) | 503  (204-995) | 38.74  (15.03-80.57) | -0.16 (-0.18 to -0.15) | <0.001 |
| Panama | 321  (138-611) | 52.96  (22.05-103.38) | 532  (228-1036) | 49.75  (20.1-98.05) | -0.2 (-0.21 to -0.19) | <0.001 |
| Papua New Guinea | 474  (196-909) | 49.31  (19.54-97.33) | 1270  (558-2417) | 48.52  (19.02-97.27) | -0.05 (-0.06 to -0.04) | <0.001 |
| Paraguay | 459  (190-890) | 48.89  (19.72-97.2) | 946  (387-1820) | 49.78  (19.72-98.55) | 0.05 (0.03 to 0.06) | <0.001 |
| Peru | 2750  (1189-5156) | 51.07  (20.79-99.82) | 4836  (2105-9143) | 50.11  (20.32-99.52) | -0.05 (-0.07 to -0.04) | <0.001 |
| Philippines | 5926  (2521-11491) | 38.71  (15.95-77.11) | 11017  (4802-20984) | 37.74  (15.7-74.54) | -0.08 (-0.09 to -0.07) | <0.001 |
| Poland | 3830  (1658-7390) | 40.66  (17-80.62) | 3426  (1482-6601) | 38.38  (15.84-76.33) | -0.2 (-0.23 to -0.18) | <0.001 |
| Portugal | 934  (398-1781) | 36.9  (14.33-75.23) | 832  (342-1654) | 34.86  (13.33-71.83) | -0.19 (-0.21 to -0.17) | <0.001 |
| Puerto Rico | 434  (186-837) | 45.37  (18.14-90.41) | 330  (141-624) | 43.77  (17.27-88.96) | -0.12 (-0.14 to -0.11) | <0.001 |
| Qatar | 32  (14-62) | 40.94  (15.89-84.2) | 219  (94-429) | 39.28  (15.08-81.24) | -0.14 (-0.15 to -0.13) | <0.001 |
| Republic of Korea | 3830  (1611-7504) | 29.97  (11.6-60.37) | 3215  (1359-6185) | 28.32  (11.24-58.08) | -0.19 (-0.2 to -0.17) | <0.001 |
| Republic of Moldova | 498  (209-973) | 43.99  (17.58-89.53) | 393  (165-750) | 43.67  (17-87.32) | -0.05 (-0.07 to -0.03) | <0.001 |
| Romania | 3129  (1315-6051) | 55.7  (22.9-108.2) | 2117  (934-3924) | 51.95  (21.4-102.77) | -0.23 (-0.24 to -0.22) | <0.001 |
| Russian Federation | 15006  (6351-28881) | 40.28  (16.34-80.78) | 13502  (5768-26049) | 39.07  (15.77-77.9) | -0.11 (-0.13 to -0.09) | <0.001 |
| Rwanda | 747  (311-1473) | 46.86  (18.53-95.34) | 1554  (646-2999) | 44.57  (17.65-89.61) | -0.15 (-0.16 to -0.15) | <0.001 |
| Saint Kitts and Nevis | 5  (2-9) | 46.64  (18.78-94.12) | 7  (3-14) | 44.61  (17.91-88.47) | -0.15 (-0.15 to -0.15) | <0.001 |
| Saint Lucia | 16  (7-31) | 47.33  (19.37-95.13) | 21  (9-41) | 45.18  (18.21-91.56) | -0.13 (-0.14 to -0.11) | <0.001 |
| Saint Vincent and the Grenadines | 15  (6-29) | 56.47  (23.4-109.85) | 14  (6-27) | 51.3  (21.12-100.9) | -0.31 (-0.33 to -0.29) | <0.001 |
| Samoa | 18  (7-35) | 49.11  (19.13-98.21) | 23  (10-45) | 48.34  (18.88-96.07) | -0.05 (-0.05 to -0.04) | <0.001 |
| San Marino | 3  (1-5) | 41.25  (16.23-82.18) | 3  (1-6) | 39.41  (15.72-80.27) | -0.13 (-0.15 to -0.12) | <0.001 |
| Sao Tome and Principe | 11  (4-21) | 42.18  (16.23-85.83) | 23  (10-45) | 41.88  (16.34-85.92) | -0.03 (-0.04 to -0.02) | <0.001 |
| Saudi Arabia | 1522  (630-2976) | 47.28  (18.96-94.41) | 4675  (1986-9006) | 45.62  (18.3-90.62) | -0.14 (-0.19 to -0.11) | <0.001 |
| Senegal | 577  (249-1087) | 34.17  (13.74-70.92) | 1313  (585-2454) | 34.14  (13.46-70.73) | 0 (-0.01 to 0) | 0.626 |
| Serbia | 1257  (542-2421) | 53.99  (22.13-105.55) | 1006  (436-1901) | 49.47  (20.09-97.46) | -0.27 (-0.34 to -0.21) | <0.001 |
| Seychelles | 7  (3-14) | 38.65  (14.88-78.87) | 9  (4-18) | 36.65  (14.47-75.07) | -0.17 (-0.18 to -0.16) | <0.001 |
| Sierra Leone | 474  (214-911) | 46.72  (19.27-94.44) | 911  (412-1743) | 41.05  (16.69-83.93) | -0.41 (-0.43 to -0.38) | <0.001 |
| Singapore | 232  (95-454) | 24.54  (9.35-49.87) | 326  (133-636) | 23.6  (9.01-48.17) | -0.12 (-0.14 to -0.1) | <0.001 |
| Slovakia | 687  (295-1304) | 51.78  (21.21-102.16) | 613  (269-1175) | 48.24  (19.21-95.39) | -0.23 (-0.24 to -0.21) | <0.001 |
| Slovenia | 243  (102-471) | 48.82  (20.13-98.03) | 196  (81-389) | 46.21  (18.04-93.52) | -0.17 (-0.18 to -0.16) | <0.001 |
| Solomon Islands | 37  (15-71) | 49.93  (19.86-100.02) | 84  (34-159) | 49.26  (19.06-98.56) | -0.02 (-0.05 to 0) | 0.038 |
| Somalia | 814  (337-1551) | 48.71  (19.67-95.82) | 2335  (972-4380) | 49.14  (19.51-97.43) | 0.03 (0.02 to 0.03) | <0.001 |
| South Africa | 3447  (1465-6663) | 36.51  (14.9-73.1) | 5138  (2188-9758) | 33.05  (13.42-66.47) | -0.31 (-0.33 to -0.29) | <0.001 |
| South Sudan | 550  (237-1064) | 43.19  (17.31-87.51) | 1031  (428-1973) | 45.2  (17.8-91.22) | 0.14 (0.13 to 0.15) | <0.001 |
| Spain | 4354  (1721-8455) | 45.26  (17.72-90.34) | 4271  (1791-8288) | 41.48  (16.24-83.46) | -0.28 (-0.29 to -0.26) | <0.001 |
| Sri Lanka | 1876  (784-3630) | 40.93  (16.33-82.96) | 2140  (898-4148) | 37.83  (14.74-77.15) | -0.25 (-0.26 to -0.24) | <0.001 |
| Sudan | 2203  (917-4164) | 47.37  (18.93-94.37) | 5122  (2151-9989) | 45.65  (18.22-91.54) | -0.12 (-0.12 to -0.11) | <0.001 |
| Suriname | 36  (15-70) | 37.37  (14.34-76.64) | 52  (21-100) | 35.54  (13.55-73.03) | -0.14 (-0.16 to -0.11) | <0.001 |
| Sweden | 735  (299-1420) | 35.77  (13.63-74.45) | 837  (351-1603) | 37.4  (14.76-75.41) | 0.16 (0.15 to 0.18) | <0.001 |
| Switzerland | 953  (408-1821) | 53.03  (21.68-103.98) | 1029  (442-1935) | 50.93  (20.68-99.23) | -0.12 (-0.13 to -0.11) | <0.001 |
| Syrian Arab Republic | 1261  (521-2444) | 46.22  (18.45-93.99) | 1735  (729-3395) | 44.83  (17.65-89.44) | -0.09 (-0.1 to -0.08) | <0.001 |
| Taiwan (Province of China) | 1076  (441-2066) | 19.23  (6.64-42.04) | 971  (397-1852) | 18.02  (6.29-39.26) | -0.17 (-0.21 to -0.12) | <0.001 |
| Tajikistan | 575  (241-1094) | 48.24  (19.6-97.55) | 1210  (512-2360) | 47.62  (19.59-94.59) | -0.04 (-0.05 to -0.04) | <0.001 |
| Thailand | 6362  (2674-12559) | 39.85  (15.62-80.5) | 6201  (2594-12215) | 37.75  (14.86-76.97) | -0.17 (-0.18 to -0.16) | <0.001 |
| Timor-Leste | 87  (38-170) | 47.18  (18.79-94.14) | 153  (63-290) | 44.75  (17.7-89.69) | -0.19 (-0.25 to -0.15) | <0.001 |
| Togo | 366  (152-689) | 43.06  (16.85-85.89) | 764  (339-1439) | 35.73  (14.15-73.11) | -0.58 (-0.61 to -0.56) | <0.001 |
| Tokelau | 0  (0-0) | 49.29  (19.61-98.3) | 0  (0-0) | 47.65  (18.68-94.44) | -0.1 (-0.11 to -0.09) | <0.001 |
| Tonga | 11  (5-20) | 48.72  (19.26-97.09) | 12  (5-23) | 47.75  (18.66-96.49) | -0.04 (-0.06 to -0.03) | <0.001 |
| Trinidad and Tobago | 143  (62-274) | 46.39  (18.68-93.37) | 122  (54-237) | 35.89  (14.1-74.89) | -0.8 (-0.82 to -0.78) | <0.001 |
| Tunisia | 907  (378-1790) | 44.57  (17.65-90.6) | 1321  (551-2562) | 42.82  (16.7-86.73) | -0.13 (-0.14 to -0.12) | <0.001 |
| Turkey | 6753  (2807-13237) | 47.76  (19.12-96.08) | 9777  (4182-18593) | 44.93  (17.89-90.1) | -0.19 (-0.21 to -0.16) | <0.001 |
| Turkmenistan | 406  (168-789) | 46.19  (18.5-92.73) | 565  (238-1105) | 44.91  (17.91-89.86) | -0.11 (-0.12 to -0.1) | <0.001 |
| Tuvalu | 1  (1-2) | 50.36  (20.26-98.83) | 1  (1-3) | 48.72  (19.28-96.95) | -0.08 (-0.11 to -0.06) | <0.001 |
| Uganda | 1252  (526-2387) | 32.86  (12.55-68.21) | 3302  (1336-6443) | 31.96  (12.05-66.05) | -0.1 (-0.14 to -0.06) | <0.001 |
| Ukraine | 4659  (1932-8993) | 36.55  (13.99-74.59) | 3763  (1553-7331) | 36.58  (14.01-74.38) | 0.01 (0 to 0.02) | 0.057 |
| United Arab Emirates | 137  (58-258) | 40.05  (15.56-81.72) | 699  (292-1354) | 40.09  (15.47-82.29) | -0.02 (-0.06 to 0.02) | 0.196 |
| United Kingdom | 5539  (2398-10672) | 38.61  (15.95-75.99) | 5598  (2468-10732) | 35.89  (15.01-70.53) | -0.24 (-0.24 to -0.23) | <0.001 |
| United Republic of Tanzania | 2525  (981-4851) | 42.76  (16.66-85) | 6210  (2686-11652) | 42.07  (16.43-84.46) | -0.05 (-0.07 to -0.04) | <0.001 |
| United States of America | 17962  (7668-34790) | 26.85  (10.39-54.38) | 19332  (8344-37791) | 25.69  (10.33-52.02) | -0.16 (-0.17 to -0.15) | <0.001 |
| United States Virgin Islands | 13  (6-26) | 45.82  (18.57-92.43) | 8  (3-15) | 43.47  (17.31-87.86) | -0.14 (-0.15 to -0.13) | <0.001 |
| Uruguay | 362  (150-701) | 48.28  (18.94-97.45) | 392  (168-744) | 46.54  (18.89-91.35) | -0.11 (-0.12 to -0.11) | <0.001 |
| Uzbekistan | 2338  (1006-4488) | 48.13  (19.37-95.27) | 4139  (1763-8020) | 46.2  (18.77-92) | -0.13 (-0.14 to -0.12) | <0.001 |
| Vanuatu | 17  (7-34) | 49.56  (19.5-98.12) | 39  (16-74) | 49.66  (19.55-97.29) | 0.01 (0 to 0.01) | 0.066 |
| Venezuela (Bolivarian Republic of) | 2203  (958-4216) | 46.38  (19.11-91.77) | 3215  (1404-6420) | 46.34  (18.86-93.16) | -0.02 (-0.04 to 0) | 0.042 |
| Viet Nam | 6633  (2762-12555) | 39.08  (15.24-79.72) | 8945  (3747-17161) | 34.95  (13.39-71.81) | -0.39 (-0.43 to -0.36) | <0.001 |
| Yemen | 1191  (491-2252) | 43.62  (17-88.21) | 3581  (1540-6886) | 43.11  (17.09-87.68) | -0.04 (-0.05 to -0.03) | <0.001 |
| Zambia | 762  (340-1419) | 41.42  (16.87-83.87) | 1786  (796-3458) | 36.9  (14.9-77.14) | -0.36 (-0.37 to -0.34) | <0.001 |
| Zimbabwe | 782  (317-1525) | 32.74  (12.33-67.19) | 1332  (539-2584) | 32.84  (12.14-69.04) | -0.01 (-0.05 to 0.02) | 0.383 |

AAPC: average annual percent change, ASR: age-standardized rate, DALYs: disability-adjusted life-years, WCBA: women of childbearing age.

Supplementary Table 5**.** The global case number and ASR of incidence, prevalence, and DALYs of caries among WCBA in 1990 and 2021 by age group, with AAPC from 1990 to 2021.

| **Age group** | **1990** | | **2021** | | **AAPC (95%CI)**  **1990–2021** | ***P*** |
| --- | --- | --- | --- | --- | --- | --- |
|  | **Number (95%UI)** | **ASR (95%UI)** | **Number (95%UI)** | **ASR (95%UI)** |  |  |
| **Incidence** | | | | | | |
| 15-19 | 103540805  (80808717-127366713) | 40518.62  (31622.87-49842.41) | 140061617  (110416944-171946753) | 46126.01  (36363.23-56626.63) | 0.42 (0.41 to 0.43) | <0.001 |
| 20-24 | 120651713  (96838053-144191450) | 49420.09  (39665.78-59062.19) | 159103116  (129038912-186549892) | 54162.39  (43927.84-63505.91) | 0.3 (0.28 to 0.31) | <0.001 |
| 25-29 | 99076165  (74636026-123310403) | 45014.25  (33910.12-56024.83) | 141648332  (108982499-173260348) | 48678.53  (37452.67-59542.24) | 0.27 (0.26 to 0.28) | <0.001 |
| 30-34 | 75414583  (57685742-91023935) | 39669.12  (30343.5-47879.86) | 125516531  (97867675-150164452) | 41988.48  (32739.23-50233.84) | 0.18 (0.17 to 0.2) | <0.001 |
| 35-39 | 59802452  (45842470-73268664) | 34477.55  (26429.29-42241.15) | 102680327  (79606221-124718994) | 36961.6  (28655.67-44894.8) | 0.24 (0.22 to 0.25) | <0.001 |
| 40-44 | 43341248  (33579189-55128254) | 30908.26  (23946.57-39314.01) | 81342942  (62458158-103323827) | 32787.69  (25175.62-41647.74) | 0.19 (0.18 to 0.19) | <0.001 |
| 45-49 | 31431060  (24827120-39428364) | 27619.4  (21816.32-34646.86) | 67127534  (53175163-84959260) | 28487.01  (22566.03-36054.29) | 0.1 (0.09 to 0.11) | <0.001 |
| **Prevalence** | | | | | | |
| 15-19 | 79894522  (54459796-111801383) | 31265.12  (21311.75-43751.23) | 94573293  (65610867-128797058) | 31145.5  (21607.4-42416.29) | -0.02 (-0.05 to -0.01) | <0.001 |
| 20-24 | 97253150  (70142633-126547494) | 39835.81  (28731.09-51835.05) | 112755202  (84784853-145084390) | 38384.48  (28862.73-49390.09) | -0.11 (-0.13 to -0.1) | <0.001 |
| 25-29 | 84670822  (58929561-122229035) | 38469.33  (26774.05-55533.52) | 110891761  (80929345-155877622) | 38108.8  (27811.99-53568.54) | -0.04 (-0.05 to -0.02) | <0.001 |
| 30-34 | 68155765  (49572503-95583460) | 35850.88  (26075.83-50278.23) | 105928518  (78487351-145778702) | 35435.79  (26256.02-48766.69) | -0.05 (-0.08 to -0.04) | <0.001 |
| 35-39 | 62140286  (40744651-84731527) | 35825.37  (23490.27-48849.76) | 101065010  (68828134-135735218) | 36380.14  (24775.9-48860.29) | 0.05 (0.04 to 0.06) | <0.001 |
| 40-44 | 50568440  (33593593-68633110) | 36062.24  (23956.84-48944.83) | 90703363  (61894010-122142650) | 36560.69  (24948.22-49233.23) | 0.05 (0.04 to 0.06) | <0.001 |
| 45-49 | 40824135  (28645422-54202943) | 35873.37  (25171.58-47629.72) | 83086581  (58728419-109304091) | 35259.58  (24922.67-46385.54) | -0.07 (-0.09 to -0.05) | <0.001 |
| **DALYs** | | | | | | |
| 15-19 | 79831  (30175-166333) | 31.24  (11.81-65.09) | 94468  (35981-196030) | 31.11  (11.85-64.56) | -0.02 (-0.04 to -0.01) | 0.014 |
| 20-24 | 96950  (36922-187566) | 39.71  (15.12-76.83) | 112360  (42934-218918) | 38.25  (14.62-74.52) | -0.11 (-0.13 to -0.1) | <0.001 |
| 25-29 | 83362  (34067-160568) | 37.87  (15.48-72.95) | 109223  (45601-207386) | 37.54  (15.67-71.27) | -0.03 (-0.05 to -0.01) | 0.004 |
| 30-34 | 66900  (27884-129104) | 35.19  (14.67-67.91) | 104036  (43533-203678) | 34.8  (14.56-68.14) | -0.05 (-0.07 to -0.04) | <0.001 |
| 35-39 | 61002  (25067-121937) | 35.17  (14.45-70.3) | 99210  (40325-197028) | 35.71  (14.52-70.92) | 0.05 (0.04 to 0.06) | <0.001 |
| 40-44 | 49271  (19321-96481) | 35.14  (13.78-68.8) | 88363  (35369-174198) | 35.62  (14.26-70.22) | 0.04 (0.03 to 0.05) | <0.001 |
| 45-49 | 39685  (16456-81238) | 34.87  (14.46-71.39) | 80770  (33744-165873) | 34.28  (14.32-70.39) | -0.07 (-0.09 to -0.05) | <0.001 |

AAPC: average annual percent change, ASR: age-standardized rate, DALYs: disability-adjusted life-years, WCBA: women of childbearing age.

Supplementary Table 6**.** The globally projected case number and ASR of caries among WCBA up to 2040

| **Year** | **Incidence** | | **Prevalence** | | **DALYs** | |
| --- | --- | --- | --- | --- | --- | --- |
|  | **Number** | **ASR** | **Number** | **ASR** | **Number** | **ASR** |
| 1992 | 553415949 | 39195.78413 | 500556688 | 36111.05562 | 493876 | 35.594225 |
| 1993 | 563057572 | 39259.95273 | 509188031 | 36106.68763 | 502337 | 35.59005 |
| 1994 | 572397060 | 39309.04342 | 517798807 | 36102.05184 | 510851 | 35.590905 |
| 1995 | 581735686 | 39346.3896 | 526560912 | 36096.97883 | 519443 | 35.586553 |
| 1996 | 589942112 | 39316.69861 | 535274111 | 36099.88513 | 528038 | 35.592646 |
| 1997 | 596361561 | 39196.21015 | 543622989 | 36116.56111 | 536308 | 35.613574 |
| 1998 | 602540144 | 39039.0555 | 552326177 | 36131.0394 | 544862 | 35.627866 |
| 1999 | 609154827 | 38913.21385 | 561061226 | 36157.49387 | 553529 | 35.657807 |
| 2000 | 617715906 | 38863.99912 | 570569397 | 36178.24075 | 562863 | 35.676501 |
| 2001 | 630162308 | 39066.23714 | 581690456 | 36323.28473 | 573858 | 35.821082 |
| 2002 | 647190370 | 39529.07788 | 595838368 | 36638.94582 | 587870 | 36.135997 |
| 2003 | 666074826 | 40087.30167 | 611093004 | 37011.48157 | 602940 | 36.504985 |
| 2004 | 684030289 | 40577.60918 | 625407988 | 37322.60748 | 617098 | 36.814155 |
| 2005 | 698095571 | 40838.18578 | 636596249 | 37451.60074 | 628183 | 36.944417 |
| 2006 | 709327174 | 40945.04745 | 644301699 | 37383.24274 | 635783 | 36.877639 |
| 2007 | 720505823 | 41065.70231 | 650189471 | 37219.67917 | 641603 | 36.71821 |
| 2008 | 731415167 | 41180.55099 | 655318120 | 37018.01313 | 646784 | 36.527795 |
| 2009 | 741620322 | 41274.13408 | 660532164 | 36838.76792 | 651937 | 36.353896 |
| 2010 | 750212669 | 41329.90653 | 666376967 | 36743.3188 | 657711 | 36.262591 |
| 2011 | 757828224 | 41386.51102 | 672712987 | 36726.23425 | 663894 | 36.244351 |
| 2012 | 765404828 | 41480.14825 | 678796545 | 36734.13929 | 669881 | 36.254015 |
| 2013 | 772477583 | 41591.72295 | 684269915 | 36754.69758 | 675219 | 36.27345 |
| 2014 | 778853904 | 41700.83441 | 689031716 | 36770.39227 | 679894 | 36.289819 |
| 2015 | 784474162 | 41789.30103 | 693098417 | 36768.14479 | 683824 | 36.285058 |
| 2016 | 790358489 | 41900.06476 | 694094139 | 36610.72185 | 684755 | 36.12884 |
| 2017 | 797061516 | 42060.79538 | 691718460 | 36284.50478 | 682421 | 35.808682 |
| 2018 | 803791584 | 42220.82194 | 689235249 | 35955.37617 | 679913 | 35.481769 |
| 2019 | 809655937 | 42324.50781 | 690132852 | 35799.9161 | 680729 | 35.325644 |
| 2020 | 815144014 | 42395.94068 | 693117562 | 35751.48028 | 683079 | 35.248059 |
| 2021 | 817480400 | 42286.71469 | 699003728 | 35842.42902 | 688430 | 35.314975 |
| 2022 | 821725686 | 42564.23013 | 690975871 | 35444.57289 | 681016 | 34.949182 |
| 2023 | 828783072 | 42666.37137 | 692395319 | 35284.43524 | 682340 | 34.78759 |
| 2024 | 836229200 | 42768.5126 | 694085534 | 35124.29759 | 683930 | 34.625997 |
| 2025 | 843461087 | 42848.4581 | 696597732 | 34992.80584 | 686354 | 34.494181 |
| 2026 | 850889673 | 42928.4036 | 699276240 | 34861.31409 | 688935 | 34.362366 |
| 2027 | 858705006 | 43008.3491 | 702159172 | 34729.82234 | 691716 | 34.23055 |
| 2028 | 866670345 | 43088.2946 | 705044079 | 34598.33059 | 694500 | 34.098735 |
| 2029 | 874328821 | 43168.2401 | 707520926 | 34466.83883 | 696883 | 33.966919 |
| 2030 | 881135835 | 43230.25069 | 710252887 | 34373.40975 | 699537 | 33.873528 |
| 2031 | 887567200 | 43292.26127 | 712561142 | 34279.98067 | 701776 | 33.780136 |
| 2032 | 893716469 | 43354.27186 | 714470806 | 34186.55158 | 703627 | 33.686745 |
| 2033 | 899349209 | 43416.28245 | 715963384 | 34093.1225 | 705064 | 33.593353 |
| 2034 | 904364035 | 43478.29303 | 717012651 | 33999.69342 | 706059 | 33.499961 |
| 2035 | 908268027 | 43526.52792 | 718329807 | 33950.28715 | 707325 | 33.450471 |
| 2036 | 911351226 | 43574.76281 | 718956903 | 33900.88089 | 707911 | 33.40098 |
| 2037 | 913969656 | 43622.9977 | 719114326 | 33851.47462 | 708037 | 33.35149 |
| 2038 | 916390451 | 43671.23258 | 719094493 | 33802.06836 | 707988 | 33.301999 |
| 2039 | 918652496 | 43719.46747 | 718942708 | 33752.66209 | 707811 | 33.252509 |
| 2040 | 920611441 | 43767.70236 | 718524835 | 33703.25583 | 707373 | 33.203018 |

ASR: age-standardized rate, DALYs: disability-adjusted life-years, WCBA: women of childbearing age.

Supplementary Table 7**.** Sensitivity analysis of the Nordpred age-period-cohort model: predictions of global caries cases among WCBA using original data and lower and upper bounds of case numbers.

| **Year** | **Case numbers of incidence** | | | **Case numbers of prevalence** | | | **Case numbers of DALYs** | | |
| --- | --- | --- | --- | --- | --- | --- | --- | --- | --- |
|  | **Original Data Prediction** | **Lower Bound Data Prediction** | **Upper Bound Data Prediction** | **Original Data Prediction** | **Lower Bound Data Prediction** | **Upper Bound Data Prediction** | **Original Data Prediction** | **Lower Bound Data Prediction** | **Upper Bound Data Prediction** |
| 1992 | 553415949 | 430689963 | 676452803 | 500556688 | 351219248 | 683334089 | 493876 | 198076 | 976524 |
| 1993 | 563057572 | 438526105 | 687363114 | 509188031 | 358604609 | 693000203 | 502337 | 201891 | 994117 |
| 1994 | 572397060 | 446209080 | 697967453 | 517798807 | 365991980 | 702708745 | 510851 | 205428 | 1010992 |
| 1995 | 581735686 | 453983479 | 709023981 | 526560912 | 373466336 | 712443360 | 519443 | 209256 | 1026973 |
| 1996 | 589942112 | 460436783 | 718923144 | 535274111 | 380123370 | 723864087 | 528038 | 212947 | 1043686 |
| 1997 | 596361561 | 465264088 | 727368937 | 543622989 | 386622593 | 734735460 | 536308 | 216340 | 1059951 |
| 1998 | 602540144 | 469795708 | 735648128 | 552326177 | 393172770 | 746004182 | 544862 | 219853 | 1075928 |
| 1999 | 609154827 | 474626223 | 744623727 | 561061226 | 399690647 | 757422930 | 553529 | 223271 | 1093223 |
| 2000 | 617715906 | 481031478 | 755920638 | 570569397 | 406804911 | 770234103 | 562863 | 227671 | 1110525 |
| 2001 | 630162308 | 490700423 | 771705088 | 581690456 | 415774859 | 783610712 | 573858 | 231883 | 1134547 |
| 2002 | 647190370 | 503790939 | 792228471 | 595838368 | 427735969 | 799721685 | 587870 | 237406 | 1160456 |
| 2003 | 666074826 | 518250281 | 814881090 | 611093004 | 440416251 | 817039049 | 602940 | 243703 | 1192200 |
| 2004 | 684030289 | 531909166 | 837578582 | 625407988 | 451710463 | 833725684 | 617098 | 249619 | 1220524 |
| 2005 | 698095571 | 542675312 | 856074750 | 636596249 | 460176296 | 847726800 | 628183 | 254203 | 1241717 |
| 2006 | 709327174 | 552037487 | 868716271 | 644301699 | 466072973 | 857667949 | 635783 | 257147 | 1252455 |
| 2007 | 720505823 | 561275798 | 881071016 | 650189471 | 470263535 | 865505803 | 641603 | 259533 | 1265833 |
| 2008 | 731415167 | 570463724 | 893117598 | 655318120 | 473965726 | 872785340 | 646784 | 261272 | 1275845 |
| 2009 | 741620322 | 578867930 | 904863297 | 660532164 | 477604134 | 880278551 | 651937 | 263349 | 1287342 |
| 2010 | 750212669 | 585909085 | 914852662 | 666376967 | 481690731 | 888619486 | 657711 | 266158 | 1296105 |
| 2011 | 757828224 | 591522592 | 923811212 | 672712987 | 486206166 | 897558823 | 663894 | 268625 | 1309137 |
| 2012 | 765404828 | 597290257 | 932787632 | 678796545 | 490318799 | 906509548 | 669881 | 270595 | 1318971 |
| 2013 | 772477583 | 602681187 | 941447818 | 684269915 | 494024509 | 914858691 | 675219 | 272768 | 1329260 |
| 2014 | 778853904 | 607353393 | 949537302 | 689031716 | 496873057 | 922119727 | 679894 | 274005 | 1341617 |
| 2015 | 784474162 | 611257873 | 956754747 | 693098417 | 499175970 | 928166697 | 683824 | 275817 | 1351708 |
| 2016 | 790358489 | 616289418 | 962753890 | 694094139 | 500139438 | 930539153 | 684755 | 276183 | 1351965 |
| 2017 | 797061516 | 622429757 | 969549277 | 691718460 | 497897073 | 929183990 | 682421 | 274981 | 1348202 |
| 2018 | 803791584 | 628211293 | 976771658 | 689235249 | 495325477 | 927597407 | 679913 | 273669 | 1345005 |
| 2019 | 809655937 | 633402484 | 983709940 | 690132852 | 495323270 | 929821155 | 680729 | 273700 | 1346923 |
| 2020 | 815144014 | 639458704 | 990815016 | 693117562 | 495424100 | 935585583 | 683079 | 275801 | 1350810 |
| 2021 | 817480400 | 641545574 | 994923526 | 699003728 | 499262978 | 942719732 | 688430 | 277488 | 1363111 |
| 2022 | 821725686 | 660768533 | 1022291530 | 690975871 | 505971359 | 957798896 | 681016 | 280925 | 1383905 |
| 2023 | 828783072 | 666598731 | 1030002063 | 692395319 | 506252421 | 960819460 | 682340 | 281276 | 1387347 |
| 2024 | 836229200 | 672479833 | 1037790353 | 694085534 | 506517383 | 963867061 | 683930 | 281617 | 1390819 |
| 2025 | 843461087 | 677955259 | 1045251895 | 696597732 | 507357969 | 967272419 | 686354 | 282160 | 1395187 |
| 2026 | 850889673 | 683454150 | 1052725132 | 699276240 | 508170278 | 970603971 | 688935 | 282679 | 1399433 |
| 2027 | 858705006 | 688968793 | 1060181815 | 702159172 | 508942166 | 973805090 | 691716 | 283167 | 1403474 |
| 2028 | 866670345 | 694532533 | 1067672261 | 705044079 | 509717407 | 976947438 | 694500 | 283644 | 1407433 |
| 2029 | 874328821 | 700108577 | 1075149018 | 707520926 | 510473043 | 980011707 | 696883 | 284101 | 1411291 |
| 2030 | 881135835 | 705201718 | 1082196281 | 710252887 | 511798072 | 983628535 | 699537 | 284838 | 1416090 |
| 2031 | 887567200 | 710062707 | 1088862130 | 712561142 | 512891646 | 986796460 | 701776 | 285446 | 1420206 |
| 2032 | 893716469 | 714711238 | 1095180676 | 714470806 | 513721763 | 989479253 | 703627 | 285910 | 1423578 |
| 2033 | 899349209 | 718615857 | 1100321257 | 715963384 | 513961502 | 991012515 | 705064 | 286047 | 1425218 |
| 2034 | 904364035 | 722136789 | 1104848617 | 717012651 | 513843272 | 991846536 | 706059 | 285983 | 1425816 |
| 2035 | 908268027 | 725063248 | 1108836539 | 718329807 | 514271820 | 993109922 | 707325 | 286191 | 1427372 |
| 2036 | 911351226 | 727763002 | 1112490349 | 718956903 | 514512054 | 994043709 | 707911 | 286298 | 1428413 |
| 2037 | 913969656 | 730366620 | 1116003187 | 719114326 | 514670771 | 994854005 | 708037 | 286360 | 1429231 |
| 2038 | 916390451 | 732621393 | 1119054656 | 719094493 | 514540934 | 995222698 | 707988 | 286278 | 1429356 |
| 2039 | 918652496 | 734484029 | 1121514564 | 718942708 | 514090245 | 995003785 | 707811 | 286018 | 1428584 |
| 2040 | 920611441 | 736105943 | 1123609525 | 718524835 | 513472169 | 994479115 | 707373 | 285668 | 1427354 |

DALYs: disability-adjusted life-years, WCBA: women of childbearing age.

Supplementary Table 8**.** Sensitivity analysis of the Nordpred age-period-cohort model: predictions of global caries among WCBA using original data and lower and upper bounds of ASR.

| **Year** | **ASIR** | | | **ASPR** | | | **ASDR** | | |
| --- | --- | --- | --- | --- | --- | --- | --- | --- | --- |
|  | **Original Data Prediction** | **Lower Bound Data Prediction** | **Upper Bound Data Prediction** | **Original Data Prediction** | **Lower Bound Data Prediction** | **Upper Bound Data Prediction** | **Original Data Prediction** | **Lower Bound Data Prediction** | **Upper Bound Data Prediction** |
| 1992 | 39195.78413 | 30488.38969 | 47966.56974 | 36111.05562 | 25312.48432 | 49267.30726 | 35.594225 | 14.310894 | 70.432999 |
| 1993 | 39259.95273 | 30563.627 | 47980.0314 | 36106.68763 | 25403.11438 | 49110.16701 | 35.59005 | 14.336887 | 70.496952 |
| 1994 | 39309.04342 | 30631.67573 | 47981.98885 | 36102.05184 | 25489.87706 | 48964.80793 | 35.590905 | 14.340102 | 70.488704 |
| 1995 | 39346.3896 | 30697.0393 | 48001.92379 | 36096.97883 | 25576.10624 | 48809.47667 | 35.586553 | 14.359738 | 70.41156 |
| 1996 | 39316.69861 | 30680.29168 | 47953.57828 | 36099.88513 | 25609.73213 | 48788.54305 | 35.592646 | 14.371308 | 70.404081 |
| 1997 | 39196.21015 | 30577.54801 | 47842.32168 | 36116.56111 | 25661.7018 | 48781.21529 | 35.613574 | 14.381464 | 70.433926 |
| 1998 | 39039.0555 | 30438.80504 | 47694.54687 | 36131.0394 | 25699.82091 | 48768.0007 | 35.627866 | 14.388588 | 70.396022 |
| 1999 | 38913.21385 | 30320.70289 | 47595.39044 | 36157.49387 | 25742.8028 | 48779.88511 | 35.657807 | 14.393804 | 70.448734 |
| 2000 | 38863.99912 | 30264.09533 | 47583.88995 | 36178.24075 | 25783.22614 | 48810.61714 | 35.676501 | 14.441122 | 70.398473 |
| 2001 | 39066.23714 | 30418.88499 | 47863.58925 | 36323.28473 | 25953.82 | 48909.80332 | 35.821082 | 14.485558 | 70.817949 |
| 2002 | 39529.07788 | 30767.38704 | 48409.22469 | 36638.94582 | 26295.38376 | 49159.31574 | 36.135997 | 14.606046 | 71.320025 |
| 2003 | 40087.30167 | 31186.82009 | 49063.57575 | 37011.48157 | 26671.50764 | 49474.52653 | 36.504985 | 14.76961 | 72.156991 |
| 2004 | 40577.60918 | 31548.79214 | 49705.51105 | 37322.60748 | 26957.86184 | 49750.78944 | 36.814155 | 14.908486 | 72.78473 |
| 2005 | 40838.18578 | 31738.71772 | 50097.86861 | 37451.60074 | 27076.22359 | 49874.35819 | 36.944417 | 14.969212 | 72.999819 |
| 2006 | 40945.04745 | 31853.56028 | 50164.01594 | 37383.24274 | 27045.35794 | 49772.73594 | 36.877639 | 14.936755 | 72.630052 |
| 2007 | 41065.70231 | 31973.0784 | 50236.06328 | 37219.67917 | 26921.18179 | 49564.11192 | 36.71821 | 14.875047 | 72.434555 |
| 2008 | 41180.55099 | 32096.98585 | 50304.76515 | 37018.01313 | 26771.0088 | 49330.62529 | 36.527795 | 14.775736 | 72.054229 |
| 2009 | 41274.13408 | 32191.52428 | 50378.40335 | 36838.76792 | 26629.48329 | 49129.54006 | 36.353896 | 14.703107 | 71.793381 |
| 2010 | 41329.90653 | 32252.9139 | 50416.39223 | 36743.3188 | 26549.6148 | 49035.21915 | 36.262591 | 14.686847 | 71.476483 |
| 2011 | 41386.51102 | 32281.47571 | 50460.02875 | 36726.23425 | 26532.92703 | 49034.74416 | 36.244351 | 14.673461 | 71.497484 |
| 2012 | 41480.14825 | 32351.88148 | 50551.69197 | 36734.13929 | 26523.41652 | 49082.17498 | 36.254015 | 14.646898 | 71.416135 |
| 2013 | 41591.72295 | 32438.95236 | 50682.69841 | 36754.69758 | 26525.30253 | 49155.58253 | 36.27345 | 14.651565 | 71.446328 |
| 2014 | 41700.83441 | 32515.58017 | 50826.46153 | 36770.39227 | 26505.44894 | 49215.294 | 36.289819 | 14.618093 | 71.646207 |
| 2015 | 41789.30103 | 32565.99651 | 50949.26488 | 36768.14479 | 26471.21155 | 49237.89392 | 36.285058 | 14.623082 | 71.761793 |
| 2016 | 41900.06476 | 32679.54101 | 51022.08805 | 36610.72185 | 26371.46538 | 49078.25942 | 36.12884 | 14.555134 | 71.370944 |
| 2017 | 42060.79538 | 32854.21768 | 51148.43303 | 36284.50478 | 26108.85979 | 48736.60334 | 35.808682 | 14.409387 | 70.77694 |
| 2018 | 42220.82194 | 33007.56607 | 51294.66291 | 35955.37617 | 25833.43742 | 48388.35024 | 35.481769 | 14.260923 | 70.211446 |
| 2019 | 42324.50781 | 33121.68006 | 51410.88843 | 35799.9161 | 25693.05216 | 48235.05933 | 35.325644 | 14.182636 | 69.905455 |
| 2020 | 42395.94068 | 33271.57427 | 51515.4183 | 35751.48028 | 25559.56461 | 48258.3086 | 35.248059 | 14.212879 | 69.711551 |
| 2021 | 42286.71469 | 33197.08979 | 51442.32217 | 35842.42902 | 25611.85646 | 48343.5914 | 35.314975 | 14.215167 | 69.918537 |
| 2022 | 42564.23013 | 33417.03098 | 51656.26594 | 35444.57289 | 25330.47877 | 47924.64615 | 34.949182 | 14.039917 | 69.270034 |
| 2023 | 42666.37137 | 33525.78345 | 51754.21185 | 35284.43524 | 25187.46478 | 47769.34936 | 34.78759 | 13.968092 | 68.993109 |
| 2024 | 42768.5126 | 33634.53593 | 51852.15776 | 35124.29759 | 25044.45078 | 47614.05256 | 34.625997 | 13.896267 | 68.716183 |
| 2025 | 42848.4581 | 33719.84142 | 51929.93498 | 34992.80584 | 24931.50621 | 47478.81482 | 34.494181 | 13.835616 | 68.486728 |
| 2026 | 42928.4036 | 33805.14692 | 52007.7122 | 34861.31409 | 24818.56164 | 47343.57708 | 34.362366 | 13.774965 | 68.257273 |
| 2027 | 43008.3491 | 33890.45241 | 52085.48942 | 34729.82234 | 24705.61707 | 47208.33933 | 34.23055 | 13.714314 | 68.027818 |
| 2028 | 43088.2946 | 33975.75791 | 52163.26664 | 34598.33059 | 24592.6725 | 47073.10159 | 34.098735 | 13.653662 | 67.798363 |
| 2029 | 43168.2401 | 34061.0634 | 52241.04386 | 34466.83883 | 24479.72793 | 46937.86385 | 33.966919 | 13.593011 | 67.568907 |
| 2030 | 43230.25069 | 34126.35184 | 52304.42098 | 34373.40975 | 24400.13352 | 46841.61948 | 33.873528 | 13.549383 | 67.403375 |
| 2031 | 43292.26127 | 34191.64028 | 52367.7981 | 34279.98067 | 24320.53911 | 46745.37512 | 33.780136 | 13.505754 | 67.237843 |
| 2032 | 43354.27186 | 34256.92872 | 52431.17522 | 34186.55158 | 24240.9447 | 46649.13075 | 33.686745 | 13.462126 | 67.072311 |
| 2033 | 43416.28245 | 34322.21716 | 52494.55235 | 34093.1225 | 24161.35028 | 46552.88639 | 33.593353 | 13.418498 | 66.906779 |
| 2034 | 43478.29303 | 34387.5056 | 52557.92947 | 33999.69342 | 24081.75587 | 46456.64203 | 33.499961 | 13.374869 | 66.741247 |
| 2035 | 43526.52792 | 34435.77242 | 52612.67231 | 33950.28715 | 24039.49032 | 46402.84476 | 33.450471 | 13.350359 | 66.653175 |
| 2036 | 43574.76281 | 34484.03924 | 52667.41515 | 33900.88089 | 23997.22477 | 46349.04749 | 33.40098 | 13.325849 | 66.565103 |
| 2037 | 43622.9977 | 34532.30606 | 52722.158 | 33851.47462 | 23954.95923 | 46295.25022 | 33.35149 | 13.301339 | 66.477031 |
| 2038 | 43671.23258 | 34580.57287 | 52776.90084 | 33802.06836 | 23912.69368 | 46241.45296 | 33.301999 | 13.276829 | 66.388959 |
| 2039 | 43719.46747 | 34628.83969 | 52831.64369 | 33752.66209 | 23870.42813 | 46187.65569 | 33.252509 | 13.252319 | 66.300887 |
| 2040 | 43767.70236 | 34677.10651 | 52886.38653 | 33703.25583 | 23828.16258 | 46133.85842 | 33.203018 | 13.227809 | 66.212815 |

ASR: age-standardized rate, ASIR: age-standardized incidence rate, ASPR: age-standardized prevalence rate, ASDR: age-standardized DALYs rate, DALYs: disability-adjusted life-years, WCBA: women of childbearing age.

Supplementary Figure 1 The global AAPC of incidence, prevalence, and DALYs for caries among WCBA from 1990 to 2021, by age group. AAPC: average annual percent change. DALYs: disability-adjusted life-years. WCBA: women of childbearing age.


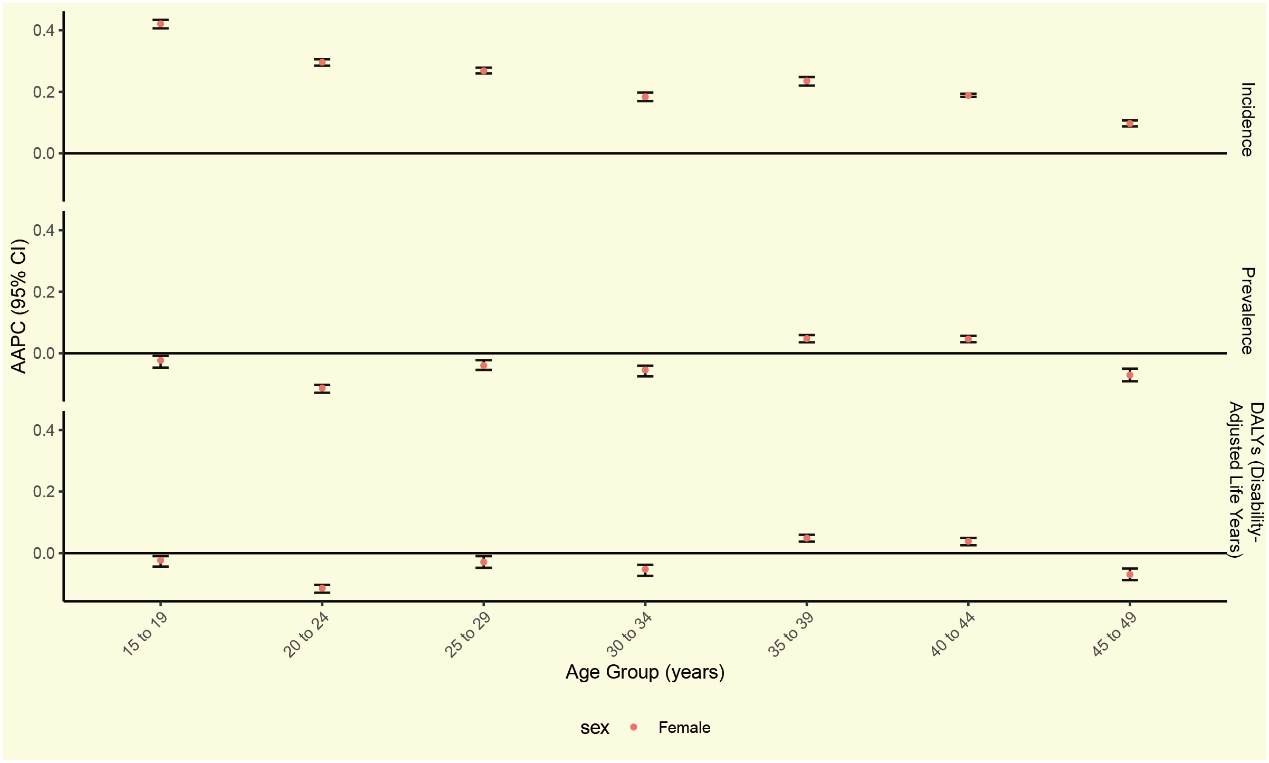


Supplementary Figure 2 The correlation between the SDI and the AAPC of incidence (A), prevalence (B), and DALYs (C) of caries among WCBA in 2021. AAPC: average annual percent change. DALYs: disability-adjusted life-years. SDI: sociodemographic index. WCBA: women of childbearing age.


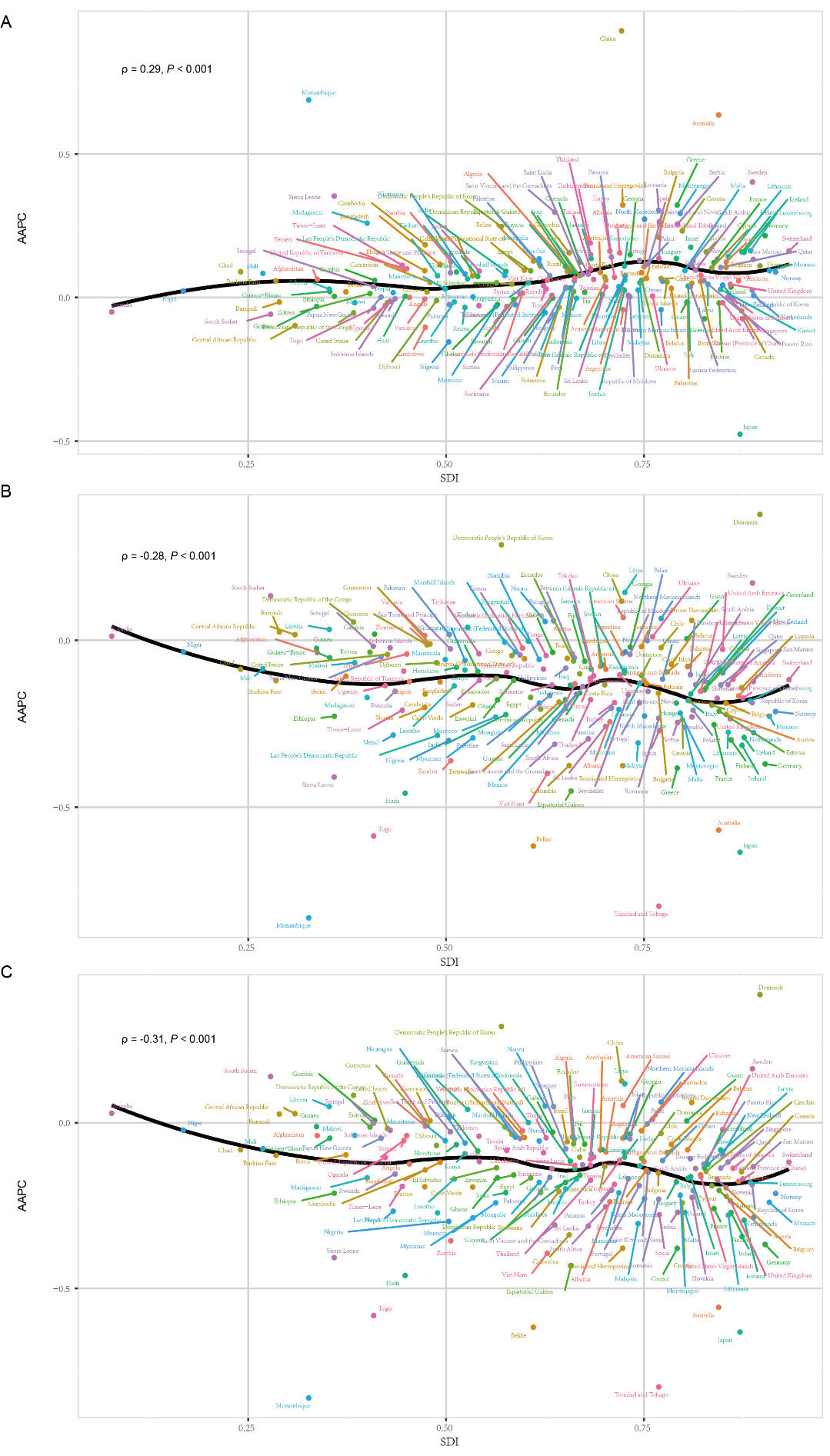

Supplement: Supplementary file 1 [file mmc1.docx]
